# Untargeted metabolomics for triaging of cytochrome *b* inhibitors during Chagas’ disease drug discovery

**DOI:** 10.1371/journal.pntd.0013917

**Published:** 2026-01-20

**Authors:** A. Kenneth MacLeod, Lindsay B. Tulloch, Michele Tinti, Darren Edwards, Susan Wyllie, Kevin D. Read

**Affiliations:** 1 Drug Discovery Unit, Wellcome Centre for Anti-Infectives Research, School of Life Sciences, University of Dundee, Dundee, United Kingdom; 2 Wellcome Centre for Anti-Infectives Research, School of Life Sciences, University of Dundee, Dundee, United Kingdom; FDA: US Food and Drug Administration, UNITED STATES OF AMERICA

## Abstract

Chagas’ disease, caused by infection with the protozoan parasite *Trypanosoma cruzi*, is a potentially fatal condition for which new treatments are urgently needed. Due to the lack of validated drug targets, phenotypic screening followed by target deconvolution is the dominant approach in Chagas’ disease drug discovery. However, as most phenotypic screening hits act through a small number of promiscuous targets, implementation of counter-screening methodology for these targets as early as possible in the workflow is essential to enable prioritisation of compounds with novel Modes of Action (MoA). Here, we demonstrate that untargeted metabolomic profiling using liquid chromatography mass spectrometry (LC-MS) can reliably identify compounds that act through one of the most common targets, cytochrome *b*. Treatment of epimastigote form *T. cruzi* in culture with cytochrome *b* inhibitors resulted in rapid and pronounced perturbation of the metabolome. We identified a signature of 79 metabolites that were differentially expressed by at least 2-fold (p < 0.05). Unsupervised multivariate analysis using these features allowed clear separation of cytochrome *b* inhibitors from compounds acting through other MoA, and through disruption of oxidative phosphorylation by other mechanisms. Flexibility was observed in this cytochrome *b* signature between experiments, and depending on the compounds used, suggesting that this approach could be readily implemented in other laboratories. Triage of cytochrome *b* inhibitors early in the Chagas’ disease drug discovery workflow using untargeted metabolomics will aid in prioritisation of medicinal chemistry resources towards compounds acting through novel mechanisms.

## Author summary

Chagas’ disease, a consequence of infection with the parasite *Trypanosoma cruzi*, is a severe global health problem. Most of the 6–7 million infected individuals reside in Latin America and increasing human migration is driving global dispersion. There are only two drugs available for treatment, both of which are poorly efficacious in the chronic phase of infection where progression to heart failure and sudden death is a frequent occurrence. New medicines that target novel aspects of parasite biology are urgently needed but most of the chemical start points identified in drug discovery projects are eventually found to act through similar mechanisms. Implementation of methodology to enable prioritisation of compounds with novel Modes of Action (MoA) as early as possible during drug discovery programmes is essential for the effective allocation of medicinal chemistry resource. To this end, we report here a MoA classification method which enables the identification and deprioritisation of compounds acting through one of the most common *T. cruzi* targets, cytochrome *b*, during Chagas disease drug discovery. The flexibility of this technique suggests that it could be readily implemented in other laboratories.

## Introduction

Approximately 6–7 million people are believed to be infected with *T. cruzi*, the majority of whom live in Mexico, Central and South America. This distribution is associated with the habitat of the vector host, the triatomine bug, but primarily due to increasing international travel approximately 75 million people are considered at risk of infection [1]. The acute stage of infection is characterised by non-specific symptoms such as fever, headache and muscle pain, with high parasite numbers present in the blood and multiple tissues, facilitating straightforward detection [2]. Two treatment options are available, benznidazole and nifurtimox, both of which are nitroheterocyclic drugs first deployed in the 1960s. Treatment with either of these drugs during the acute stage is curative in 80–90% of patients [3]. However, in most cases of infection, acute symptoms are mild or completely absent. The vast majority of individuals therefore remain undiagnosed, untreated, and progress to chronic infection. Parasitaemia is lower at this stage but is associated with reservoirs of “persister” parasites, dormant forms refractory to drug treatment and host immune response [4,5]. The World Health Organisation estimates that progressive tissue damage resulting from continuous low level inflammation leads to digestive and cardiac disorders in up to 10% and 30% of cases, respectively, with 12,000 deaths occurring per year, mostly due to heart failure decades after initial infection [1]. Benznidazole and nifurtimox are far less effective when administered in the chronic phase, contraindicated for some populations, and associated with high incidence (up to 40%) of adverse reaction in older patients [1]. There is therefore a pressing need to develop new medicines.

Drug discovery and development for Chagas’ disease has suffered from historic underfunding [6] however, in recent years, several charitable foundations and pharmaceutical companies have invested in early discovery [7]. In collaboration with academic groups researching kinetoplastid biology, substantial progress has been made in the definition of an effective workflow utilising new cellular and animal models. There are very few validated molecular targets to support target-based drug discovery against *T. cruzi*, and phenotypic screening has therefore proved an effective means to identify new chemical start points. However, further development and optimisation of phenotypic screening hit compounds can be impeded by a lack of knowledge of the Mode of Action (MoA). Target deconvolution – the process by which the molecular target is identified – is an essential component to allow a rational approach to optimisation of new molecules [8]. Crucially, through such work, it has become apparent that a very high proportion of phenotypic screening hits act through a relatively small number of targets. Hence implementation of target deconvolution methodology as early as possible in phenotypic drug discovery programmes is critical to allow deprioritisation of compounds acting through these promiscuous targets in favour of novel MoA. At the University of Dundee Drug Discovery Unit, we have observed that approximately 58% of high-throughput phenotypic screening hits against *T. cruzi* act through CYP51, a sterol 14α-demethylase essential for membrane function [9]. Because of this observation, we developed a high-throughput fluorescence-based assay with recombinant enzyme to enable routine triaging of CYP51 inhibitors [9]. The next most promiscuous target is the Q_i_ active site of cytochrome *b*, part of the cytochrome *bc1* complex of the electron transport chain, with a typical frequency of approximately 20% of phenotypic hits [10]. We have previously assembled a panel of *T. cruzi* clones bearing a variety of mutations in the cytochrome *b* gene, each of which confers varying susceptibility to cytochrome *b* inhibitors [10]. Compared to wild-type *T. cruzi*, a shift in potency of hit compounds in these lines (either resistance or hyper-sensitivity) indicates involvement of cytochrome *b*, and is therefore one way of triaging compounds.

Metabolomic profiling, where measurement of changes in abundance of endogenous small molecules within a biological sample in response to chemical challenge can indicate dysregulation of specific metabolic pathways, is a methodology well-suited to the identification and characterisation of drug MoA [11]. Although, in the context of anti-infective drug discovery and development, metabolomic profiling has been more widely used for biomarker and target discovery [12], there are many examples of successful target deconvolution studies in protozoan pathogens including kinetoplastids and *Plasmodium* [13–17]. Such work tends to involve significant effort in the appropriate design of individual experiments and in the manual curation and review of results such that their interpretation provides insight. For a metabolome profiling assay to be of sufficient utility during hit prioritisation in early drug discovery, where the aim is to identify the best compounds for progression as quickly as possible, standardised experimental methodology followed by analysis of metabolomic data using clustering approaches (with minimal data interpretation) is a more appropriate strategy [18]. Such an approach has been used to classify the MoA of the Medicines for Malaria Venture (MMV) “Malaria Box” of compounds in *Plasmodium falciparum*, the causative agent of malaria into one of four groups based on their disruptive effects: cellular homeostasis, haemoglobin catabolism, folate biosynthesis or mtETC/pyrimidine synthesis, with the remaining 35% unclassified [19]. Others have used metabolomics-based methodology to demonstrate that approximately half of the Malaria Box compounds elicit similar metabolic effects to existing drugs [20], to determine the life cycle stage at which drugs are most active [21], and to identify a motif shared among fast acting compounds [22]. MoA clustering approaches using metabolomic data have also been applied to differentiate the effects of antimicrobials in organisms including *Mycobacterium smegmatis* [23,24], *Escherichia coli* [25] and *Staphylococcus aureus* [26]. This preference for clustering approaches where broad classifications are used is in part a consequence of the inherent difficulty in identifying specific targets when the metabolome is incompletely sampled and the distinction between specific and generic effects cannot necessarily be determined. To an extent, multivariate and clustering analysis of the effects of multiple compounds used in parallel overcomes this, as non-specific effects are present in all samples. Although precise targets are rarely identified, rather generalised processes, this nonetheless provides a means by which to identify compounds acting through similar mechanisms and thereby facilitates compound triage and drug combination study design. Here we describe the development and validation of such an approach to triage inhibitors of cytochrome *b* during Chagas’ disease drug discovery.

## Results and discussion

### Metabolomic effects of the cytochrome *b* inhibitor, DDD01716002, are rapid and pronounced

**Fig 1:**
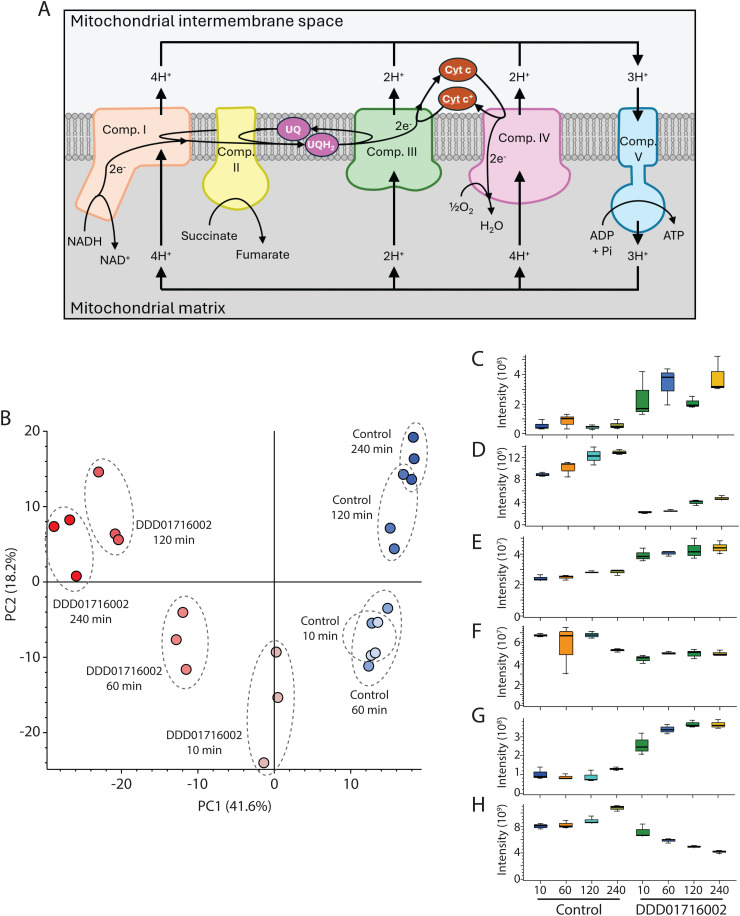
Effect of the cytochrome b inhibitor, DDD01716002, on the *T. cruzi* metabolome. (A) Oxidative phosphorylation scheme showing the five mitochondrial complexes with their reactions. Electrons (e^-^) are passed along the chain from complexes I and II to complex IV via ubiquinone (UQ) and cytochrome c (Cyt c) cofactors. Complexes I, III and IV export protons from the mitochondrial matrix to generate the proton gradient that complex V uses to drive ATP generation. (B) PCA shows result of triplicate sample analysis, with untreated cells in blue and DDD01716002-treated cells in red. Boxplots show LCMS response (peak area) of (C) succinate, (D) fumarate, (E) ADP, (F) ATP, (G) lactate and (H) proline.

Cytochrome b, the catalytic subunit of mitochondrial complex III, plays a central role in the electron transport chain and oxidative phosphorylation (Fig 1A). During the electron transport chain electrons are passed along a series of redox protein complexes (mitochondrial complexes I-IV) via ubiquinone/ol and cytochrome c cofactors to the terminal electron acceptor, oxygen. In addition complexes I, III and IV pump protons out of the mitochondrion, generating the protein gradient across the mitochondrial membrane (also known as the mitochondrial membrane potential) that drives ATP synthesis by complex V (FoF1-ATP synthase). This coupling of the electron transport chain with ATP synthesis is referred to as oxidative phosphorylation [27].

We first investigated the timecourse of metabolome changes following treatment with DDD01716002, an inhibitor of the Q_i_ active site of cytochrome *b*, at 2 × EC_50_ [10]. Samples were prepared from 10 ml cultures containing a total of 10^8^ *T. cruzi* epimastigotes for processing as described in Materials and Methods. Following LC-MS analysis, 885 features were observed across samples. Principal components analysis (PCA) revealed clear differentiation between treated and untreated cells after only 10 minutes, with this differentiation becoming more pronounced with increasing duration of incubation, out to the final sampling timepoint of four hours (Fig 1B).

Although the aim of this work was to determine whether metabolomic profiling could be used in compound triaging and, hence, mechanistic rationalisation of the observed effects was not required, we nonetheless scrutinised metabolites proximal to cytochrome b to assess whether expected changes were occurring. Unfortunately, cytochrome b substrates and products (ubiquinol, ubiquinone and cytochrome c) were not directly observed. However, the inhibition of complex III inhibits the entire electron transport chain by blocking the regeneration of ubiquinone (substrate of complex I and II) and reduction of cytochrome c (substrate of complex IV). Evidence of this blockage could be seen within 10 min of DDD01716002 addition to *T. cruzi* epimastigotes, with a rapid 4-fold accumulation of complex II substrate succinate and 4-fold depletion of complex II product fumarate (Fig 1C and 1D).

Inhibition of the electron transport chain inhibits oxidative phosphorylation as complexes I, III and IV are unable to generate the proton gradient that complex V uses to produce ATP. We observed a ~ 30% increase and decrease in ADP and ATP, respectively, within 10 min of DDD01716002 addition (Fig 1E and 1F), with these levels maintained for the following 4 hr, possibly as DDD01716002-treated cells switched to ATP generation by anaerobic means. Lactate, the anaerobic waste product of glycolysis, accumulated following DDD01716002 addition (Fig 1G), whereas levels of proline, an anaerobic energy source, steadily dropped over the subsequent 4 hr (Fig 1H). Representative chromatograms for each of these six metabolites are provided in S1 Fig.

### Identification of cytochrome b metabolomic signature

With throughput a key consideration, we sought to reduce culture and sample size. In all subsequent experiments, treatments were carried out with 1.4 ml cultures containing a total of 1.4 × 10^7^ cells, using 12-well culture plates. An incubation time of 4 hours was selected for all further work as (i) short sampling times were not practical when processing larger numbers of samples, (ii) slow-acting drugs may require longer incubation times to exert effect (for example, 24 hours are required for CYP51 inhibitors to alter the sterol profile significantly in *leishmania* [28] and (iii) this might allow cytochrome b inhibition effects to become apparent without further development to a more general profile associated with cell death. The incubation time of 4 hours therefore represented a compromise between all of these factors. Compound concentrations were increased to 5 × EC_50_ as this enhanced the level of target inhibition and was shown, in all cases, not to cause cell death by 4 hours.

To mimic a typical counter-screening experiment – which would require differentiation of cytochrome *b* inhibitors from a variety of other hit compounds acting through various (unknown) targets – and thereby to identify a common signature of cytochrome b inhibition, we carried out a small-scale experiment comparing the *T. cruzi* metabolome following treatment with four cytochrome *b* inhibitors to that of a “non-cytochrome *b* group”, comprised of samples from untreated cells, or from cells treated with inhibitors of other targets (Table 1).

**Table 1:** Compounds used to identify metabolic signature of cytochrome b inhibitors. All compounds were verified inhibitors of the targets specified in *T. cruzi.* KRS: lysyl-tRNA synthetase, NMT: N-myristoyltransferase.

Compound	Target	EC_50_ (nM)	Screen concentration		Reference
			nM	×EC_50_	
DDD01716002	Cyt b	76.5 ± 3.3	400	5	[10]
DDD01542111	Cyt b	180 ± 10.0	1,000	5	
DDD00808408	Cyt b	267 ± 3.0	1,500	6	
Compound 3	Cyt b	9.9 ± 0.2	35	4	[29]
DMU759	KRS1	13.3 ± 1.4	75	6	[30]
DDD85646	NMT	34,500 ± 1,800	75,000	2	[31]
TCMDC-143194	Proteasome	2,040 ± 35	15,000	7	[32]

**Fig 2:**
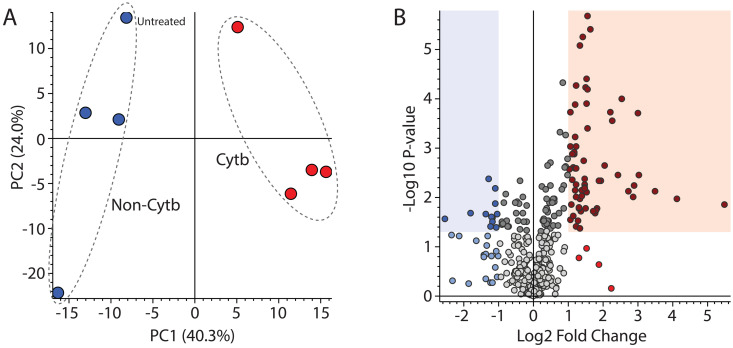
Pronounced and distinctive effect of cytochrome b inhibitors on the *T. cruzi* metabolome. (A) PCA comparing “Cytb” and “Non-Cytb” groups. (B) Volcano plot showing 83 features with ≥2-fold increase (red) or decrease (blue) in abundance (p = 0.05) between groups.

As expected due to the lower sample concentration in comparison to the previous experiment, a lower total number of features were observed. As this number was higher (467) with the MS operating in positive mode than in negative mode, the former was used here and for all subsequent analyses. Despite the diverse nature of the non-cytochrome *b* group, unsupervised multivariate analysis demonstrated clear differentiation of the two groups (Fig 2A).

In comparison of the group ratios for each feature by two-tailed student’s t-test to identify those metabolites which might be used as a more specific diagnostic signature of cytochrome *b* inhibition, we identified 83 that were differentially expressed by at least 2-fold (p < 0.05, Fig 2B). As approximately two thirds of these metabolites were lost (p > 0.05) following Benjamini-Hochberg multiple test correction, this was not applied during definition of the cytochrome *b* signature on the assumptions that (i) the p value of individual metabolites would vary between different experiments with different compounds and (ii) many features would likely demonstrate high coefficients of variation in this type of comparison due to the diversity of MoA within the “non-cytochrome *b*” group. Discarding of these features may therefore have diminished the capacity of multivariate analysis using this signature to differentiate cytochrome *b* inhibitors from other compounds as yet untested. S1 Table contains p values for each metabolite as calculated by two-tailed student’s T test, both before and after adjustment by multiple test correction. Following removal of four duplicate features, the cytochrome *b* signature list was defined as comprising 79 metabolites (Table 2). In accordance with Metabolomics Standards Initiative guidelines [33], annotation confidence level was associated with match to an in-house database of authentic reference standards (Level A), to the mzCloud spectral library (Level B), to the KEGG database (Level C) or in the absence of any database match (D). Three putative annotations from KEGG corresponding to drugs were rejected. Pathways analysis indicated metabolism of amino acids and nucleotides as prominent features of the cytochrome b signature (Tables 3 and S1). Some of the features used in the signature list had identical retention times. Specifically, there was a group at exactly 5.47 minutes and another group at exactly 5.94 minutes. While it is possible that these constitute in-source fragments of only two metabolites, this was not shown to be the case, hence none were removed from the signature list. Representative chromatograms, MS spectra and MS/MS spectra for each metabolite have been provided as S1 File.

**Table 2:** Cytochrome b signature list. Features showing differential abundance of ≥ 2-fold in *T. cruzi* treated with cytochrome b inhibitors. Levels A, B and C denote annotations assigned based on match to authentic reference standard, mzCloud or KEGG databases, respectively, while level D denotes metabolites to which putative molecular formulae could be defined based on exact mass, but for which database search returned no hits.

Formula	Name	Level	Calc. MW	m/z	RT [min]	Ratio: (Cytb)/ (Non-Cytb)
C10 H13 N4 O8 P	Inosine-monophosphate	A	348.0473	349.0546	6.52	45.27
C14 H27 N O4		D	273.1941	274.2013	3.26	17.67
C9 H14 O4	cis-2-Carboxycyclohexyl-acetic acid	C	186.0890	187.0962	3.65	11.26
C4 H10 Br Cl N7 O12 P		D	492.8995	493.9068	5.47	8.25
C15 H16 Cl N2 O15 P		D	529.9970	531.0043	5.47	8.08
C12 H23 N O4	Isovalerylcarnitine	C	245.1627	246.1700	3.65	7.36
C5 H2 N4 O	6-oxopurine	A	136.0385	137.0458	2.97	6.68
C3 H N7 O6 P2		D	292.9472	293.9545	5.55	5.85
C10 H22 Cl2 O17 P2		D	545.9715	546.9788	5.47	5.42
C4 H9 N O3	Homoserine	A	119.0581	120.0654	5.57	4.88
C5 H7 O2 P S		D	161.9905	162.9978	5.98	4.67
C10 H11 Cl N O13 P		D	418.9670	419.9742	5.47	4.13
C8 H3 O P S		D	177.9643	178.9717	5.98	3.84
C14 H17 N3 O S		D	275.1092	276.1165	3.29	3.73
C5 H11 N O2 S	Methionine	B	132.0244	150.0582	4.57	3.60
C19 H22 Cl N5 O18 S4		D	770.9519	771.9592	5.47	3.52
C10 H14 N5 O7 P	Adenosine monophosphate	A	347.0633	348.0706	6.14	3.44
C29 H17 Cl N5 O12 P3		D	754.9777	755.9850	5.47	3.29
C4 H8 N O P S		D	149.0066	150.0138	5.95	3.16
C4 H N O9		D	206.9648	207.9721	5.95	2.96
C2 H5 Hg	Ethyl mercury ion	C	231.0094	232.0167	5.94	2.96
C9 H18 N7 O17 P S		D	559.0226	560.0299	5.94	2.93
C3 H7 N O3	Serine	A	105.0423	106.0496	5.95	2.93
C7 H14 Cl N O P2 S2		D	288.9682	289.9755	5.95	2.89
C21 H16 Cl N4 O10 P3 S		D	643.9487	644.9560	5.47	2.88
C3 H4 Cl2 N2 O13 P2 S		D	439.8286	440.8359	5.46	2.87
C7 H12 N3 O7 P S		D	313.0129	314.0201	5.94	2.83
C11 H9 N O2	trans-3-Indoleacrylic acid	B	187.0631	205.0969	4.51	2.82
C11 H12 N2 O2	Tryptophan	A	204.0896	205.0968	4.51	2.82
C9 H11 N O3	Tyrosine	B	164.0474	182.0812	5.05	2.81
C10 H16 N2 O3 S	Biotin	B	244.0881	245.0954	1.33	2.81
C6 H2 N2 O12		D	293.9611	294.9684	5.47	2.79
C4 H5 Cl N3 P3		D	222.9388	223.9460	5.96	2.78
C4 H13 Cl N5 O7 P3		D	370.9714	371.9787	5.94	2.73
C6 H16 N5 O9 P3		D	395.0158	396.0231	5.94	2.71
C8 H9 N		D	119.0734	120.0807	4.17	2.67
C8 H6	[Similar to: Phenylethanolamine; ΔMass: -35.0372 Da]	B	102.0468	120.0807	4.17	2.66
C7 H19 Cl N O13 P3		D	452.9747	453.9820	5.94	2.65
C12 H16 N4 O S	Thiamine	B	264.1042	265.1114	4.55	2.62
C14 H26 N4 O11 P2	Citicoline	C	488.1080	489.1153	6.95	2.57
C9 H22 N O15 P3		D	477.0193	478.0265	5.94	2.56
C9 H11 N O2	Phenylalanine	B	148.0525	166.0863	4.17	2.55
C8 H Cl2 N4 O7 P		D	365.8958	366.9030	5.46	2.51
C9 H12 N2 O2	(4-Ethoxyphenyl)urea	C	180.0898	181.0971	1.32	2.50
C6 H13 N O2	Isoleucine	A	131.0947	132.1020	4.32	2.45
C5 H4 Cl N O12		D	304.9420	305.9492	5.95	2.37
C8 H14 N2 O5	gamma-Glutamylalanine	C	218.0901	219.0974	6.49	2.37
C13 H16 N2 O10 P2		D	422.0268	423.0341	5.92	2.37
C16 H5 Cl N2 P2 S		D	353.9341	354.9413	4.51	2.37
C5 H9 N2 O P S		D	176.0174	177.0247	5.93	2.36
C11 H10 Cl N5 O13 P2		D	516.9444	517.9516	5.46	2.35
C10 H21 N8 O15 P3		D	586.0339	587.0412	5.91	2.33
C14 H17 N3 O		D	243.1371	244.1444	3.17	2.33
C8 H11 N O9 S2		D	328.9869	329.9942	5.95	2.32
C7 H10 N O4 P		D	203.0348	241.9979	4.16	2.31
C4 H8 N2 O3	Asparagine	A	132.0535	133.0608	5.94	2.27
C5 H7 N8 O P S		D	258.0203	259.0276	5.92	2.27
C13 H9 Cl N2 O3		D	276.0310	259.0277	5.92	2.27
C3 H7 N O2	Alanine	A	89.0475	90.0548	5.47	2.23
C10 H15 N5 O10 P2	Adenosine 5’-diphosphate	A	427.0298	428.0371	7.42	2.19
C15 H10 Cl F3 N2 O3		D	358.0341	341.0309	5.92	2.18
C8 H13 N4 O7 P S		D	340.0236	341.0309	5.92	2.18
C4 H7 N3 O	Creatinine	A	113.0587	114.0660	2.87	2.13
C7 H23 N8 O8 P3 S2		D	504.0305	505.0378	5.91	2.12
C6 H13 N O2	Leucine	A	131.0947	132.1020	4.19	2.09
C15 H16 N2 O		D	240.1263	258.1601	3.06	2.09
C6 H10 Cl N4 O5 P S		D	315.9797	316.9870	5.92	2.08
C39 H14 N3 O3 P S2		D	667.0208	668.0280	6.92	0.49
C8 H12 Cl O16 P		D	429.9562	430.9635	6.51	0.48
C17 H27 N3 O17 P2	UDP-GlcNAc	A	607.0831	608.0905	6.92	0.48
C21 H22 N3 O15 P S3		D	682.9949	684.0021	6.92	0.47
C18 H37 N O2	Threo-sphingosine, (-)-	C	299.2825	300.2898	1.68	0.45
C15 H15 Cl N2 O4 S		D	354.0445	393.0077	6.51	0.44
C4 H7 N O4	Aspartate	A	133.0375	134.0448	6.58	0.44
C25 H14 N O13 P S		D	598.9924	599.9997	6.51	0.43
C5 H15 N6 O18 P3 S2		D	603.9088	604.9161	5.92	0.41
C3 H6 N3 P3 S		D	208.9491	209.9563	6.59	0.39
C11 H21 N2 O4 P3		D	338.0705	339.0778	6.51	0.29
C6 H15 N4 O5 P	Phosphoarginine	C	254.0781	255.0853	7.82	0.17

**Table 3:** Pathways analysis. Molecular pathways containing three or more metabolites from the cytochrome b signature.

Pathway	# metabolites
Superpathway of indole-3-acetate conjugate biosynthesis	5
Superpathway of L-isoleucine biosynthesis I	5
Aspartate superpathway	4
Superpathway of histidine, purine, and pyrimidine biosynthesis	4
Superpathway of L-threonine metabolism	4
Superpathway of purine nucleotide salvage	4
Superpathway of L-lysine, L-threonine and L-methionine biosynthesis II	3

### Validation of the cytochrome *b* signature

**Fig 3:**
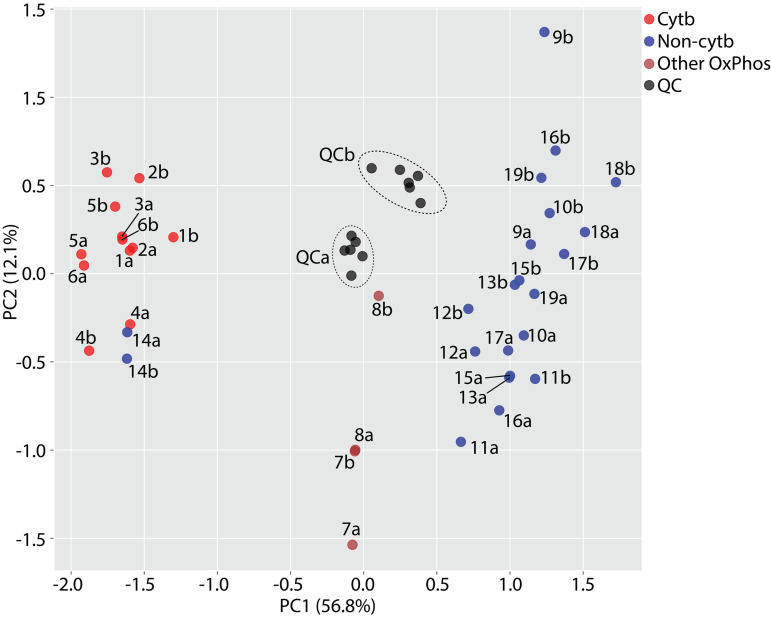
Validation of the cytochrome b signature. PCA scores plot shows cells treated with cytochrome b inhibitors (red), “non-cytochrome b inhibitors” (blue) or other inhibitors of oxidative phosphorylation (brown). QC pools (black) were prepared separately from replicate set a and replicate set b. Samples 18 and 19 were from untreated cells. All other samples were from cells treated with compounds as shown in Table 3.

To validate the cytochrome b signature for further use in the identification and triage of compounds acting through this target, cells were treated in duplicate with six cytochrome *b* inhibitors and 13 “non-cytochrome *b* inhibitors”, comprising 2 × untreated cells and 11x cells treated with well-characterised compounds known to act through other targets (Table 4). A large proportion of the metabolites identified in the previous experiment– 34 of the original 79 – were not observed. In the majority of cases this was due to their being of relatively low abundance, as evidenced by generally lower maximum peak area and higher relative standard deviation of peak area across QC injections (S1 Table). Consequently, many of these features did not meet processing method acceptance criteria. The remainder were missed due to their failing acceptance criteria for retention time or mass accuracy or, for a couple of level D metabolites, because a slight difference in m/z led to assignation of an alternative putative molecular formulae from that given in the previous analysis. but PCA using this subset of metabolites nonetheless allowed clear separation of cytochrome *b* inhibitors from almost all other treatments (Fig 3). Duplicate biological samples generally clustered closely together, indicating a high degree of reproducibility. Two QC pools were prepared, one from sample set “a” and the other from sample set “b”. There was some separation of these on principal component 1, indicating a degree of variability in the method, but this did not impede the clear separation of experimental groups.

**Table 4:** Compounds used to validate the cytochrome b signature. NMT: N-myristoyltransferase, PTR: pteridine reductase, DHFR-TS: dihydrofolate reductase-thymidylate synthase, NTR: nitroreductase, OSC: oxidosqualene cyclase, KRS: lysyl-tRNA synthetase, MRS: methionyl-tRNA synthetase, CPSF: cleavage and polyadenylation specificity factor. Note that NTR1 and NTR2 are not targets of nitroheterocyclic drugs but, rather, reductively activate the nitroheterocyclic prodrugs stated.

Compound	ID	Target	EC_50_ (nM)	Screen concentration		Species and reference
				nM	×EC_50_	
DDD01716002	1	Cyt b	76.5 ± 3.3	373	5	*T. cruzi* [10]
DDD01542111	2	Cyt b	180 ± 10	885	5	
Compound 5	3	Cyt b	21 ± 0.9	95	5	*T. cruzi*, series described in [29]
GNF7686	4	Cyt b	5,330 ± 310	27,000	5	*T. cruzi* [38]
Buparvaquone	5	Cyt b	33.7 ± 3.3	158	5	*L. donovani* [39,40]
Antimycin A	6	Cyt b	117 ± 4	585	5	*T. cruzi* [10]
Oligomycin A	7	Mitochondrial complex V	84.8 ± 7.1	397	5	[34]
2,4-dinitrophenol	8	Protonophore	175,000 ± 3,700	880,000	5	[35]
N,N,N′,N′-Tetrakis(2-pyridinylmethyl)-1,2-ethanediamine	9	Me2 + chelator	5,450 ± 250	28,000	5	*L. donovani* [41]
DDD01012248	10	Proteasome	117 ± 3	595	5	*T. cruzi* [32]
DDD85646	11	NMT	34,500 ± 1,800	100,000	3	*T. cruzi* [42]
Pyrimethamine	12	PTR1/ DHFR-TS	45,300 ± 8,800	100,000	2	*T. brucei* [43]
Fexinidazole sulfone	13	NTR1	18,600 ± 600	100,000	5	*T. brucei* [44]
GSK2920487A	14	OSC	4,070 ± 170	20,000	5	*L. donovani* [45]
DDD01510706	15	KRS	2,190 ± 200	10,000	5	*T. cruzi* [30]
DDD00806905	16	MRS	66.1 ± 2.2	550	8	*L. donovani* [46]
Acoziborole	17	CPSF3	29,200 ± 2,500	100,000	3	*T. brucei* [47]

Notably, two of the compounds tested in this validation experiment disrupted oxidative phosphorylation through mechanisms other than cytochrome *b* inhibition; oligomycin A is an ATP synthase inhibitor [34] and 2,4-dinitrophenol is a protonophore [35]. Samples from *T. cruzi* treated with these two compounds were observed in a distinct region of the PCA plot, separate from the cytochrome *b* inhibitor and “non-cytochrome *b* inhibitor” groups. The primary effect of compounds targeting cytochrome b is inhibition of the ETC and proton export from the mitochondrion; the secondary effect is inhibition of ATP synthase as the mitochondrial membrane potential depletes. There was no obvious mechanistic rationale for most of the metabolites responsible for sample group separation (S2 Table), supporting the unbiased approach used. However, the strongest drivers of separation of these two compounds from cytochrome b inhibitors were inosine monophosphate (23-fold lower, adjusted p = 8.99 x 10^-5^) and C12H23NO4 (11-fold lower, adjusted p = 8.97 x 10^-5^), putatively annotated as isovalerylcarnitine. The former is elevated during ETC/TCA shutdown due to depletion of aspartate and NAD + , preventing conversion to AMP or GTP, respectively [36]. The latter may be elevated during ETC inhibition due to accumulation of isovaleryl-CoA, which may be prevented from transferring electrons to 3-methylcrotonyl-CoA due to depletion of ubiquinone. This indicated that both oligomycin A and DNP failed to inhibit the ETC. The strongest driver of separation of these two compounds from the “non-cytochrome b inhibitor” group was C6H15N4O5P (2.7-fold lower, adjusted p = 0.02), putatively annotated as phosphoarginine. This metabolite is used as a phosphate store at times of ATP excess and is conversely depleted during ATP starvation [37], as occurs during inhibition of ATP synthase. Therefore, our results suggest that distinct clustering of these two compounds was due to the partial similarity of their effects to those of cytochrome b inhibitors, i.e., inhibition of ATP synthase without disruption of the ETC.

**Fig 4:**
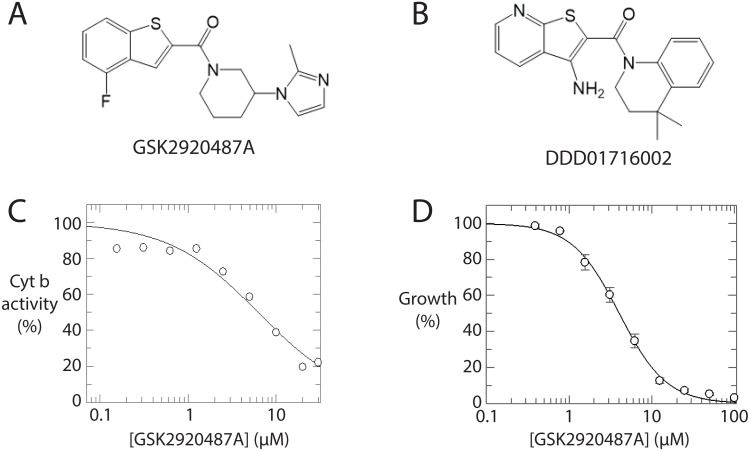
Investigation of GSK2920487A as a *T. cruzi* cytochrome b inhibitor. (A) Structure of GSK2920487A. (B) Structure of DDD01716002. (C) Dose-response curve for GSK2920487A against *T. cruzi* cytochrome b. Representative curve is shown for one biological replicate, composed of one technical replicate. (D) Dose-response curve for GSK2920487A against wild-type *T. cruzi* epimastigotes. Representative curve is shown for one biological replicate, composed of two technical replicates.

There was one “non-cytochrome b inhibitor” which clustered with the cytochrome b inhibitor group. This compound, GSK2920487A, has previously been shown through multiple orthogonal techniques to be an inhibitor of oxidosqualene cyclase in *Leishmania donovani* [45]. However, this has not been confirmed as the target in *T. cruzi*. Notably, GSK2920487A and DDD01716002 share a degree of structural similarity (Fig 4A and 4B). The former was therefore tested in an enzymatic assay against *T. cruzi* cytochrome b. Remarkably, GSK2920487A inhibited *T. cruzi* cytochrome b by 77% at 20 µM – the concentration used in the metabolomic assay – and displayed a weighted mean IC_50_ from three biological replicates of 7.3 ± 0.9 µM (Fig 4C). This, in conjunction with the observation that the EC_50_ against live *T. cruzi* epimastigotes is 4.0 ± 0.2 µM (Fig 4D), indicated that cytochrome b is a target of GSK2920487A in *T. cruzi* epimastigotes. However, it must be stressed that cytochrome b is not believed to be the target in the intracellular amastigote form of *T. cruzi*, the form in infected humans, where the potency of GSK2920487A is 10-fold greater. Oxidosqualene cyclase, the likely target in *T. cruzi* amastigotes as in *L. donovani*, plays a key role in sterol biosynthesis. The epimastigote form of *T. cruzi* can scavenge sterols from its surroundings whereas the amastigote form cannot, therefore the effect of oxidosqualene cyclase inhibition is of less consequence in the former. These observations underscore the importance of considering life-cycle stage when assessing compounds with different MoA.

The identification of GSK2920487A as a cytochrome b inhibitor in *T. cruzi* epimastigotes was an unexpected outcome from this study and served as an important validation of the workflow. With further development, the applicability of this methodology could be extended to any target for which an inhibition signature can be defined. A key limitation is the availability of verified inhibitors to enable the initial signature definition, and to then serve as positive controls in subsequent experiments. As this workflow is developed, however, the untargeted format in which the data are collected is well-suited to the identification of compounds with previously unrecognised or multiple mechanisms of action, either through prospective or retrospective analysis.

New antichagasic compounds are often identified by screening against mammalian cells infected with *T. cruzi* amastigotes, the clinically relevant parasite form [10,30,32]. However, determination of compound MoA in this context is challenging as host cell biomass greatly exceeds that of the parasite. As epimastigote *T. cruzi* grows axenically in culture, MoA elucidation studies are often carried out with this alternative form on the basis that most cellular processes are active in both forms, and the targets of most compounds are likely the same [10,30,32]. However, as we have shown here, results are not always comparable. Significant differences in drug potency between epimastigote and intracellular amastigote forms may be an indicator of target stage-specificity. Alternatively, the target may be the same but apparent potency could differ due to altered drug uptake or efflux. In certain situations, it may therefore be worth confirming metabolomic results in low-throughput orthogonal assays with intracellular parasites overexpressing the putative target [30,32], or *in vitro* biochemical assays such as described here. Although the requirement for additional steps to mechanically lyse host cells and to isolate and purify the live parasites released would decrease throughput and allow time for metabolic changes to occur during processing, [48,49], future assessment of whether this metabolomic approach is adaptable to analysis of *T. cruzi* intracellular amastigotes warrants investigation.

## Concluding remarks

Here we have demonstrated the utility of untargeted metabolomic profiling for the triage of phenotypic screening hits acting through cytochrome b inhibition, one of the most promiscuous targets in *T. cruzi*. Using multivariate methods to identify metabolite features that associated most strongly with inhibition of this target – irrespective of their identity or functional significance – we have defined a characteristic metabolomic signature. The original 79 metabolite signature was based on a small-scale experiment comparing only four cytochrome *b* inhibitors to four “non-cytochrome b inhibitors” and, as such, was highly dependent on the action of these compounds. Had different compounds been used, a somewhat different signature might have been identified. Nonetheless, a subset of this signature was highly effective in differentiating cytochrome *b* inhibitors from other compounds in an independent data set, demonstrating good technical reproducibility and an ability to differentiate cytochrome b inhibitors from compounds acting against oxidative phosphorylation through other mechanisms. Furthermore, although a minimum of three biological replicates are generally recommended for metabolomic studies to ensure statistical robustness, with five replicates preferred [33], this workflow permits effective clustering of cytochrome b inhibitors through unsupervised multivariate analysis with a single replicate for each compound, enabling efficient triaging of large compound sets. Repeated application of this methodology in future studies may allow iterative refinement towards an optimal signature list, but we have shown that inter-experimental variability in the metabolites used is not a barrier to effective application of the technique. Moreover, this innate flexibility suggests that profiling of polar metabolites in *T. cruzi* to identify cytochrome b inhibitors is a strategy that will be successful in other laboratories without extensive method development, facilitating an effective counter-screen for use during Chagas’ disease drug discovery.

## Materials and methods

### Chemicals and reagents

All LCMS mobile phase reagents were of LCMS grade and purchased from Fisher Scientific (Thermo Fisher Scientific, Waltham, MA, USA). Metabolite authentic standards were as supplied in IROA Technologies Mass Spectrometry Metabolite Library (lot 230–01, Merck, Burlington, MA, USA) and were reconstituted following manufacturer recommendations.

### Cell lines and culture conditions, drug sensitivity assays

*T. cruzi* epimastigotes of the Silvio strain (MHOM/BR/78/Silvio; clone X10/7A) were maintained at 28^o^C in RTH/FBS growth medium as previously described [32]. To determine the effects of compounds on cell growth, mid-log phase *T. cruzi* epimastigotes were seeded into 96-well plates with test compound concentration gradients established through two-fold serial dilutions, and cell viability was determined through cellular oxidation of resazurin as previously described [32]. Data were corrected for background fluorescence and fitted to the two-parameter equation below, where *y* is % cell growth, *[I]* is the inhibitor concentration and *m* is the slope, using Grafit (Erithacus Software).


$$y=\frac{\text{1}\text{0}\text{0}}{\text{1}+{\left(\frac{\left[I\right]}{{\text{EC}}_{\text{50}}}\right)}^{m}\;}$$

Weighted means were calculated for each drug from three biological replicates. Measurement of cytochrome b activity was performed in the presence and absence of inhibitors as previously described [10]. Data were fitted to the two-parameter equation above and a weighted mean IC_50_ was determined from three biological replicates.

### Sample preparation

Samples were prepared following the method first described by t’Kindt and colleagues for promastigote form *L. donovani*, as this effectively disrupts the glycocalyx – a robust pericellular matrix rich in glycoprotein and glycolipid – and extracts intracellular metabolites into a single-phase sample suitable for HILIC-MS analysis [50]. Briefly, *T. cruzi* epimastigotes were seeded in RTH/FBS growth medium at 10^7^ cells/ml in 1.4 ml or 10 ml volumes and treated with inhibitors for 10–240 min as indicated. Cultures were rapidly cooled to 4 °C on dry ice/ EtOH to quench cellular metabolism. Cells were pelleted, washed 2× in 1 ml PBS and suspended in either 50 µl (cells from 1.4 ml culture) or 200 µl (cells from 10 ml culture) of CHCl3: MeOH: water (1:3:1). Metabolites were extracted with end-over-end agitation at 4 °C for 1 h. Insoluble material was pelleted at 16K g for 5 min at 4 °C and supernatants were transferred to glass vials for immediate analysis.

### Metabolomics

Samples (5 µL injection volume) were analysed using a Vanquish UHPLC system coupled to an Orbitrap Exploris 120 mass spectrometer, operated using Xcalibur software version 4.4.16.14 (Thermo Fisher Scientific). Chromatographic separation was achieved using an Atlantis Premier zic-HILIC column, 100 × 2.1 mm, particle size 1.7 µm (Waters, Milford, MA, USA, part number 186009979). The column was held at 45 °C, mobile phase A was 95/5 acetonitrile/water with 10 mM ammonium acetate and acetic acid to pH 5.0, mobile phase B was 50/50 acetonitrile/water with 10 mM ammonium acetate and acetic acid to pH 5.0. All mobile phase components were of LCMS grade. The gradient programme was as follows: 0.0 – 1 min: 1% B, 1 – 9 min: 1–98% B, 9–11 min: 98% B, 11 – 11.2 min: 98–1% B, and 11.2 – 15 min: 1% B. Flow rate was 0.6 mL/min. The mass spectrometer was operated with electrospray ionisation in positive mode for all analyses, with the exception of the DDD01716002 timecourse experiment where negative mode was used. MS parameters were as follows: ion source type: H-ESI, spray voltage: static, positive ion (V): 3500, negative ion (V): 2500, Orbitrap resolution: 60000, scan range (m/z): 70–900, stepped HCD collision energies (%): 30,60,90. Data processing was carried out in Compound Discoverer version 3.3.3.200 (Thermo Fisher Scientific). Where possible, metabolite annotation was based on matching of chromatographic retention time, exact mass (+/- 3 ppm) and fragmentation spectrum with those of authentic standards for 643 metabolites previously analysed on the system, using mzVault version 2.3 (Thermo Fisher Scientific) and a defined mass list. Alternatively, putative annotations were assigned following search against mzCloud (Thermo Fisher Scientific) and KEGG databases. All metabolites discussed individually in the text were matched to an in-house database entry with the exception of lactate, which was matched to an entry in mzCloud. Pathways analysis was carried out using the Metabolika node in Compound Discoverer. Pathways considered biologically implausible, for example peptidoglycan or chitin biosynthesis, were excluded. LC-MS metabolomics data have been deposited in the EMBL-EBI MetaboLights database [51] with study identifier MTBLS13510.

### Data processing and statistical analysis for cytochrome b signature validation

Mass spectrometry-derived peak areas were obtained using Compound Discoverer and transferred to a custom computational pipeline implemented in Python (v3.7) for statistical analysis. Peak areas were log10-transformed and quantile normalised. Principal Component Analysis (PCA) was performed on all 45 metabolites using the sklearn Python package (0.22). For comparison of the effects of DNP and oligomycin A to those of other compounds, p values were calculated in Compound Discoverer using a one-way ANOVA model with Tukey post-hoc test and adjusted using Benjamini-Hochberg correction for false discovery rate.

## Supporting information

S1 Table Observations (m/z, retention time, reference ion) and metabolite annotation (calculated MW, molecular formula, name and annotation level) along with grouped peak areas, coefficients of variation (%CV), and resulting fold change and statistical test calculation results for the cytochrome b signature. Column T contains full pathways analysis results for each metabolite. Column Y denotes whether each was observed in the subsequent validation experiment.
(XLSX)

S2 Table Metabolites observed in the validation experiment alongside grouped peak areas, %CV and corresponding ratios/ fold changes between groups with statistical test results. (XLSX)

S1 Fig Representative chromatograms from analysis of QC injection for (A) succinate, (B) fumarate, (C) ADP, (D) ATP, (E) lactate and (F) proline. (EPS)

S1 File Representative chromatograms, MS1 and (where obtained) MS2 spectra for the cytochrome b signature. (PDF)
