# Supplementary material for: Untargeted metabolomics for triaging of cytochrome b inhibitors during Chagas’ disease drug discovery
Source: PLoS Negl Trop Dis. 2026 Jan 20;20(1):e0013917. doi: 10.1371/journal.pntd.0013917 (PMC12844530; doi:10.1371/journal.pntd.0013917)
Supplement: S1 File — (PDF) [file pntd.0013917.s004.pdf]

Compounds

17-Dec-2025 1:28

File name: 02_Cytb signature generation-(1)

Study: 02_Cytb signature generation

Structure	Name	RT [min]	Formula	Calc. MW	Group Areas
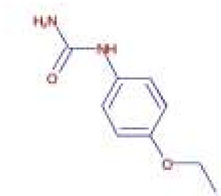	(4-Ethoxyphenyl)urea	1.32	C9 H12 N2 O2	180.08979	8.33e6 / 3.33e6

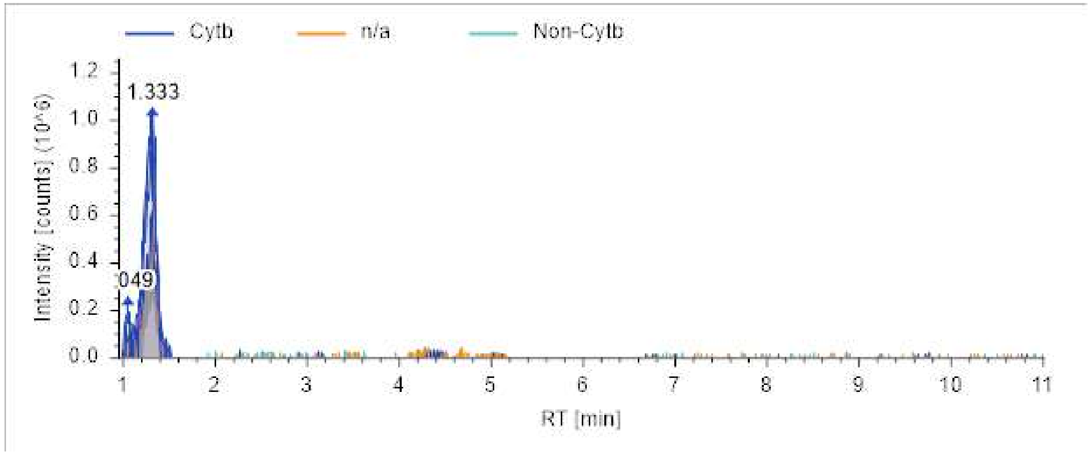

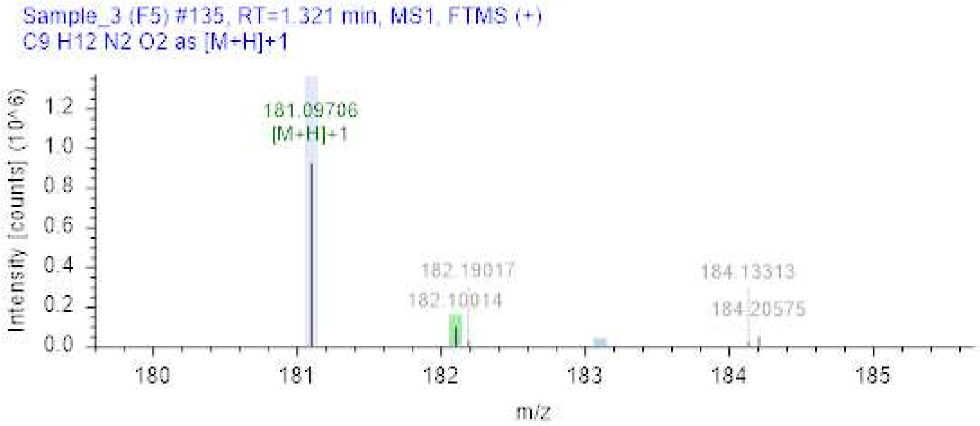

Compounds

17-Dec-2025 1:28

File name: 02_Cytb signature generation-(1)

Study: 02_Cytb signature generation

Structure	Name	RT [min]	Formula	Calc. MW	Group Areas
	Biotin	1.32	C10 H16 N2 O3 S	244.08812	6.72e7 / 2.39e7

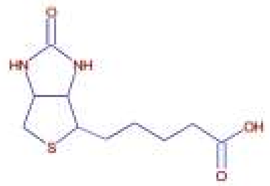

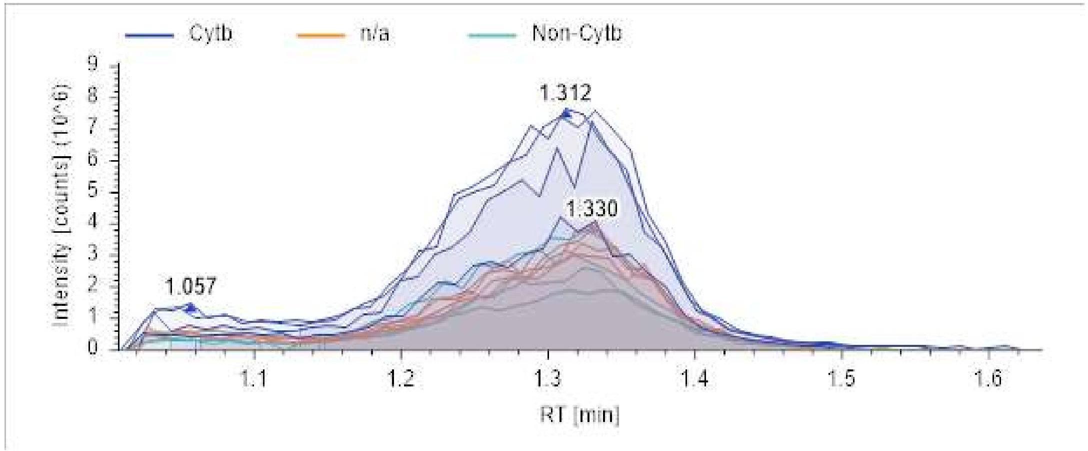

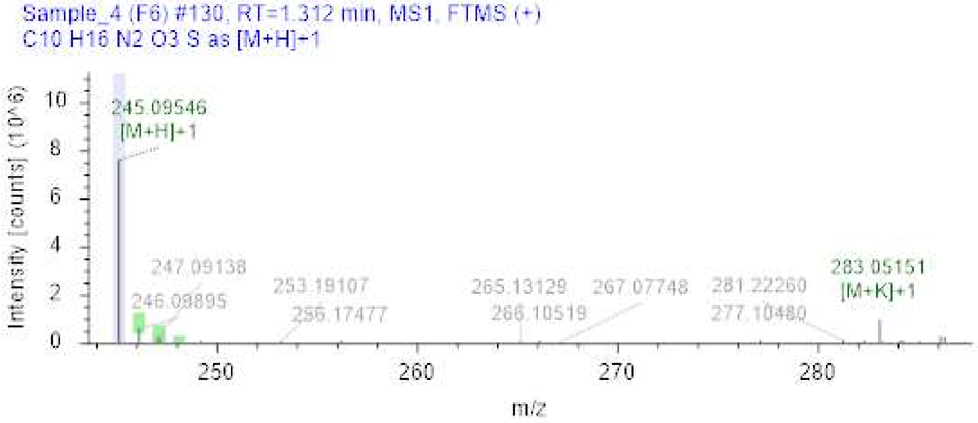

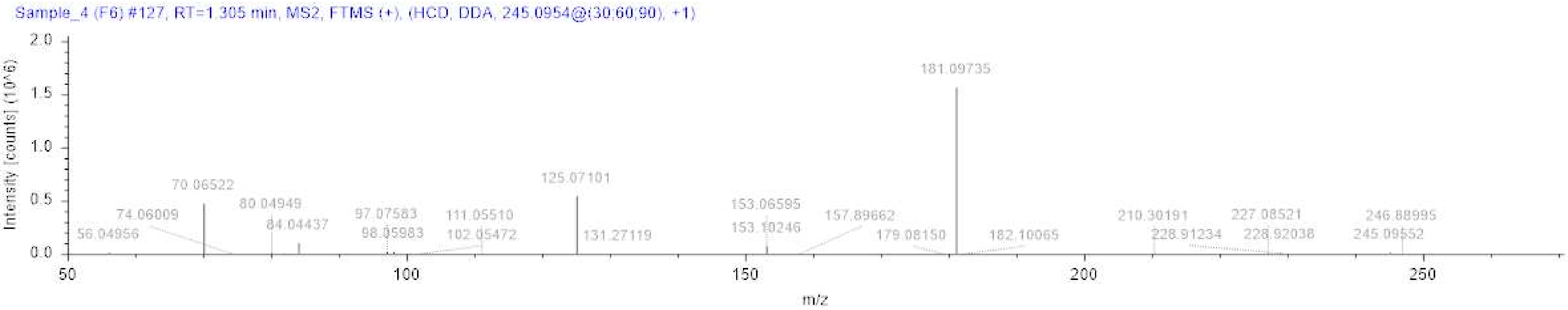

Compounds

17-Dec-2025 1:28

File name: 02_Cytb signature generation-(1)

Study: 02_Cytb signature generation

Structure	Name	RT [min]	Formula	Calc. MW	Group Areas
	THREO-SPHINGOSINE, (-)-	1.68	C18 H37 N O2	299.28254	1.12e7 / 2.48e7

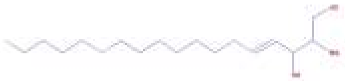

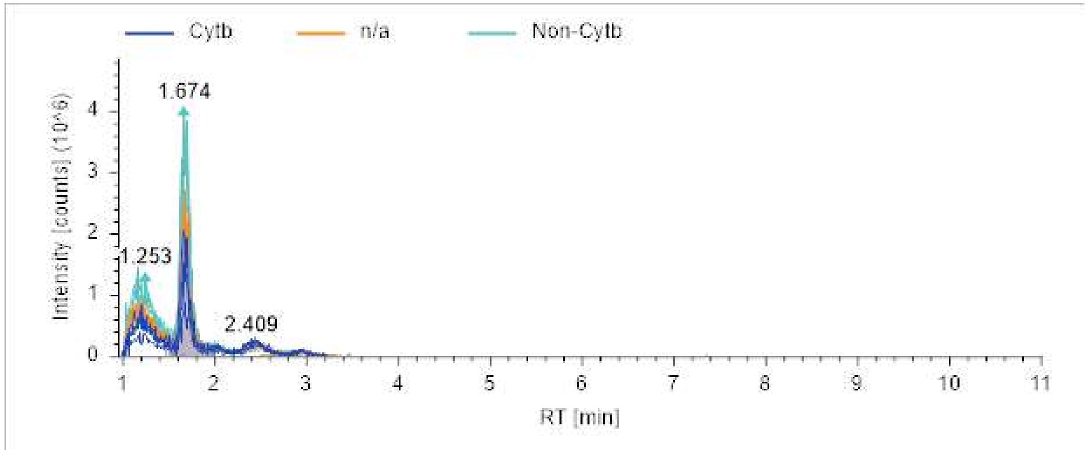

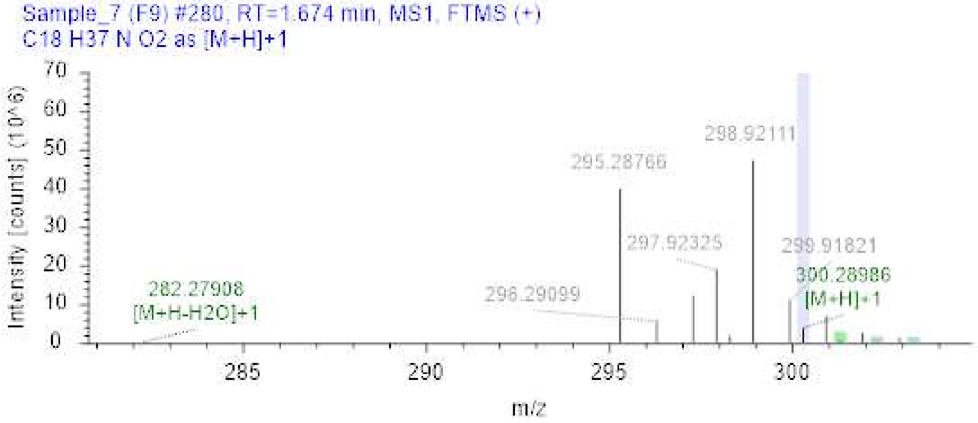

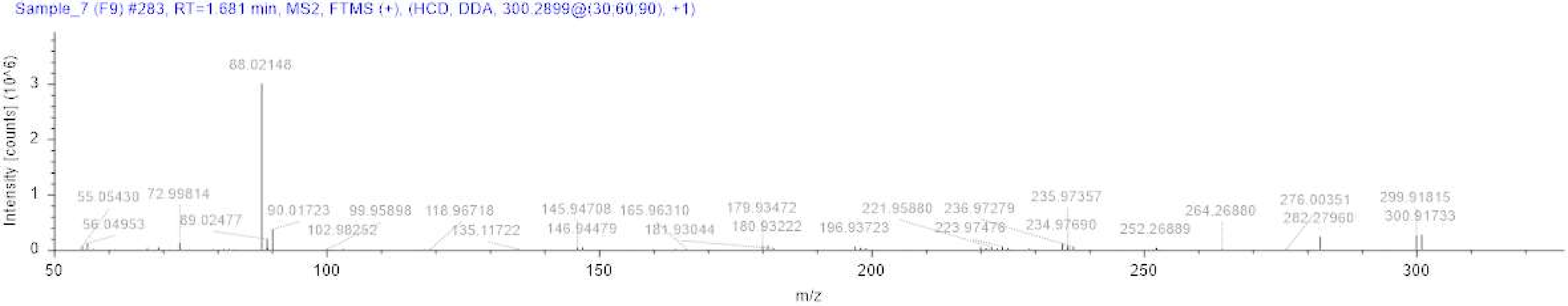

Compounds

17-Dec-2025 1:28

File name: 02_Cytb signature generation-(1)

Study: 02_Cytb signature generation

Structure	Name	RT [min]	Formula	Calc. MW	Group Areas
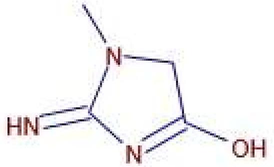	Creatinine	2.87	C4 H7 N3 O	113.05873	1.85e7 / 8.68e6

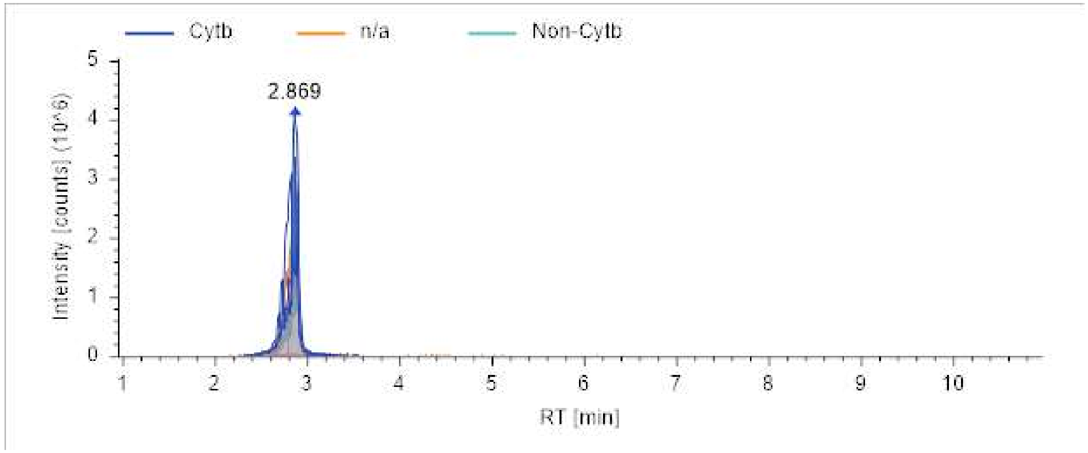

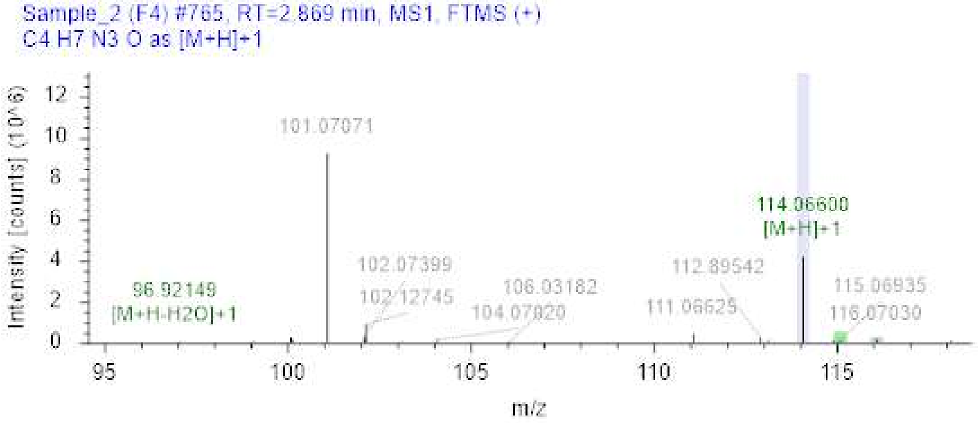

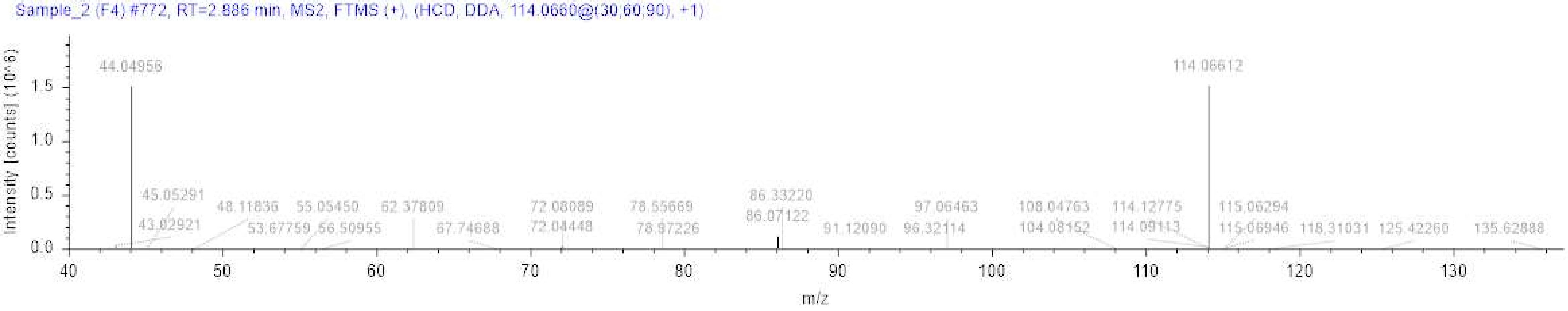

Compounds

17-Dec-2025 1:28

File name: 02_Cytb signature generation-(1)

Study: 02_Cytb signature generation

Structure	Name	RT [min]	Formula	Calc. MW	Group Areas
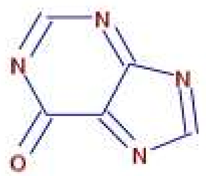	6-OXOPURINE	2.97	C5 H2 N4 O	136.03854	7.93e6 / 1.19e6

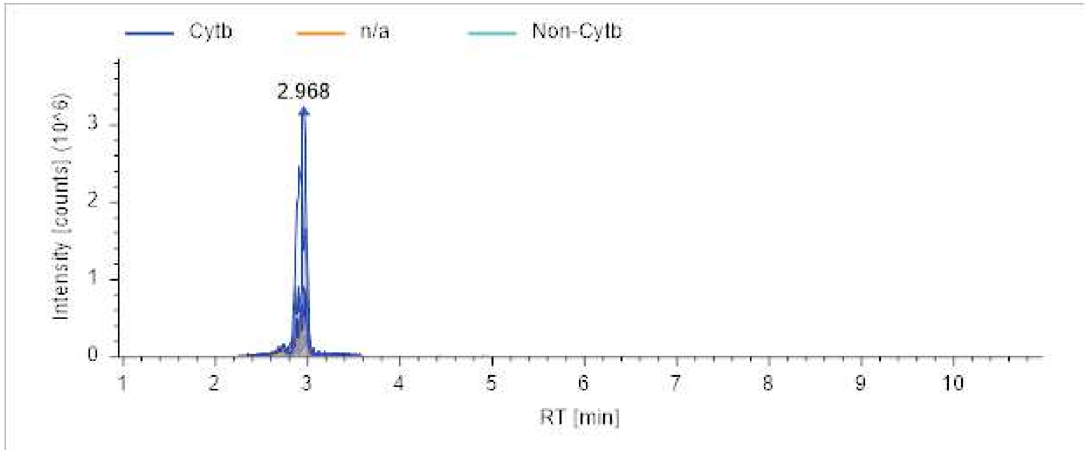

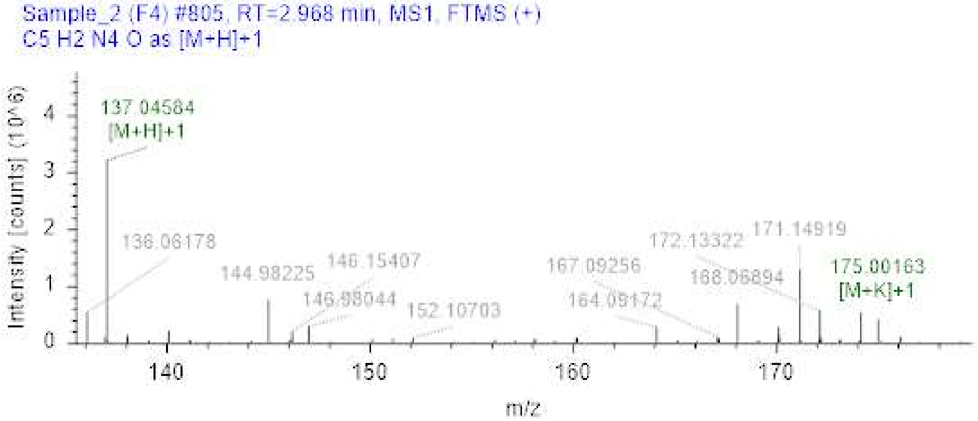

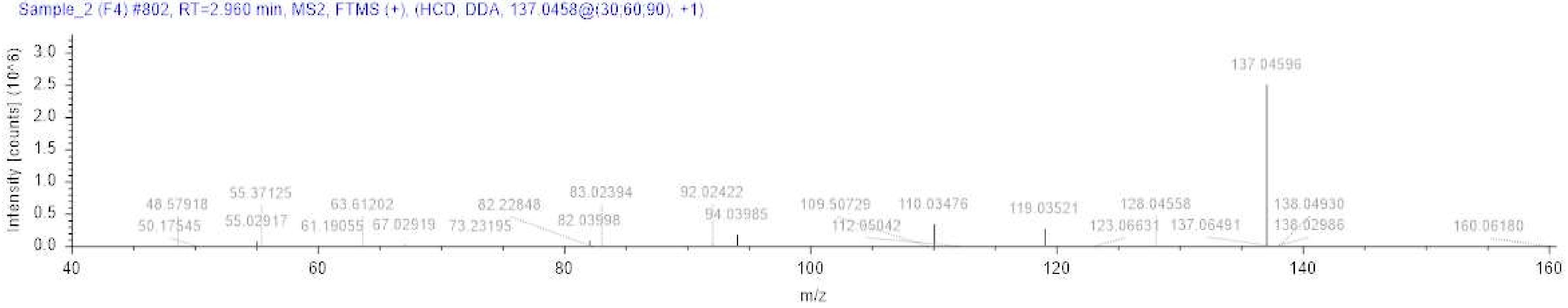

Compounds

17-Dec-2025 1:28

File name: 02_Cytb signature generation-(1)

Study: 02_Cytb signature generation

Structure	Name	RT [min]	Formula	Calc. MW	Group Areas
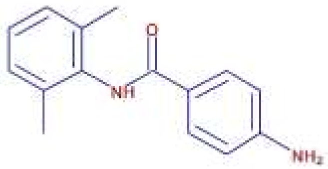	ameltolide	3.06	C15 H16 N2 O	240.12626	1.04e7 / 4.99e6

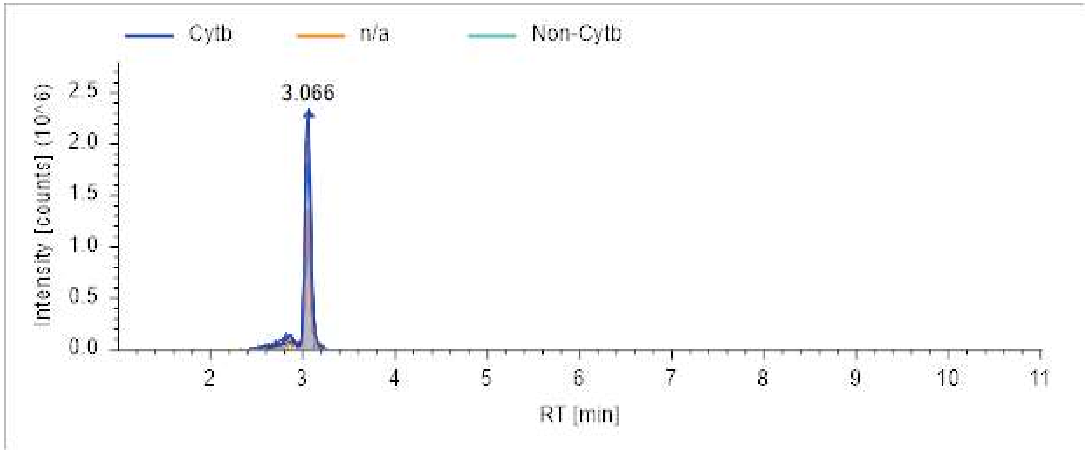

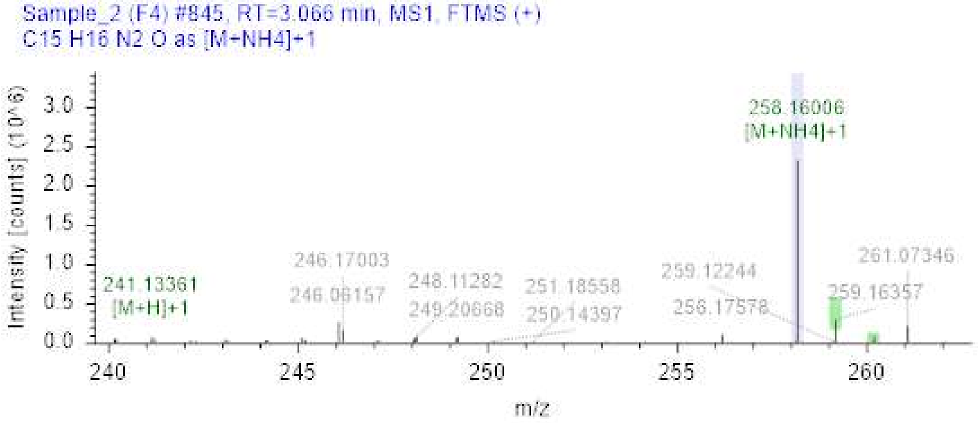

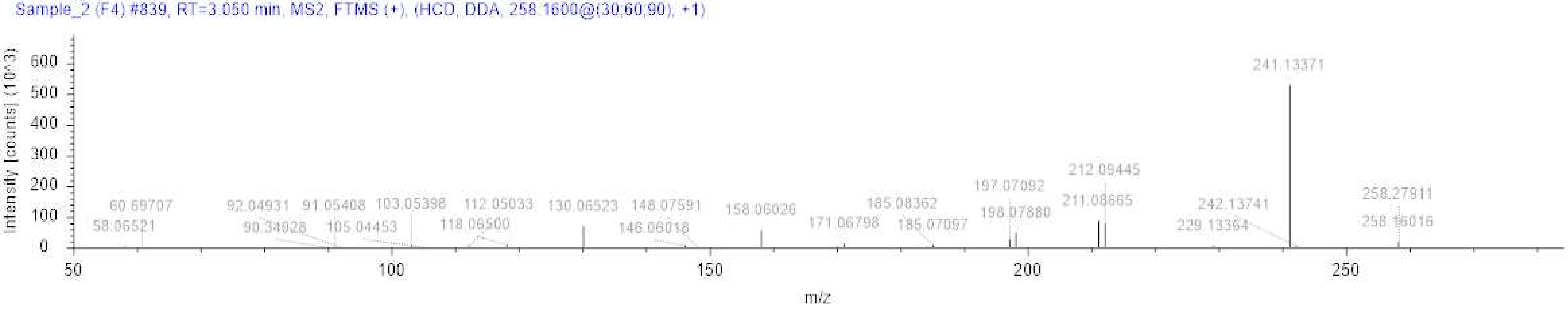

Compounds

17-Dec-2025 1:28

File name: 02_Cytb signature generation-(1)

Study: 02_Cytb signature generation

Structure	Name	RT [min]	Formula	Calc. MW	Group Areas
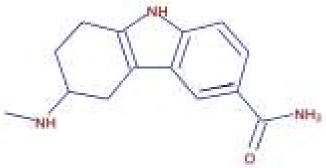	Frovatriptan	3.17	C14 H17 N3 O	243.13711	2.66e6 / 1.14e6

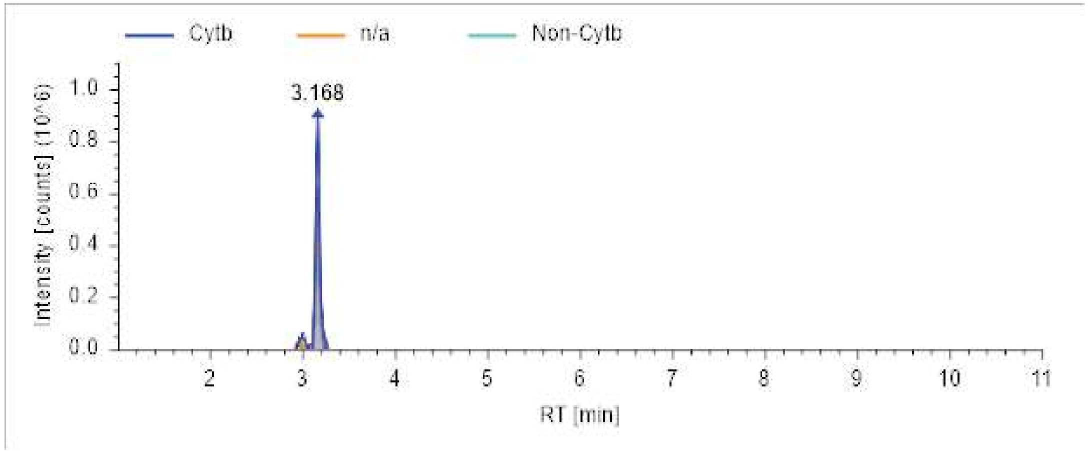

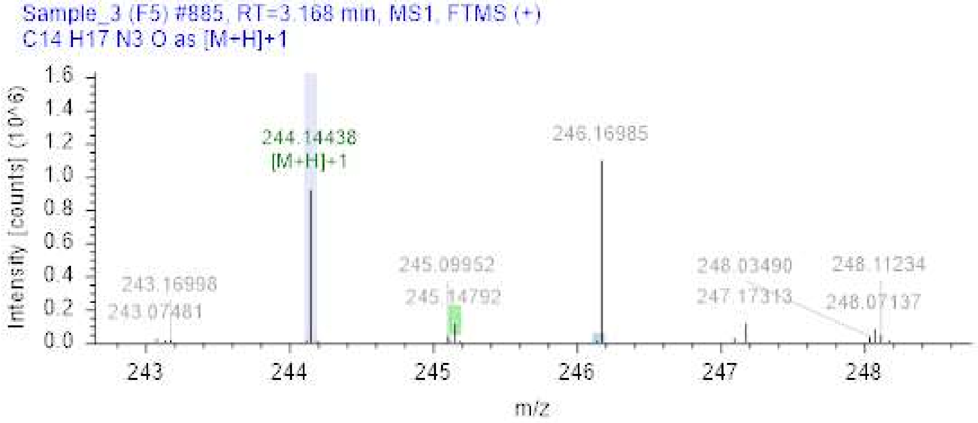

Compounds

17-Dec-2025 1:28

File name: 02_Cytb signature generation-(1)

Study: 02_Cytb signature generation

Structure	Name	RT [min]	Formula	Calc. MW	Group Areas
		3.26	C14 H27 N O4	273.19405	3.69e6 / 2.09e5

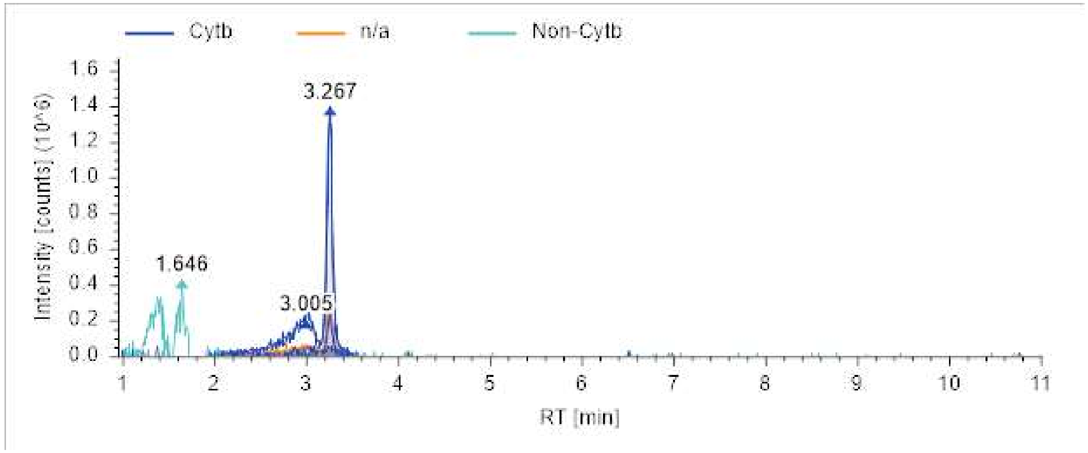

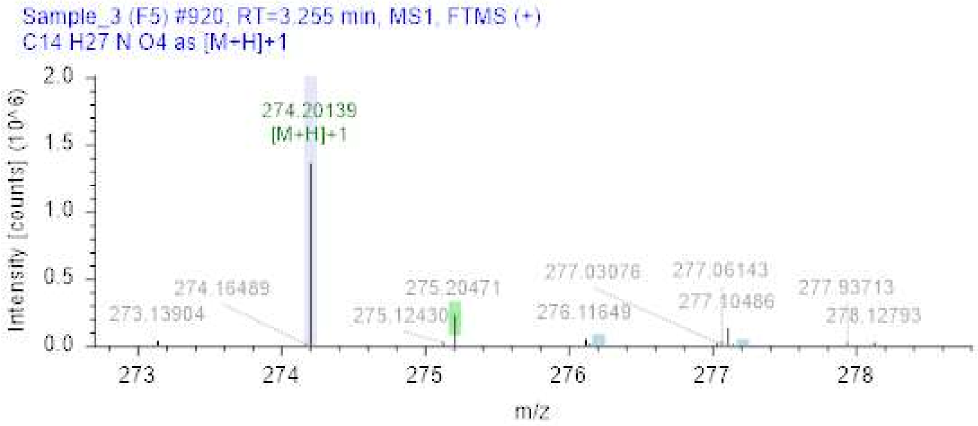

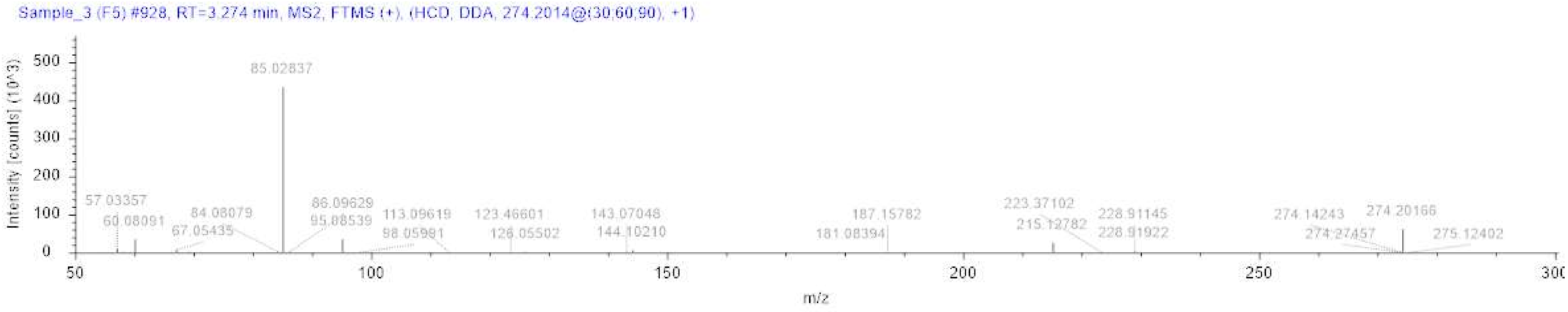

Compounds

17-Dec-2025 1:28

File name: 02_Cytb signature generation-(1)

Study: 02_Cytb signature generation

Structure	Name	RT [min]	Formula	Calc. MW	Group Areas	
		3.29	C14 H17 N3 O S	275.10923	6.24e5	1.67e5

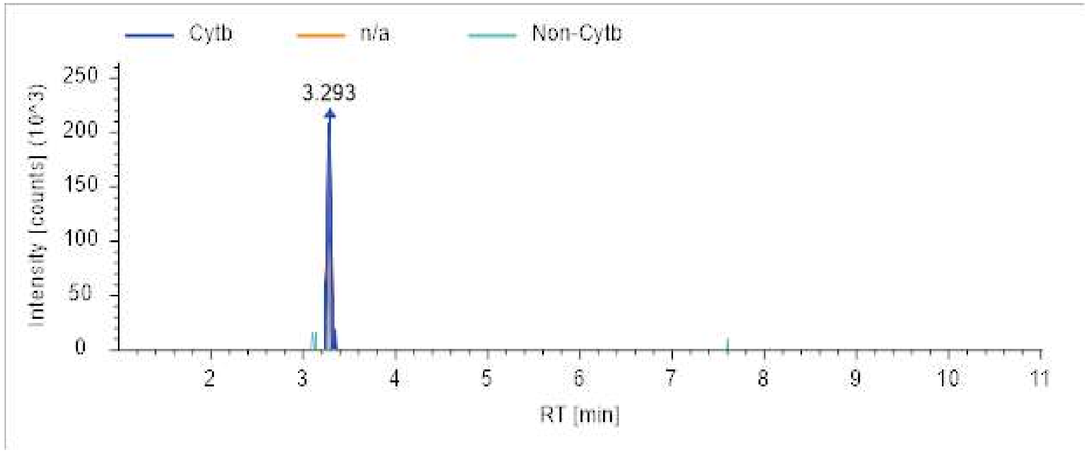

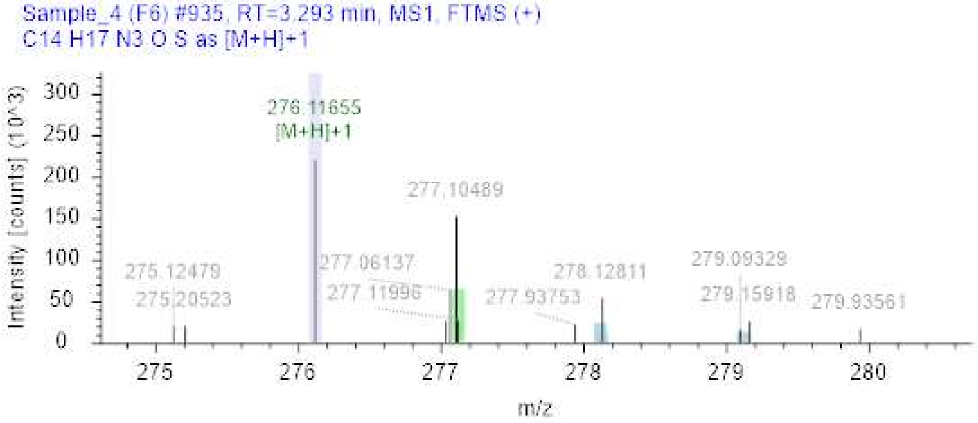

Compounds

17-Dec-2025 1:28

File name: 02_Cytb signature generation-(1)

Study: 02_Cytb signature generation

Structure	Name	RT [min]	Formula	Calc. MW	Group Areas
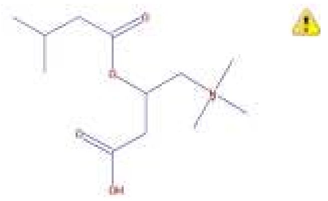	Isovalerylcarnitine	3.65	C12 H23 N O4	245.16271	9.93e8 / 1.35e8

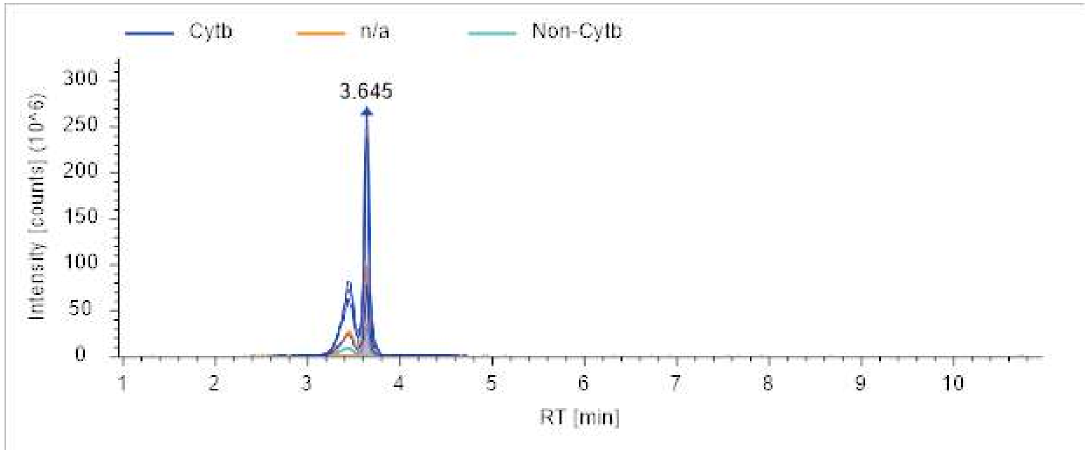

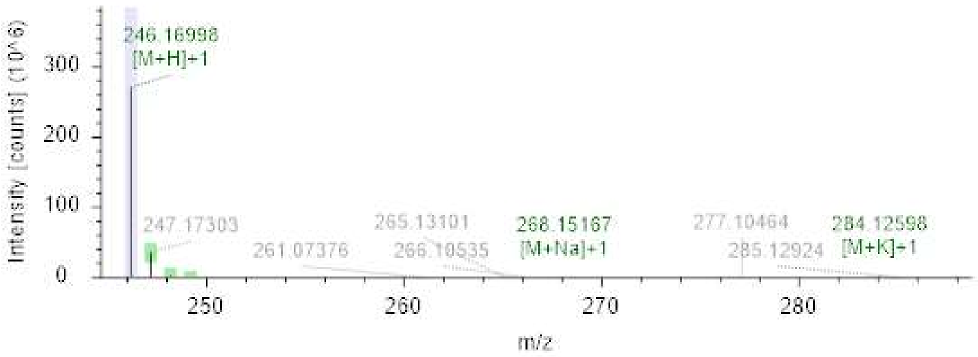

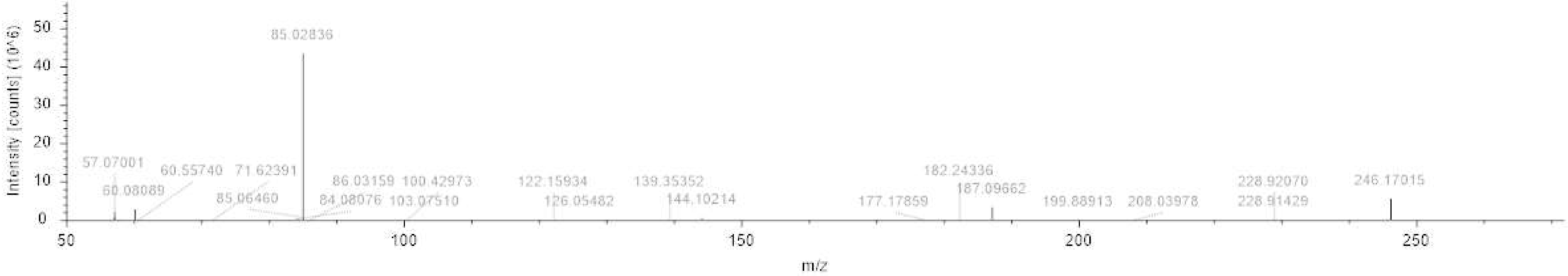

File name: 02_Cytb signature generation-(1)

Study: 02_Cytb signature generation

Structure	Name	RT [min]	Formula	Calc. MW	Group Areas
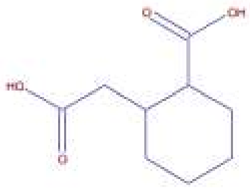	cis-2-Carboxycyclohexyl-acetic acid	3.65	C9 H14 O4	186.08899	1.37e6 1.22e5

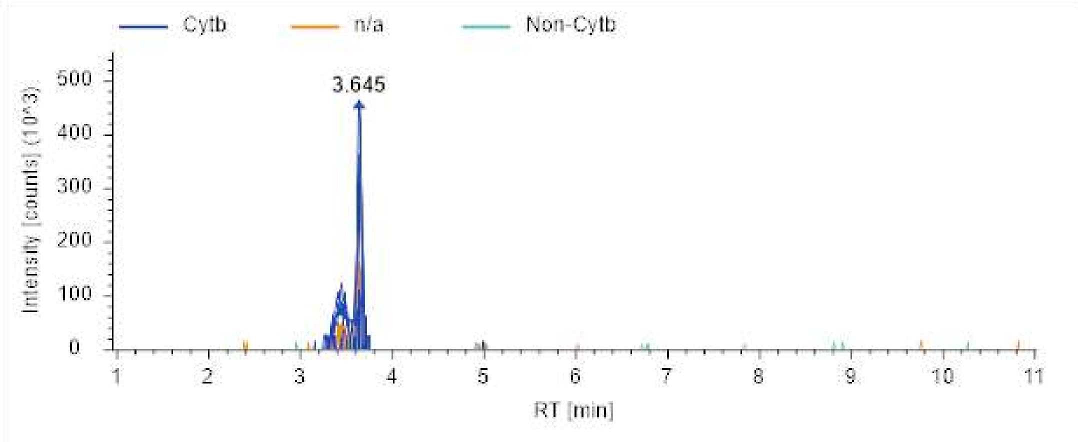

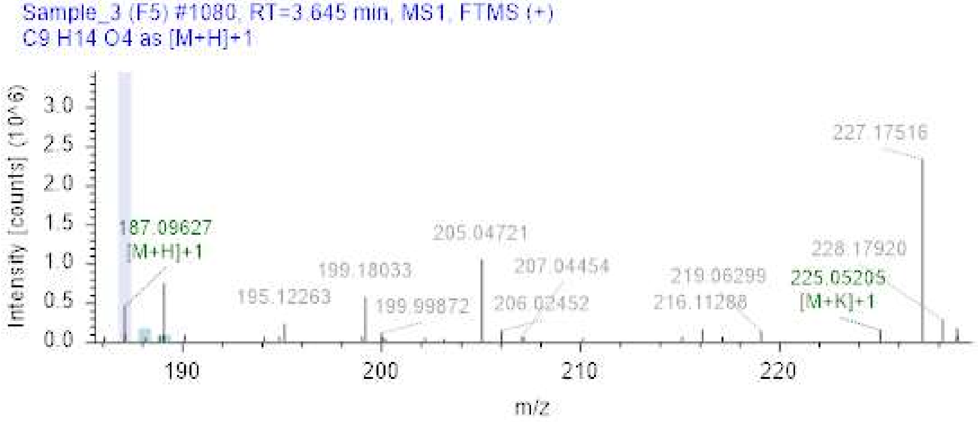

Compounds

17-Dec-2025 1:28

File name: 02_Cytb signature generation-(1)

Study: 02_Cytb signature generation

Structure	Name	RT [min]	Formula	Calc. MW	Group Areas
		4.16	C7 H10 N O4 P	203.03475	1.80e6 7.80e5

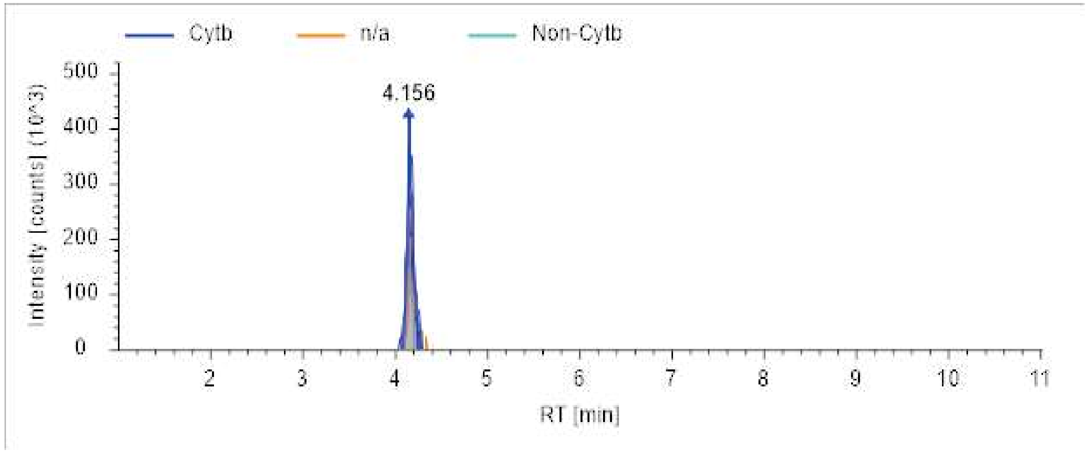

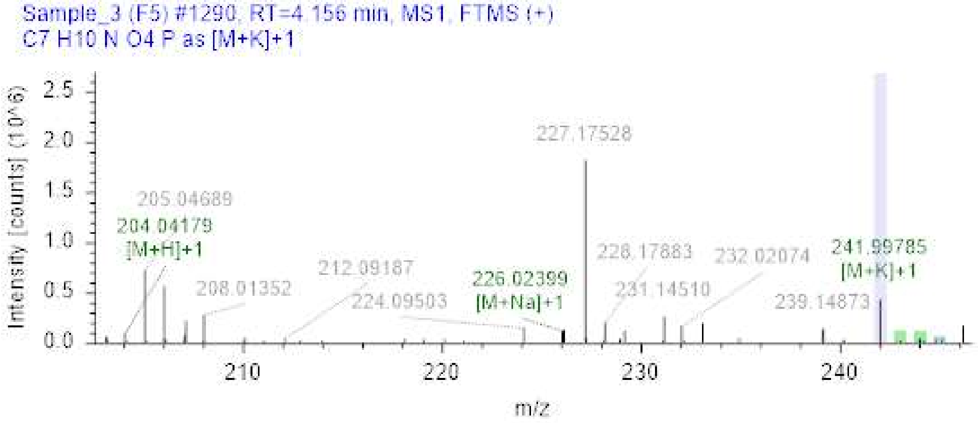

Compounds

17-Dec-2025 1:28

File name: 02_Cytb signature generation-(1)

Study: 02_Cytb signature generation

Structure	Name	RT [min]	Formula	Calc. MW	Group Areas
		4.17	C8 H9 N	119.07338	3.48e7 / 1.31e7

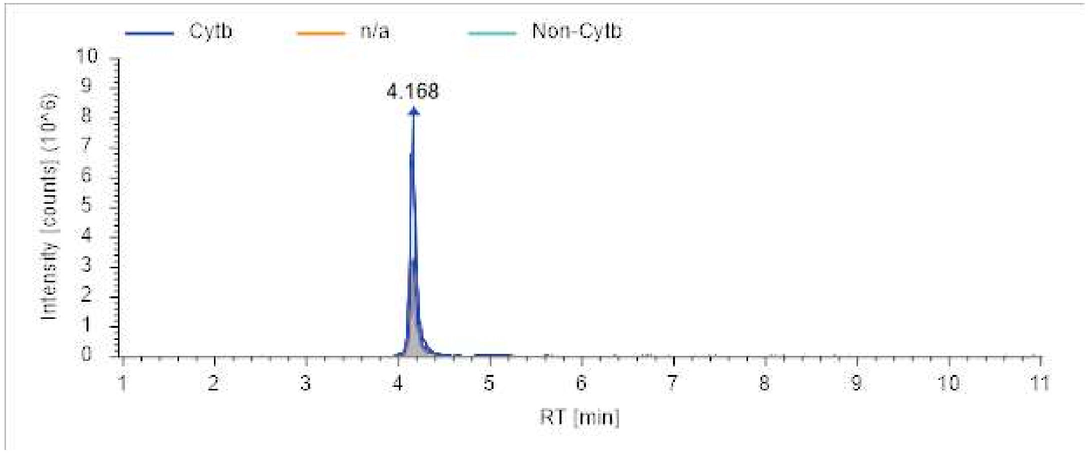

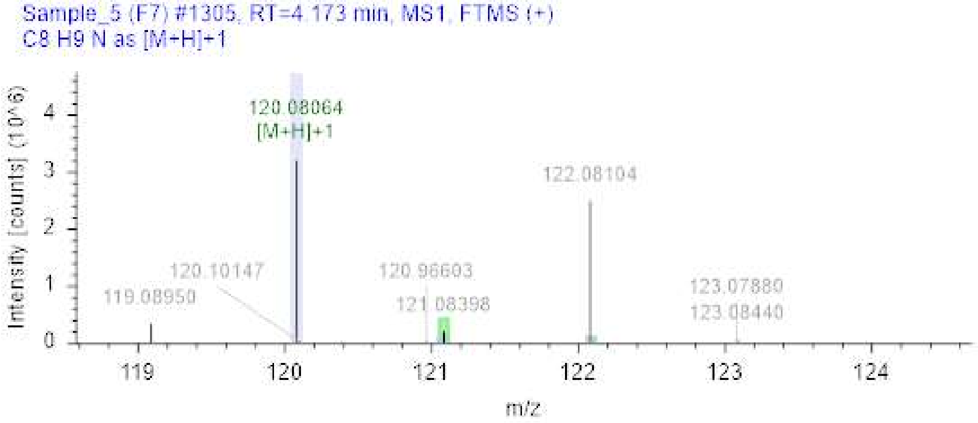

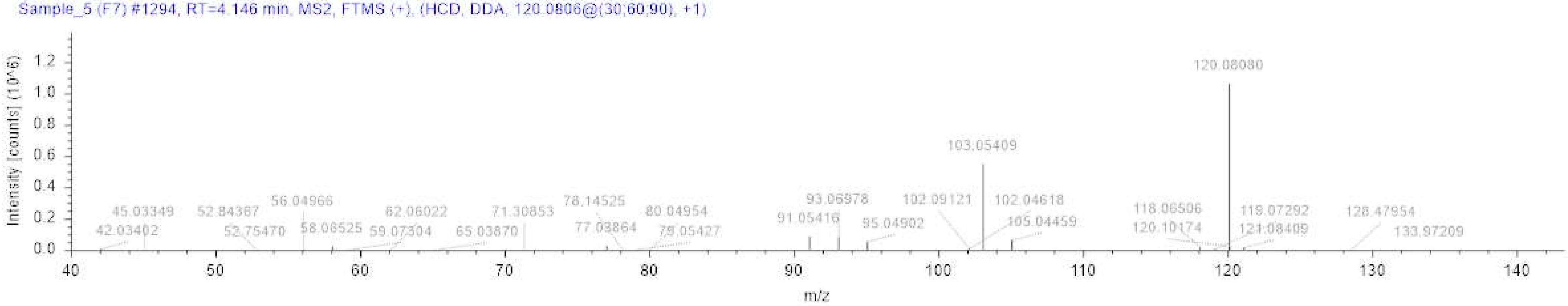

Compounds

17-Dec-2025 1:28

File name: 02_Cytb signature generation-(1)

Study: 02_Cytb signature generation

Structure	Name	RT [min]	Formula	Calc. MW	Group Areas
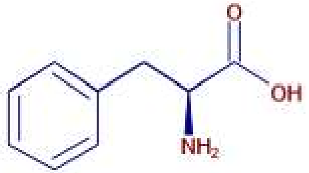	Phenylalanine	4.17	C9 H11 N O2	148.05250	3.12e8 / 1.22e8

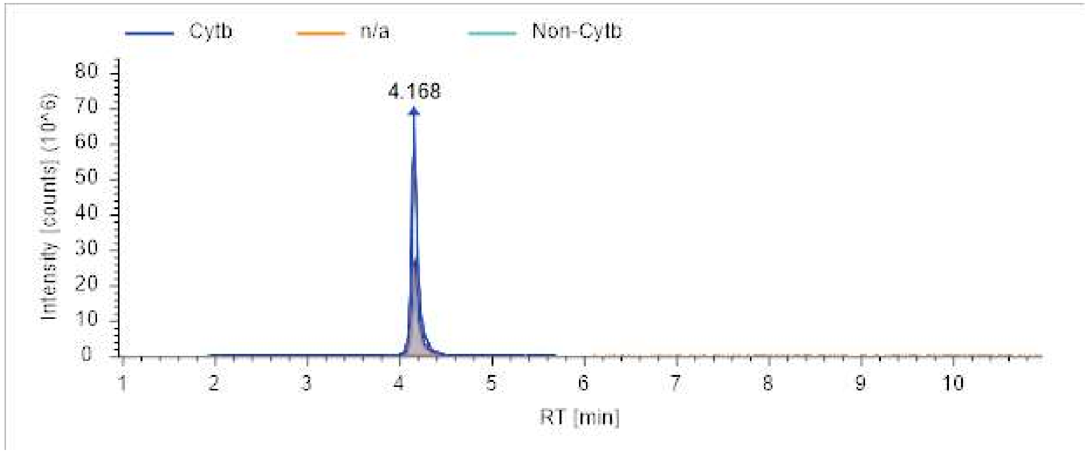

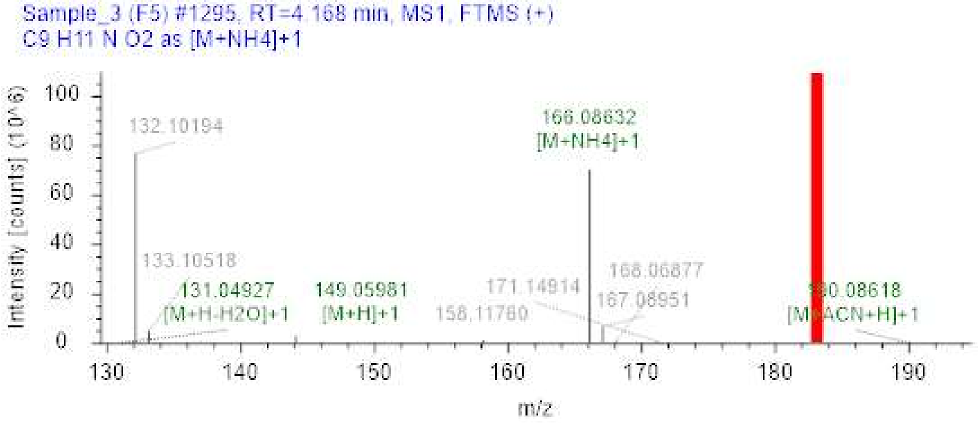

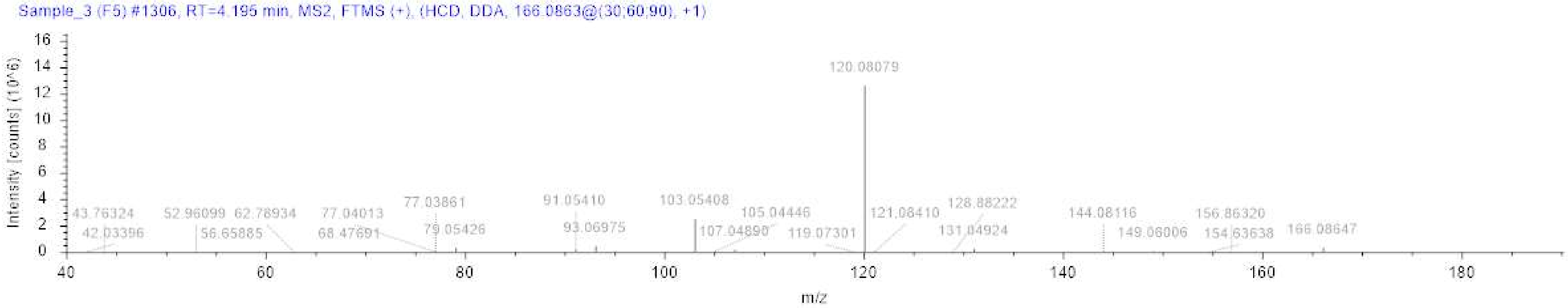

File name: 02_Cytb signature generation-(1)

Study: 02_Cytb signature generation

Structure	Name	RT [min]	Formula	Calc. MW	Group Areas
	[Similar to: Phenylethanolamine; ΔMass: -35.0372 Da]	4.17	C8 H6	102.04683	3.48e7 1.31e7

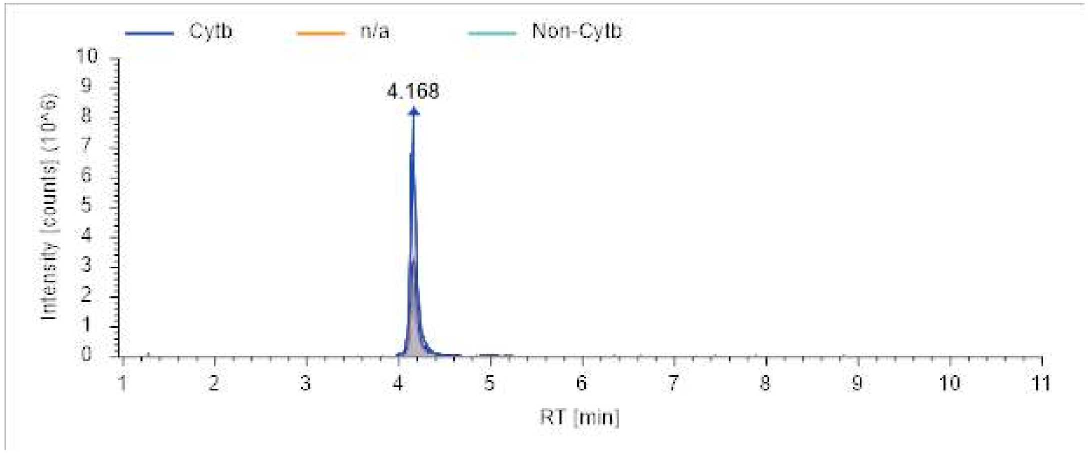

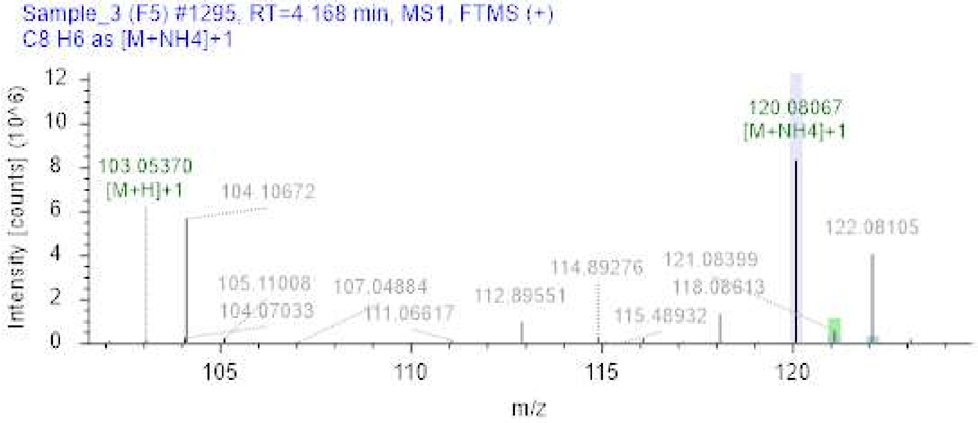

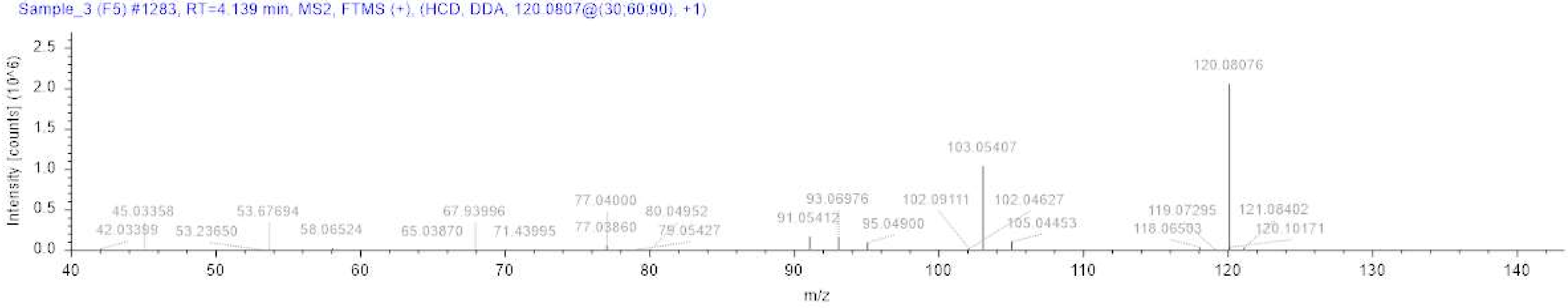

Compounds

17-Dec-2025 1:28

File name: 02_Cytb signature generation-(1)

Study: 02_Cytb signature generation

Structure	Name	RT [min]	Formula	Calc. MW	Group Areas
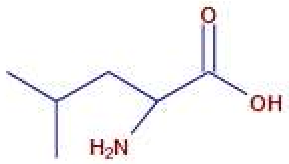	Leucine	4.19	C6 H13 N O2	131.09467	4.23e8 2.02e8

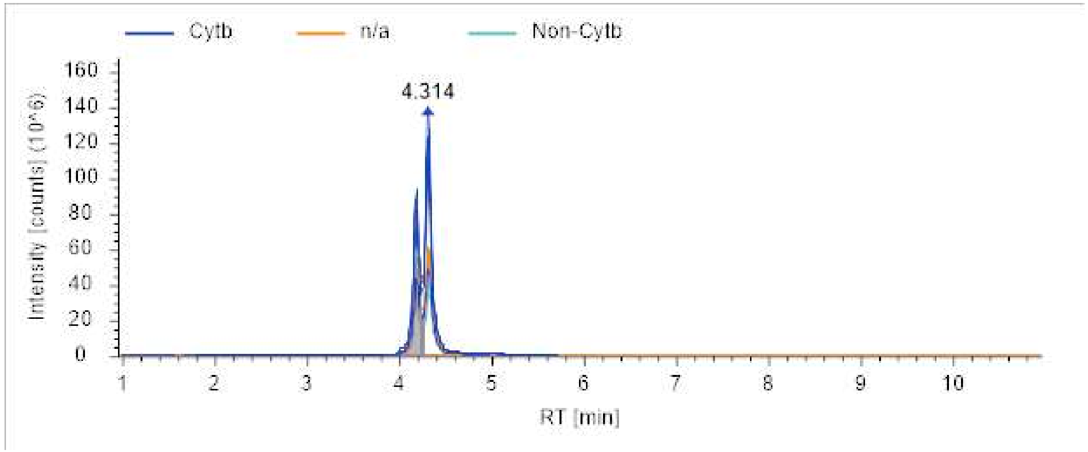

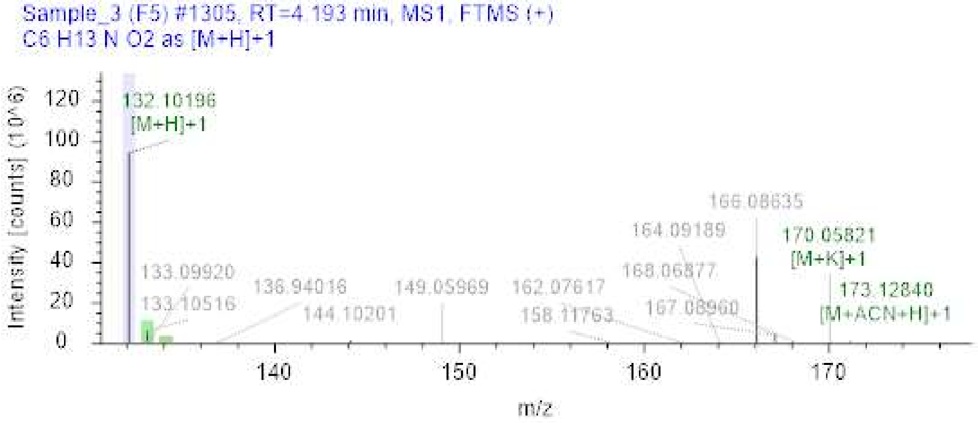

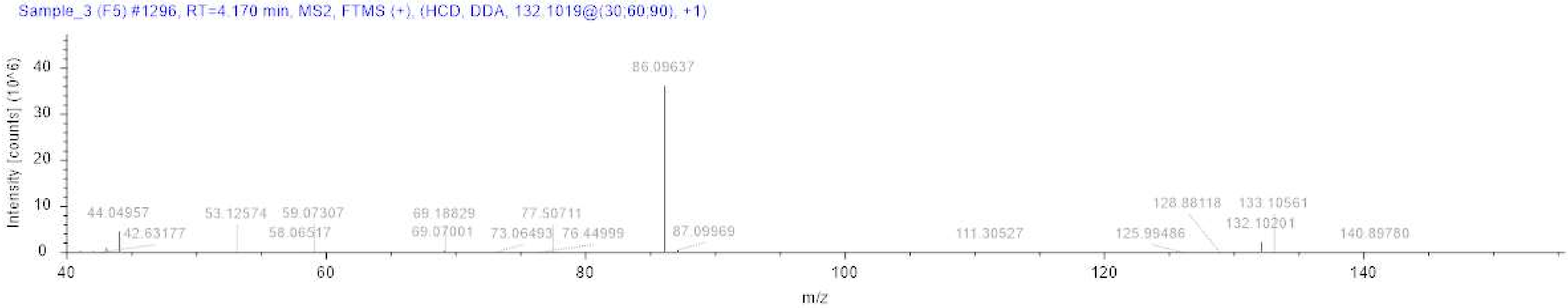

Compounds

17-Dec-2025 1:28

File name: 02_Cytb signature generation-(1)

Study: 02_Cytb signature generation

Structure	Name	RT [min]	Formula	Calc. MW	Group Areas
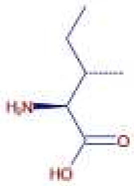	Isoleucine	4.32	C6 H13 N O2	131.09468	6.69e8 / 2.72e8

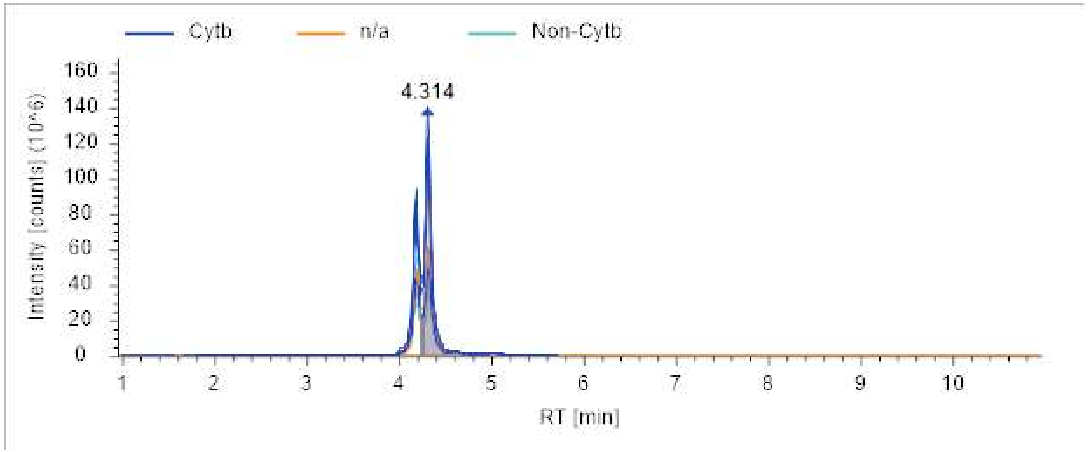

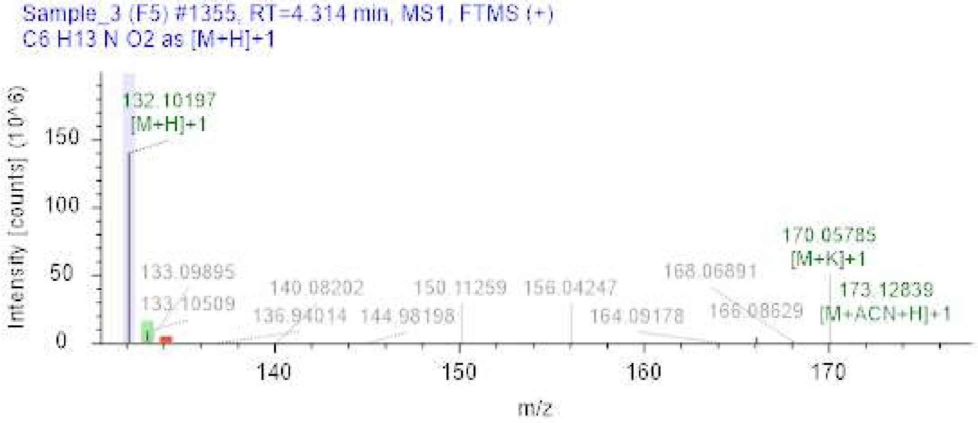

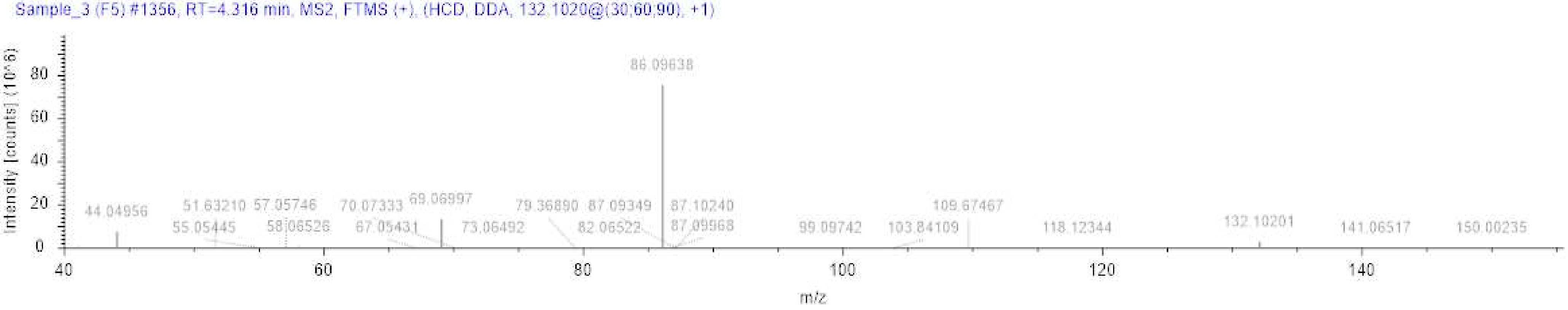

Compounds

17-Dec-2025 1:28

File name: 02_Cytb signature generation-(1)

Study: 02_Cytb signature generation

Structure	Name	RT [min]	Formula	Calc. MW	Group Areas
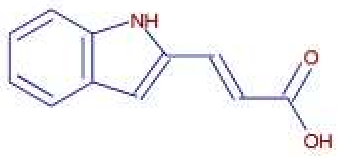	trans-3-Indoleacrylic acid	4.51	C11 H9 N O2	187.06314	2.98e7 / 1.06e7

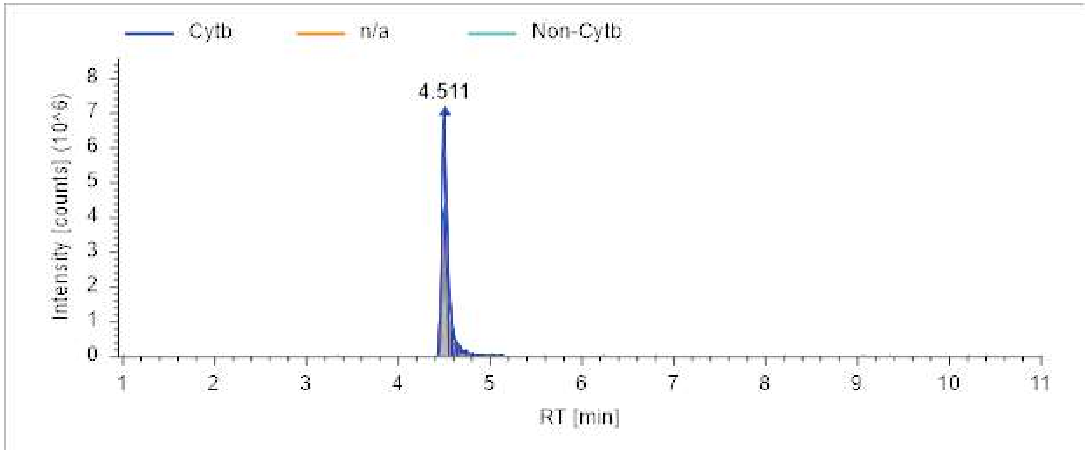

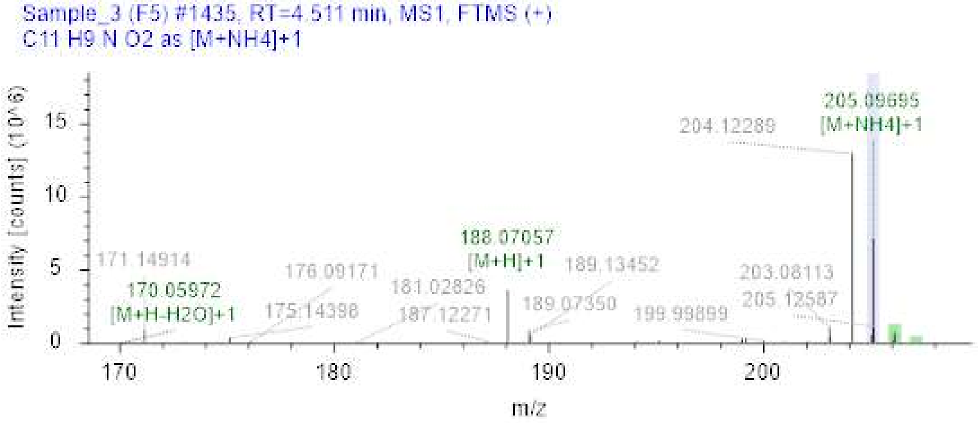

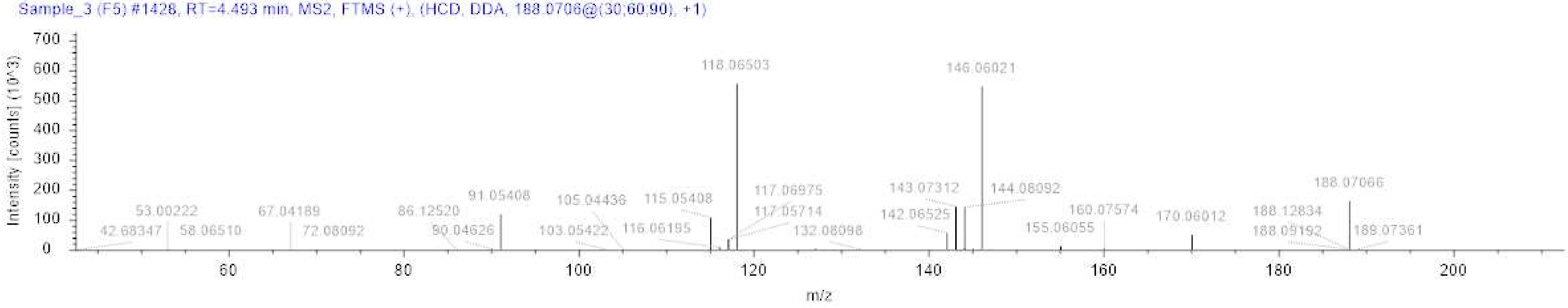

Compounds

17-Dec-2025 1:28

File name: 02_Cytb signature generation-(1)

Study: 02_Cytb signature generation

Structure	Name	RT [min]	Formula	Calc. MW	Group Areas
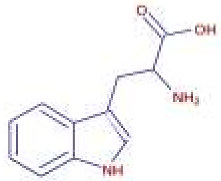	Tryptophan	4.51	C11 H12 N2 O2	204.08957	2.98e7 1.06e7

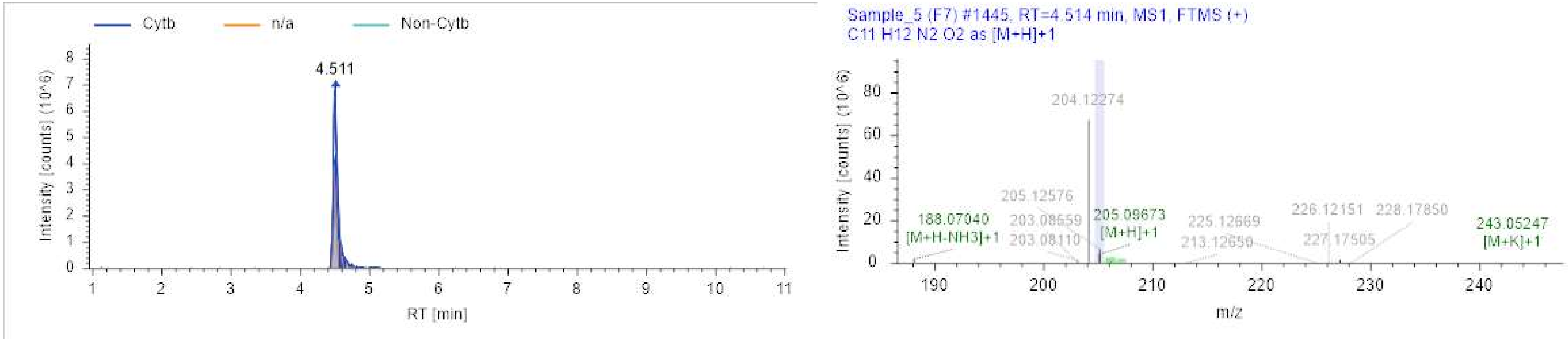

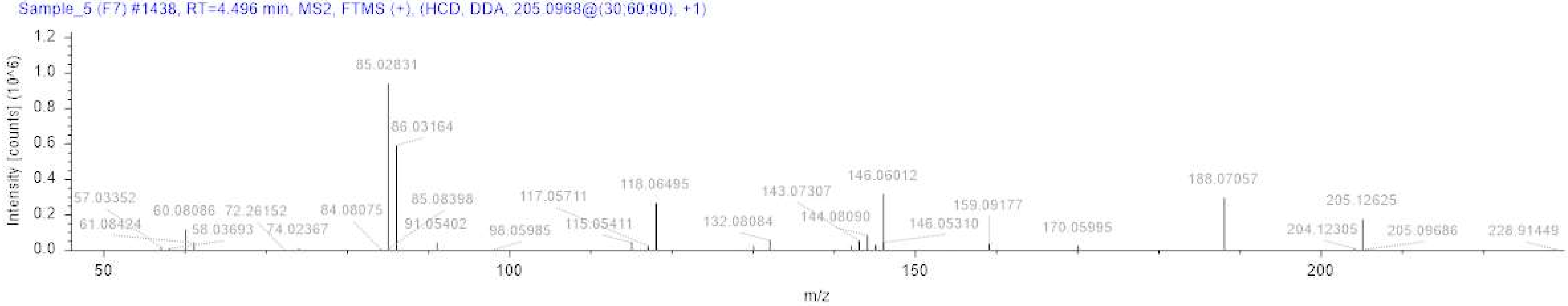

Compounds

17-Dec-2025 1:28

File name: 02_Cytb signature generation-(1)

Study: 02_Cytb signature generation

Structure	Name	RT [min]	Formula	Calc. MW	Group Areas	
		4.51	C16 H5 Cl N2 P2 S	353.93407	7.27e5	3.07e5

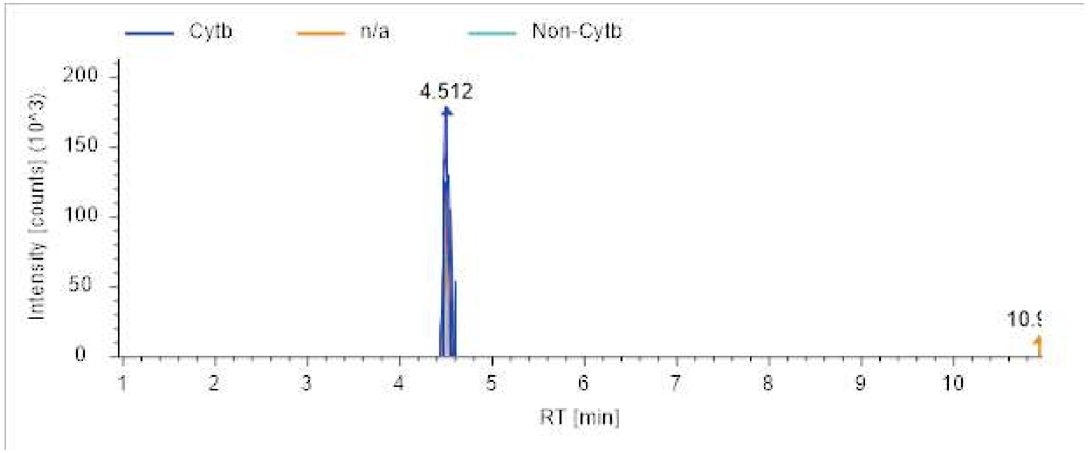

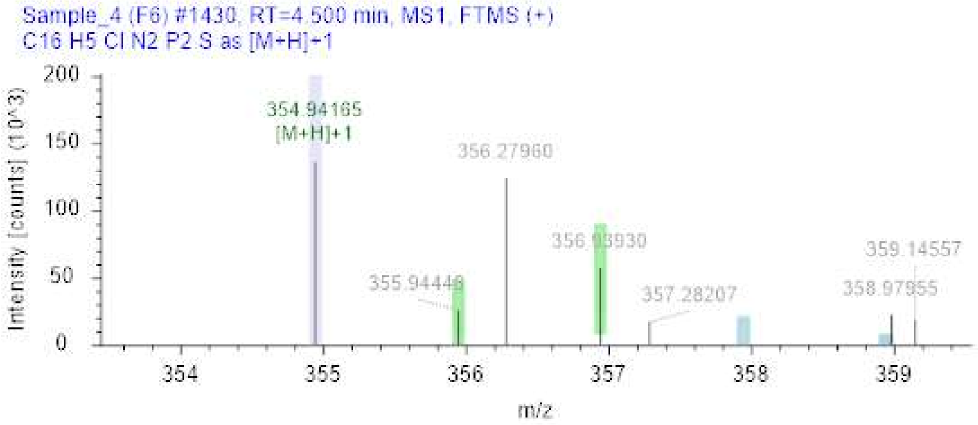

Compounds

17-Dec-2025 1:28

File name: 02_Cytb signature generation-(1)

Study: 02_Cytb signature generation

Structure	Name	RT [min]	Formula	Calc. MW	Group Areas
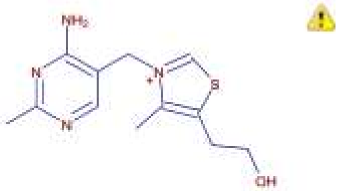	Thiamine	4.55	C12 H16 N4 O S	264.10417	8.59e6 / 3.28e6

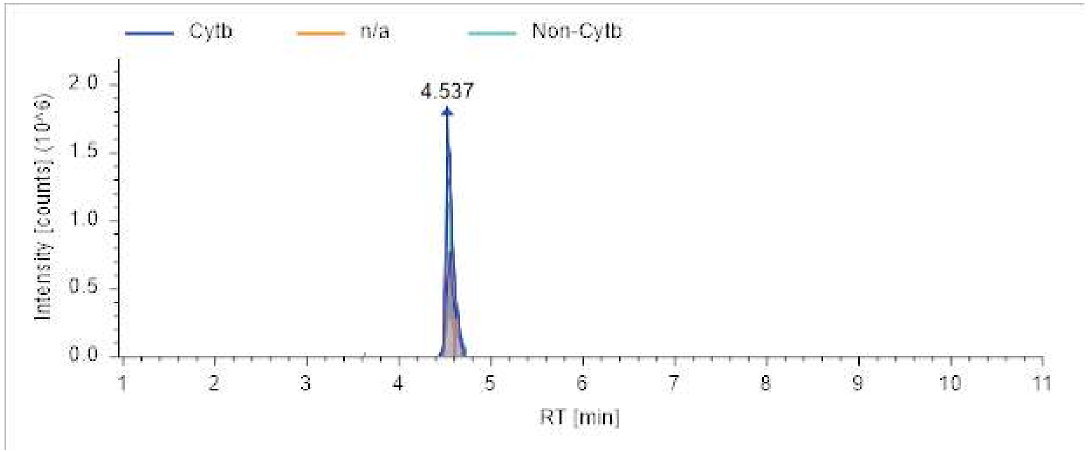

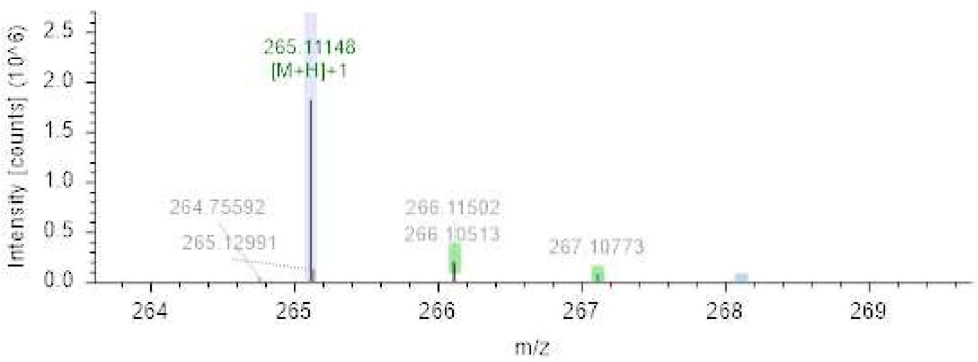

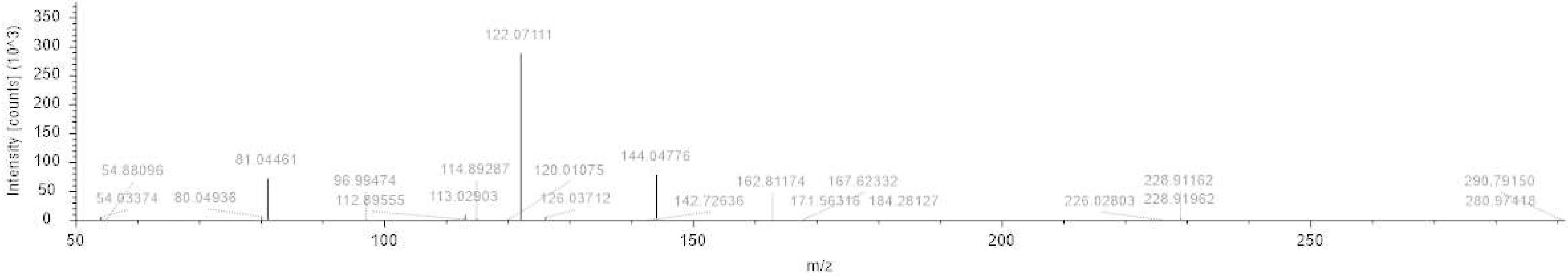

Compounds

17-Dec-2025 1:28

File name: 02_Cytb signature generation-(1)

Study: 02_Cytb signature generation

Structure	Name	RT [min]	Formula	Calc. MW	Group Areas
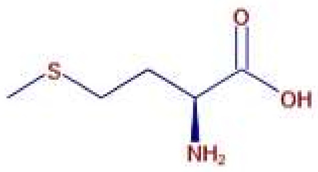	Methionine	4.57	C5 H11 N O2 S	132.02436	4.51e7 / 1.25e7

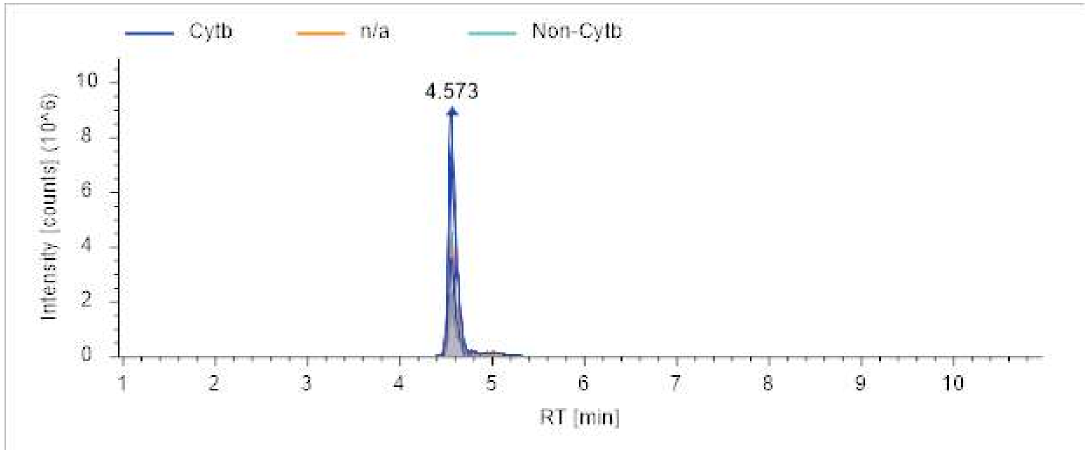

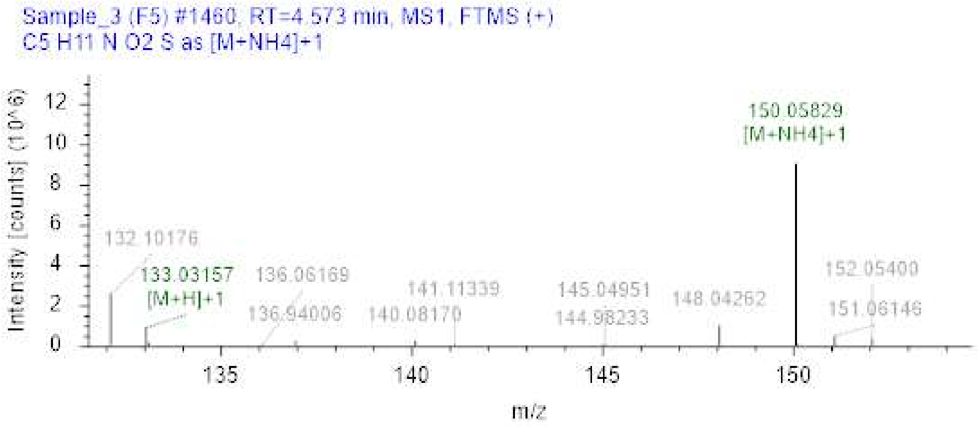

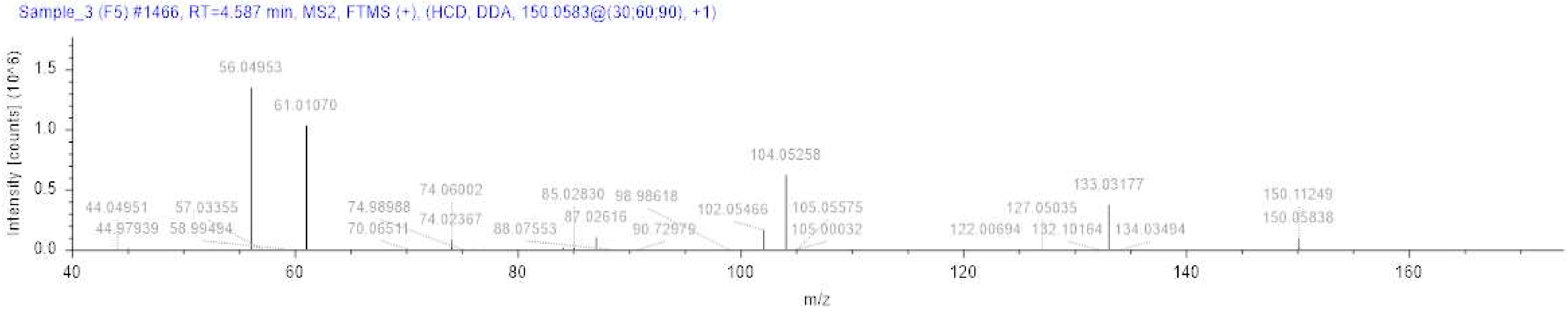

Structure	Name	RT [min]	Formula	Calc. MW	Group Areas
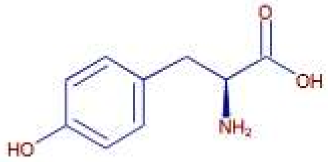	Tyrosine	5.05	C9 H11 N O3	164.04735	1.82e7 / 6.48e6

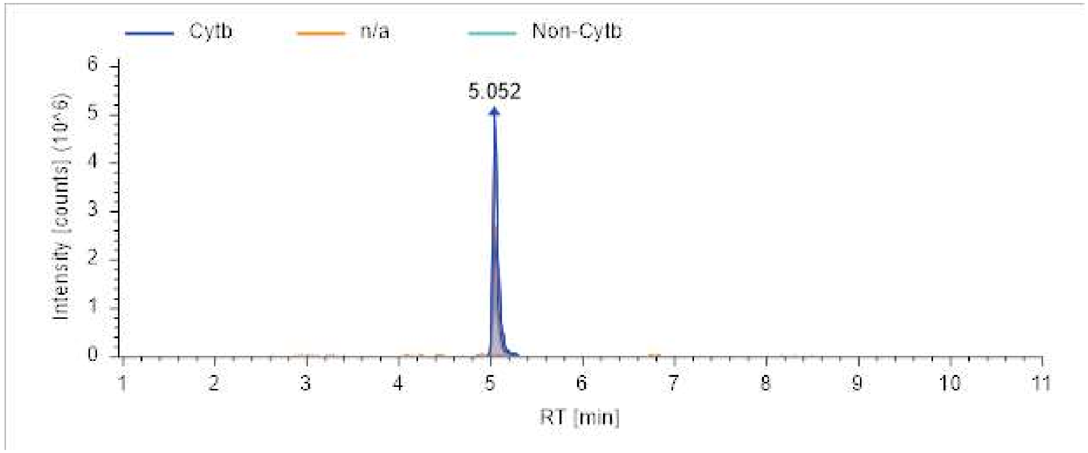

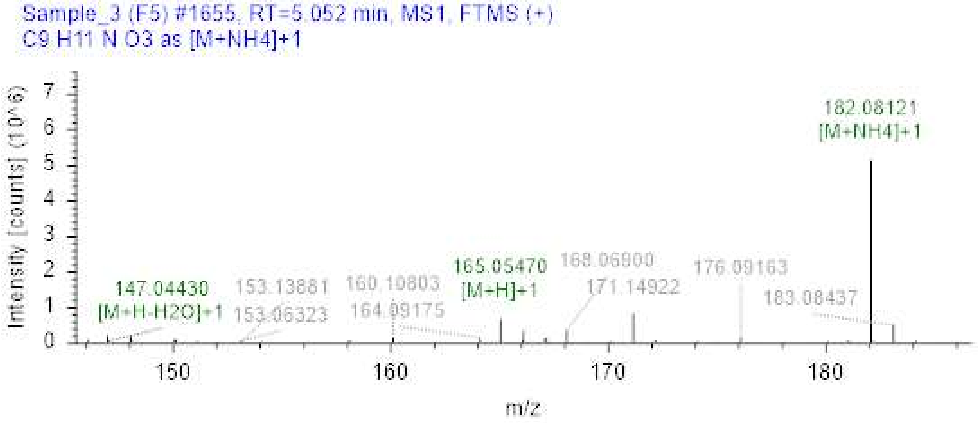

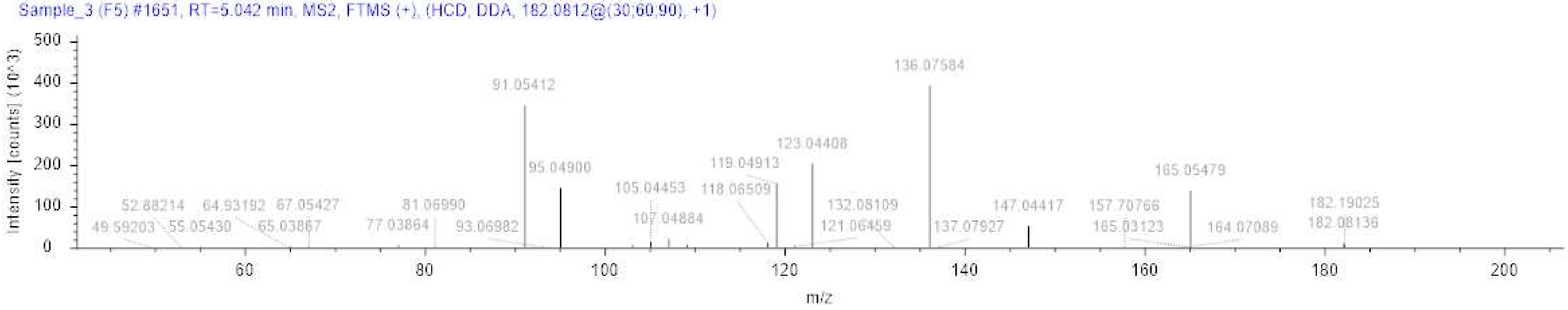

Compounds

17-Dec-2025 1:28

File name: 02_Cytb signature generation-(1)

Study: 02_Cytb signature generation

Structure	Name	RT [min]	Formula	Calc. MW	Group Areas
		5.46	C8 H Cl2 N4 O7 P	365.89577	1.99e6 7.93e5

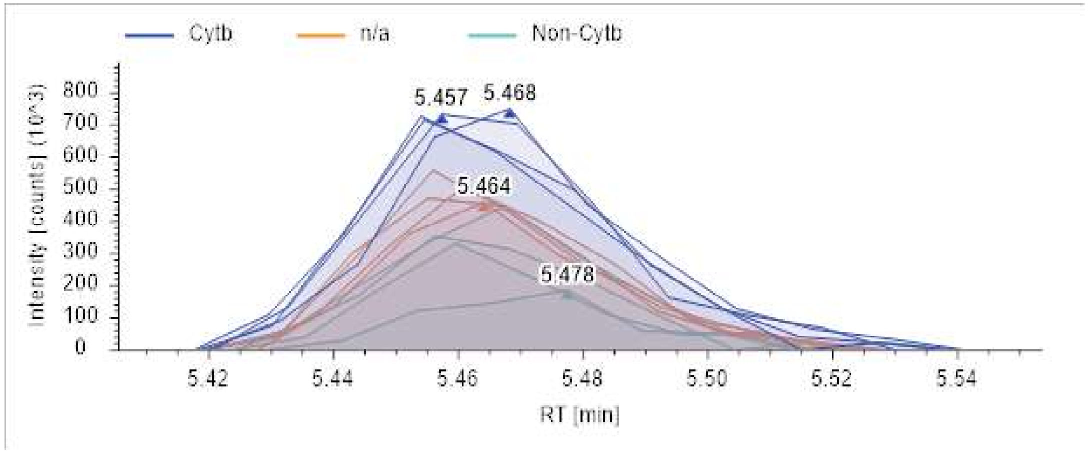

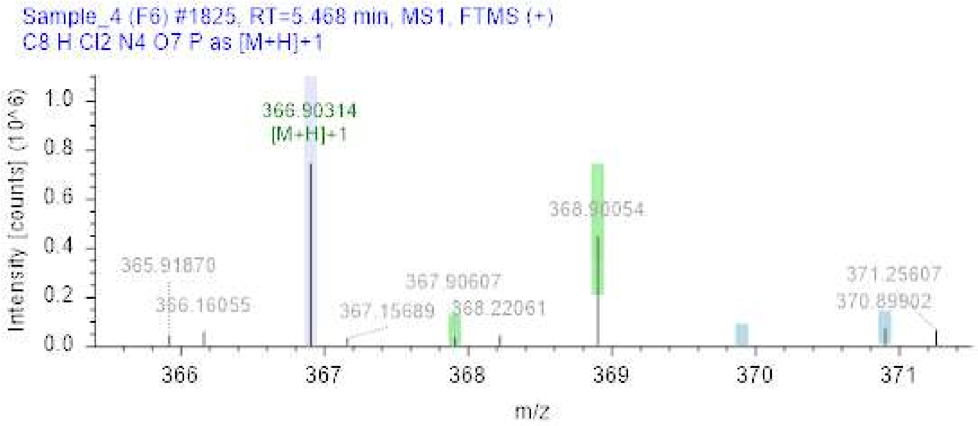

Compounds

17-Dec-2025 1:28

File name: 02_Cytb signature generation-(1)

Study: 02_Cytb signature generation

Structure	Name	RT [min]	Formula	Calc. MW	Group Areas
		5.46	C3 H4 Cl2 N2 O13 P2 S	439.82862	4.52e5 / 1.57e5

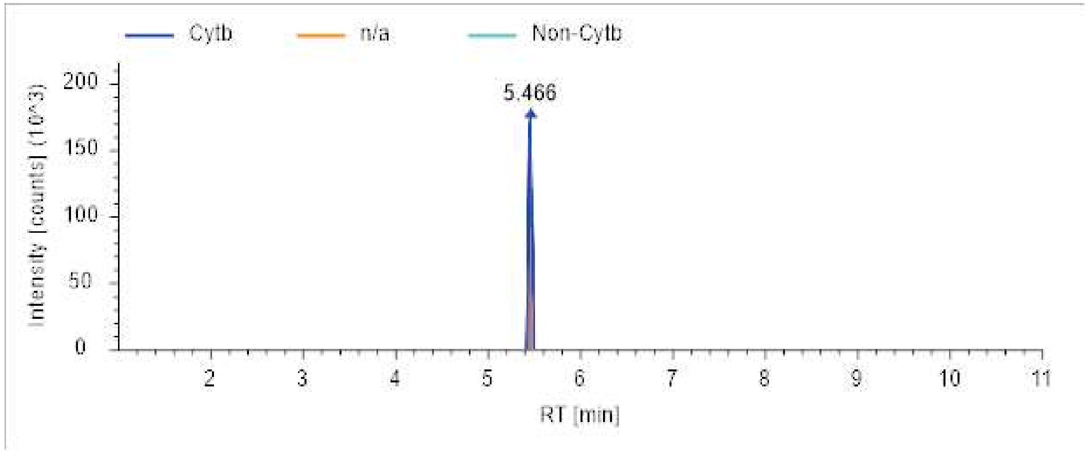

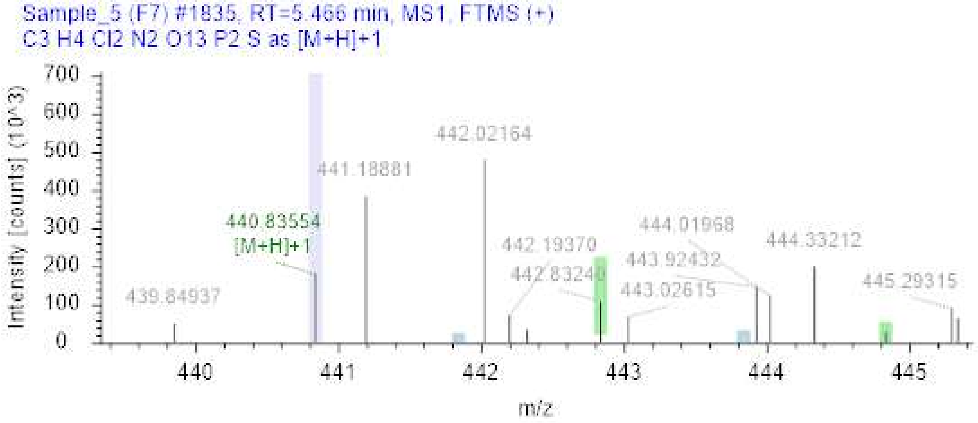

Compounds

17-Dec-2025 1:28

File name: 02_Cytb signature generation-(1)

Study: 02_Cytb signature generation

Structure	Name	RT [min]	Formula	Calc. MW	Group Areas	
		5.46	C11 H10 Cl N5 O13 P2	516.94436	2.37e6	1.01e6

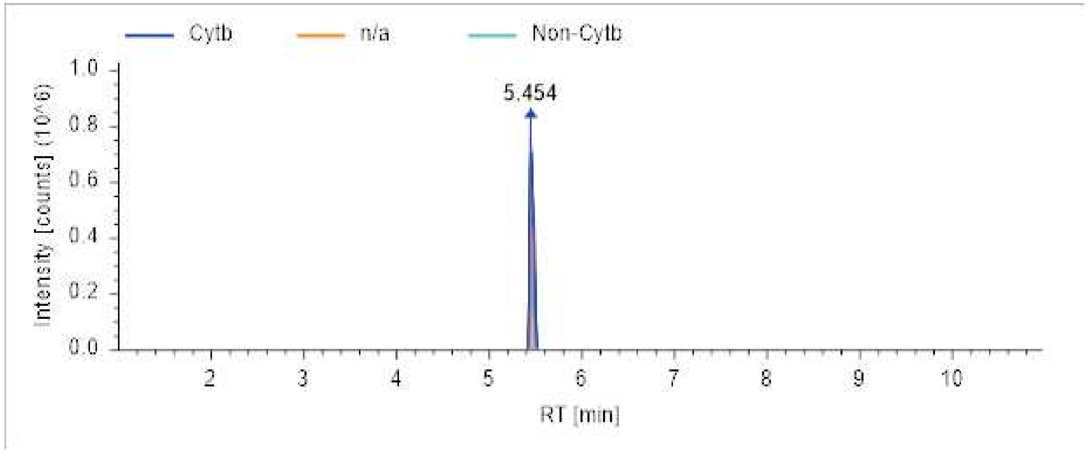

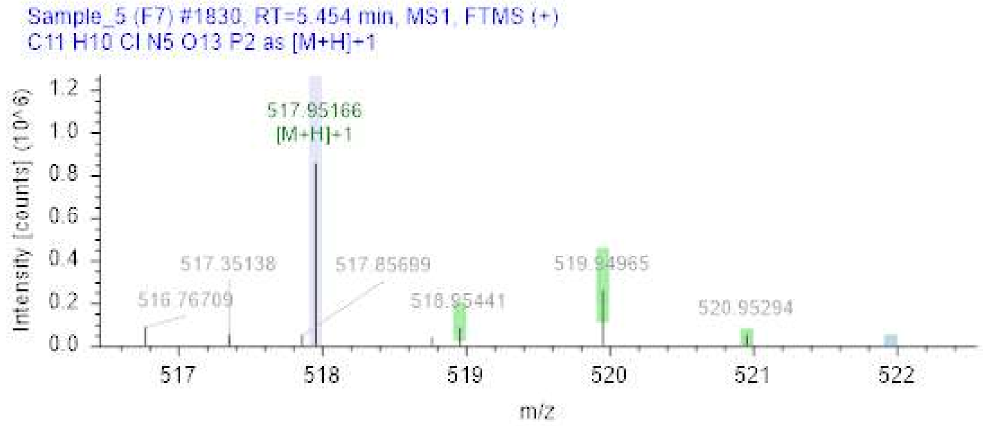

Compounds

17-Dec-2025 1:28

File name: 02_Cytb signature generation-(1)

Study: 02_Cytb signature generation

Structure	Name	RT [min]	Formula	Calc. MW	Group Areas	
		5.46	C6 H2 N2 O12	293.96112	8.12e6	2.91e6

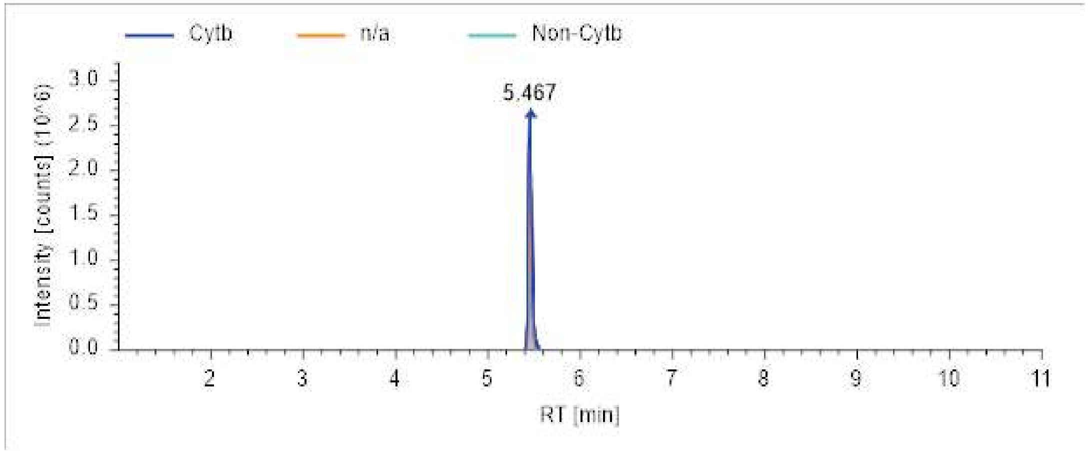

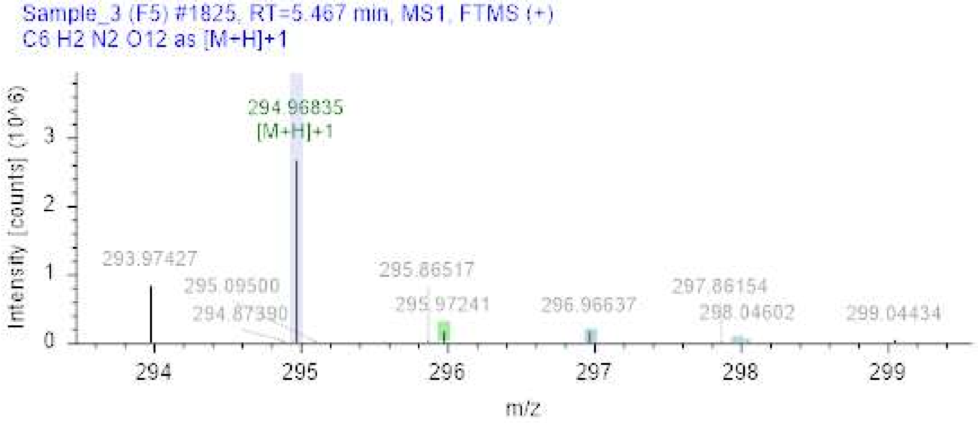

Structure	Name	RT [min]	Formula	Calc. MW	Group Areas
		5.46	C4 H10 Br Cl N7 O12 P	492.89950	4.77e5 / 5.79e4

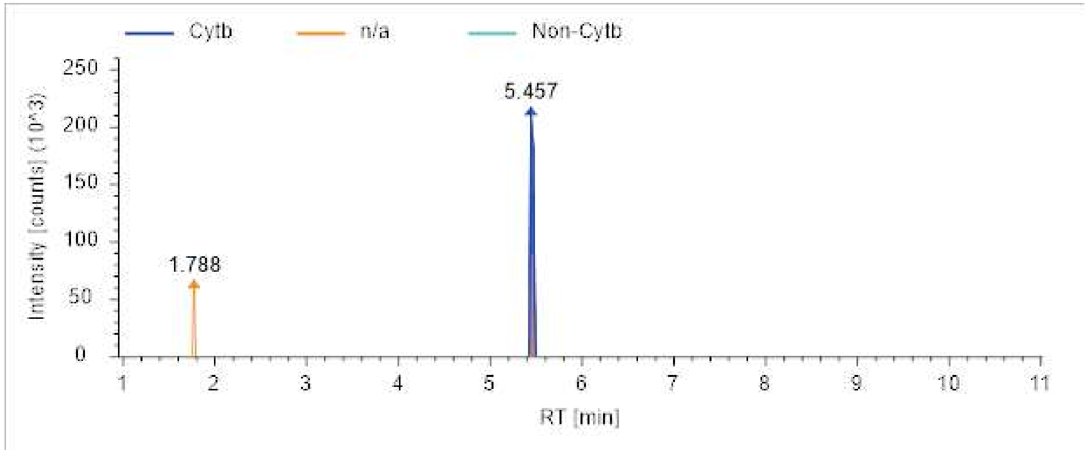

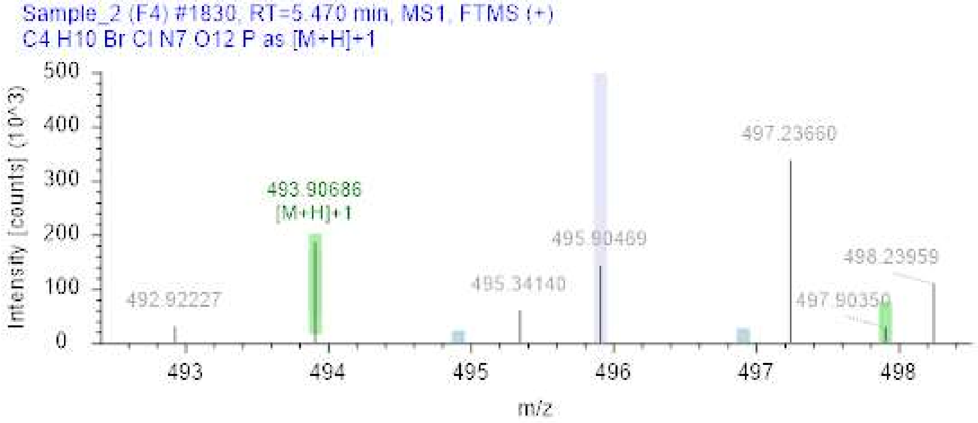

Compounds

17-Dec-2025 1:28

File name: 02_Cytb signature generation-(1)

Study: 02_Cytb signature generation

Structure	Name	RT [min]	Formula	Calc. MW	Group Areas
		5.47	C10 H22 Cl2 O17 P2	545.97154	1.37e6 / 2.52e5

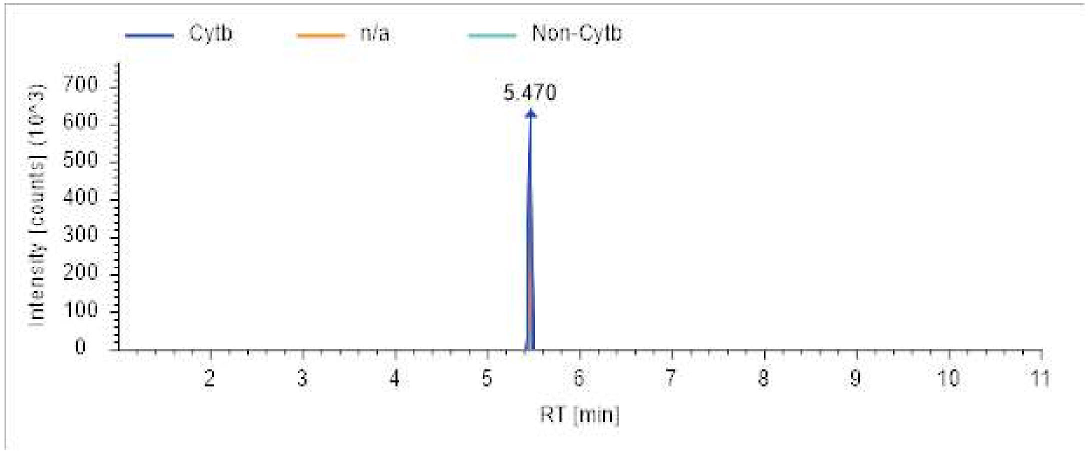

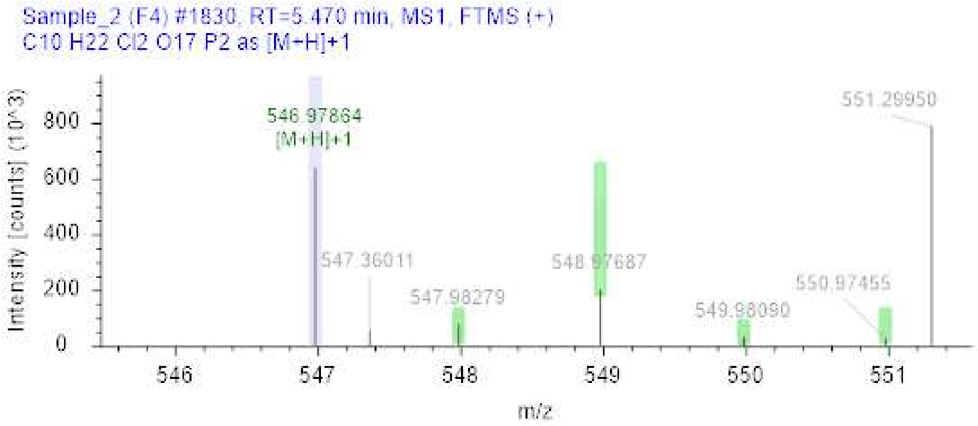

Compounds

17-Dec-2025 1:28

File name: 02_Cytb signature generation-(1)

Study: 02_Cytb signature generation

Structure	Name	RT [min]	Formula	Calc. MW	Group Areas
		5.47	C10 H11 Cl N O13 P	418.96696	7.94e6 / 1.92e6

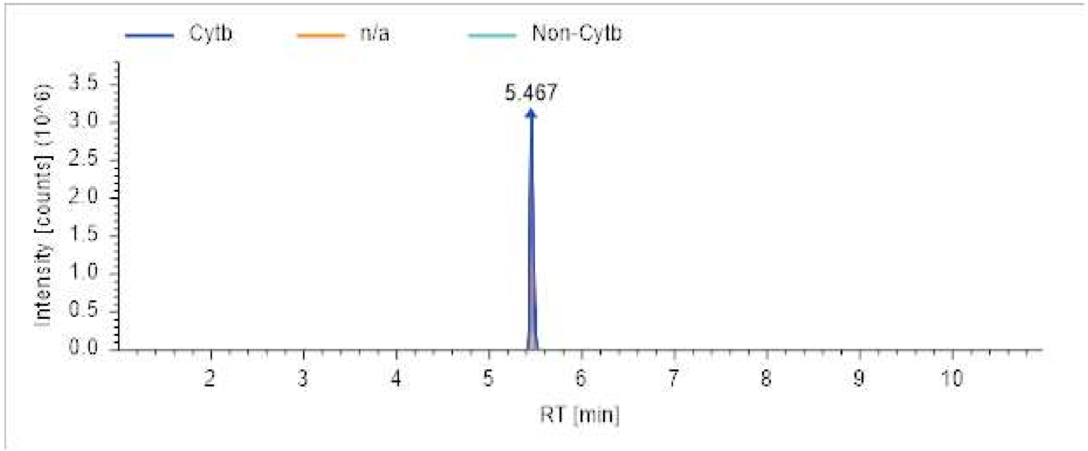

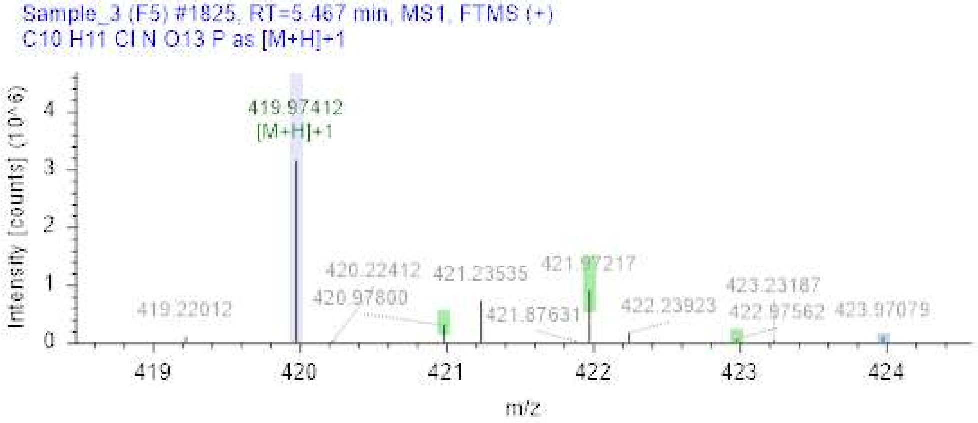

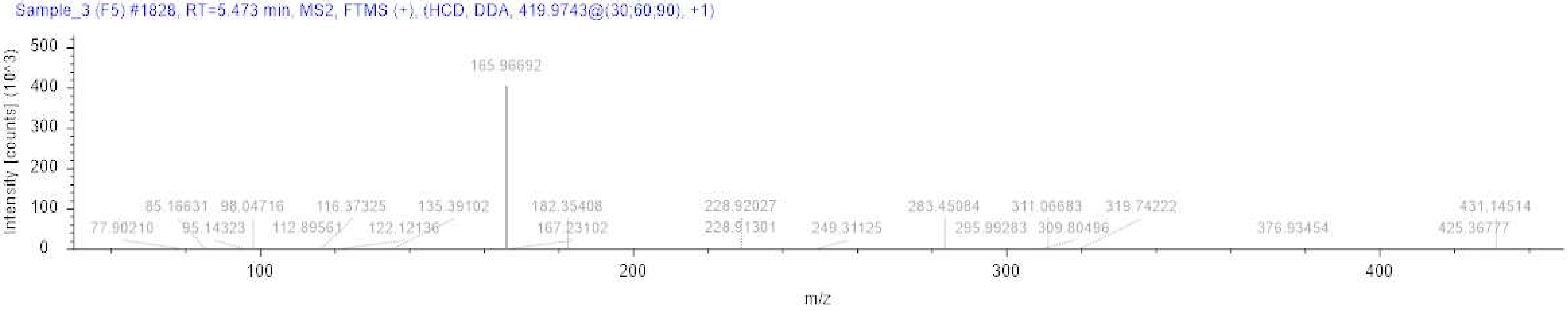

Compounds

17-Dec-2025 1:28

File name: 02_Cytb signature generation-(1)

Study: 02_Cytb signature generation

Structure	Name	RT [min]	Formula	Calc. MW	Group Areas
		5.47	C15 H16 Cl N2 O15 P	529.99698	6.65e5 8.23e4

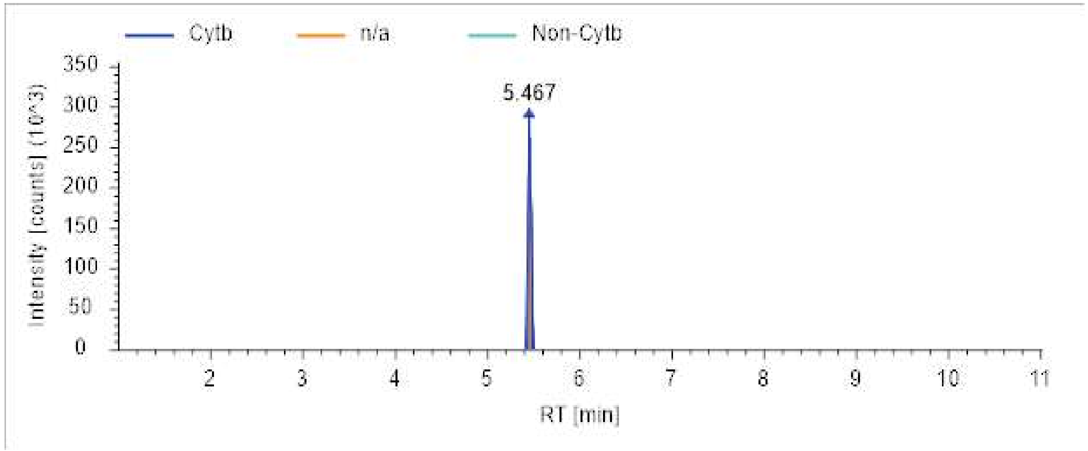

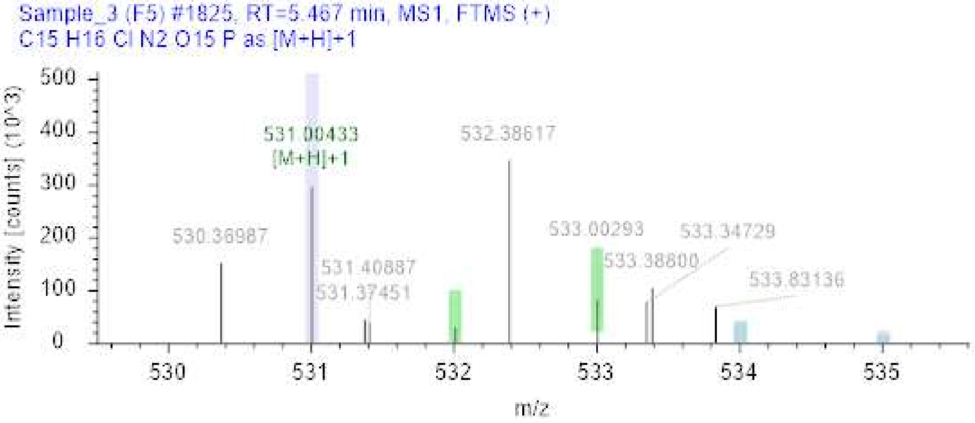

Compounds

17-Dec-2025 1:28

File name: 02_Cytb signature generation-(1)

Study: 02_Cytb signature generation

Structure	Name	RT [min]	Formula	Calc. MW	Group Areas
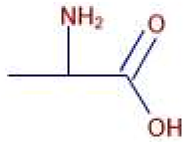	Alanine	5.47	C3 H7 N O2	89.04754	1.87e8 / 8.41e7

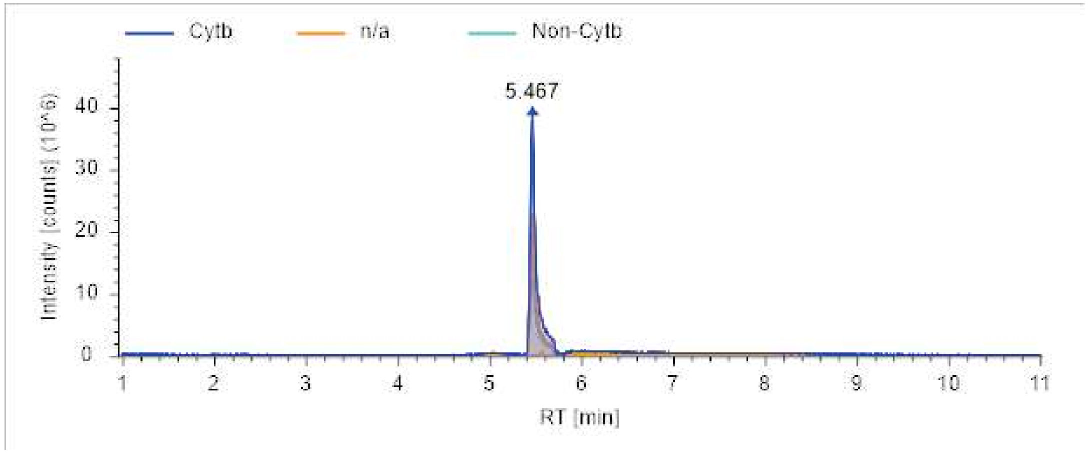

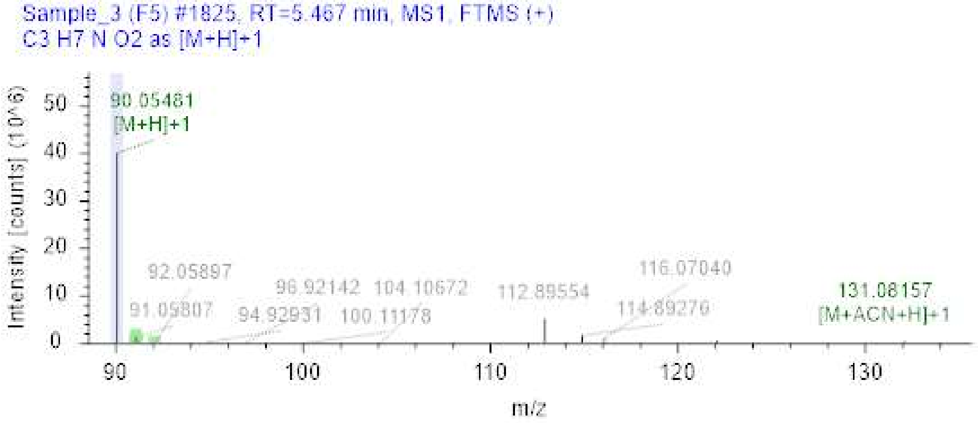

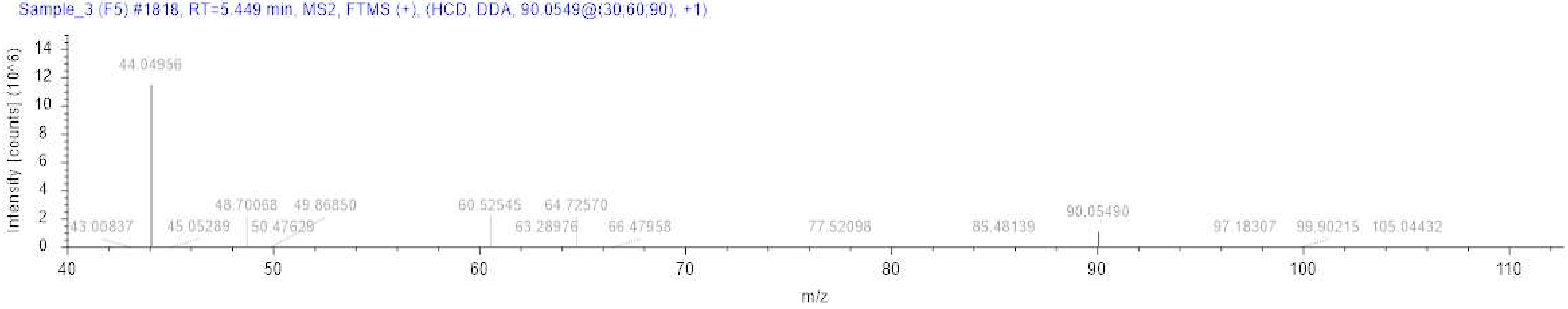

Compounds

17-Dec-2025 1:28

File name: 02_Cytb signature generation-(1)

Study: 02_Cytb signature generation

Structure	Name	RT [min]	Formula	Calc. MW	Group Areas
		5.47	C21 H16 Cl N4 O10 P3 S	643.94870	1.14e6 / 3.97e5

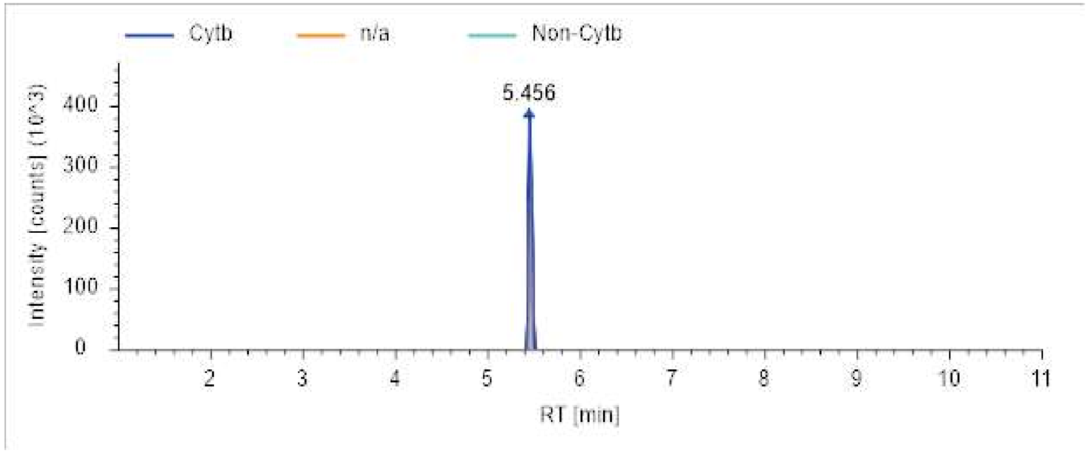

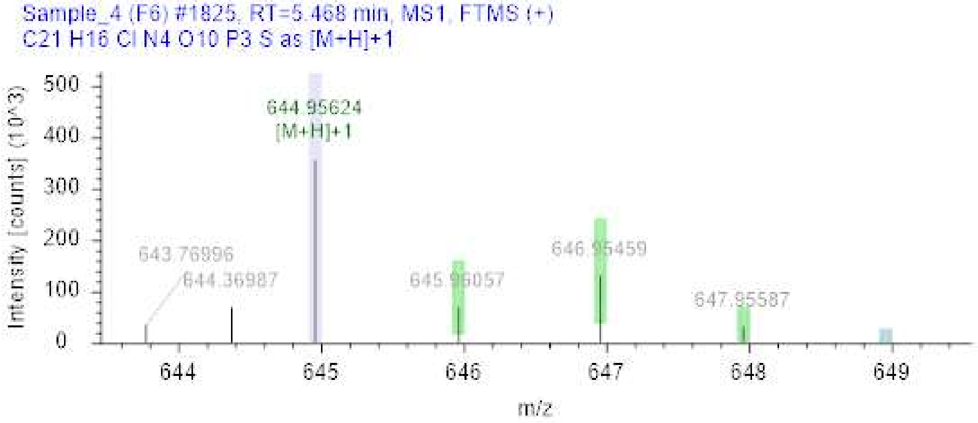

Compounds

17-Dec-2025 1:28

File name: 02_Cytb signature generation-(1)

Study: 02_Cytb signature generation

Structure	Name	RT [min]	Formula	Calc. MW	Group Areas
		5.47	C19 H22 Cl N5 O18 S4	770.95192	1.22e6 3.48e5

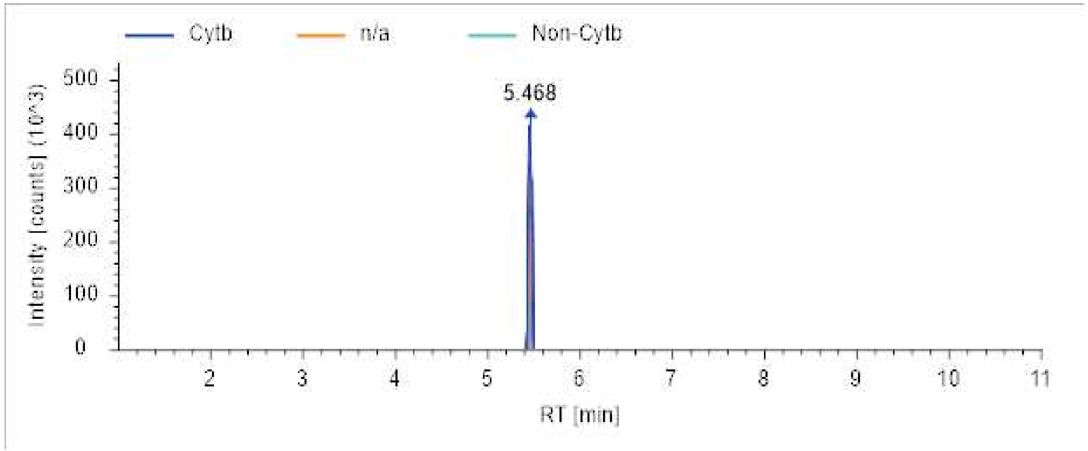

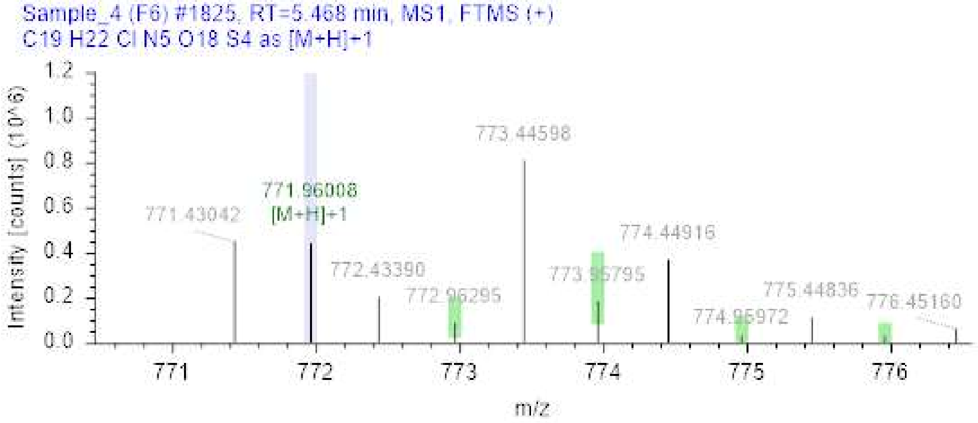

Compounds

17-Dec-2025 1:28

File name: 02_Cytb signature generation-(1)

Study: 02_Cytb signature generation

Structure	Name	RT [min]	Formula	Calc. MW	Group Areas
		5.47	C29 H17 Cl N5 O12 P3	754.97768	5.98e5 / 1.82e5

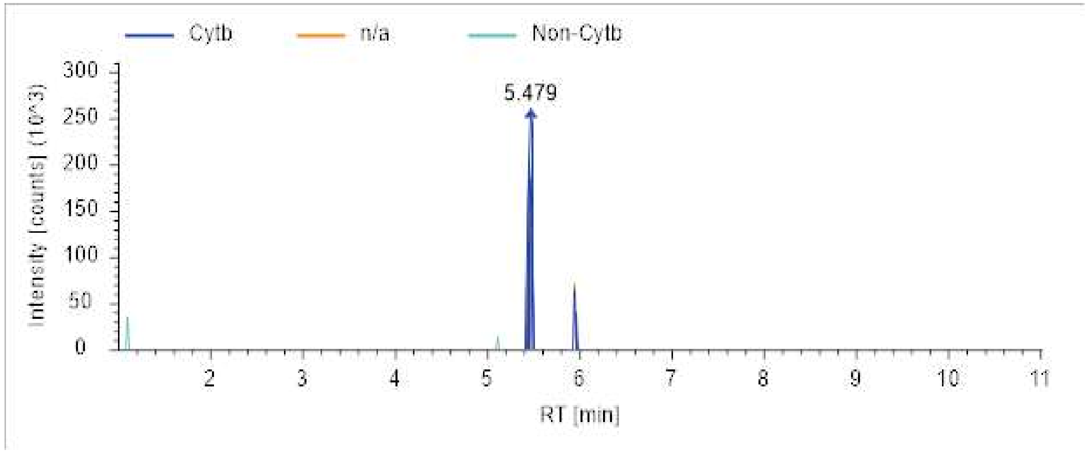

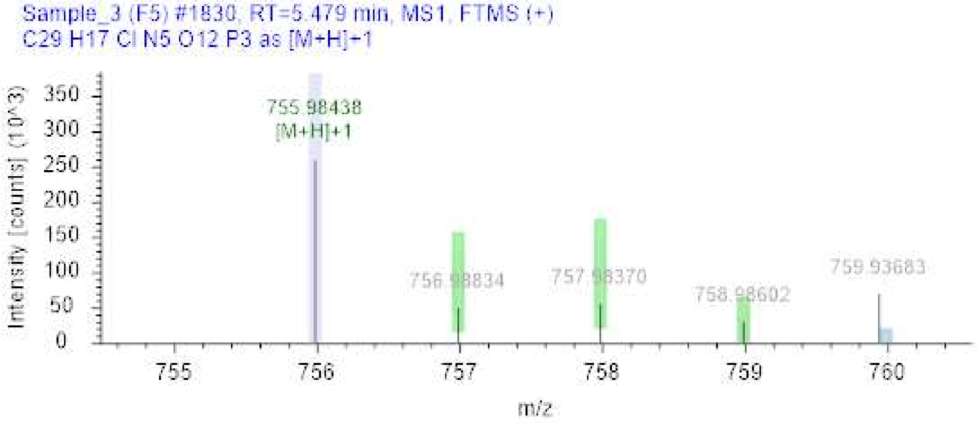

Compounds

17-Dec-2025 1:28

File name: 02_Cytb signature generation-(1)

Study: 02_Cytb signature generation

Structure	Name	RT [min]	Formula	Calc. MW	Group Areas
		5.55	C3 H N7 O6 P2	292.94722	2.69e6 4.60e5

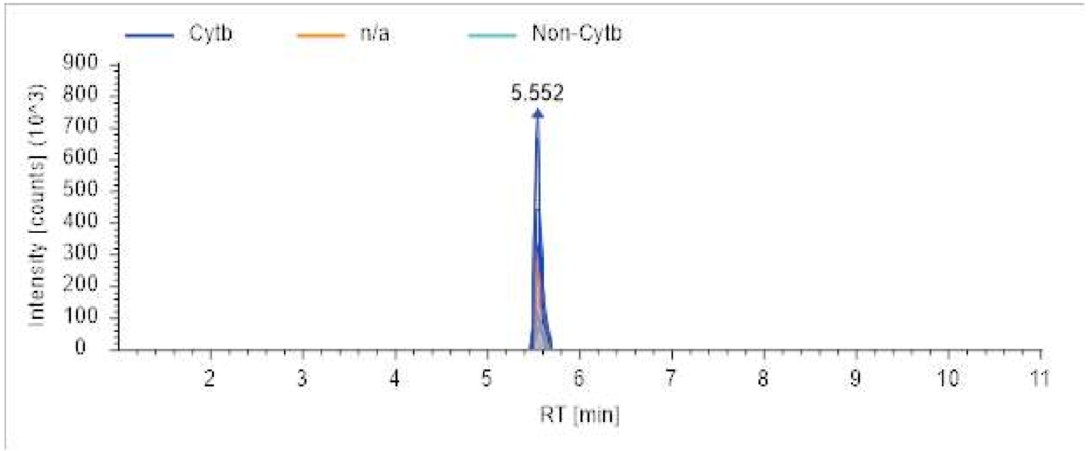

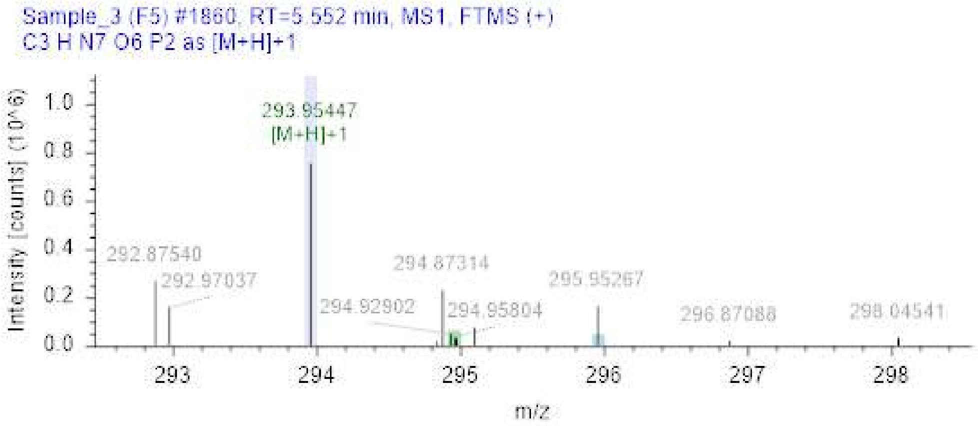

Compounds

17-Dec-2025 1:28

File name: 02_Cytb signature generation-(1)

Study: 02_Cytb signature generation

Structure	Name	RT [min]	Formula	Calc. MW	Group Areas
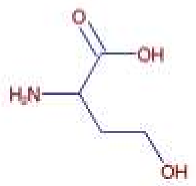	Homoserine	5.57	C4 H9 N O3	119.05811	1.95e7 3.99e6

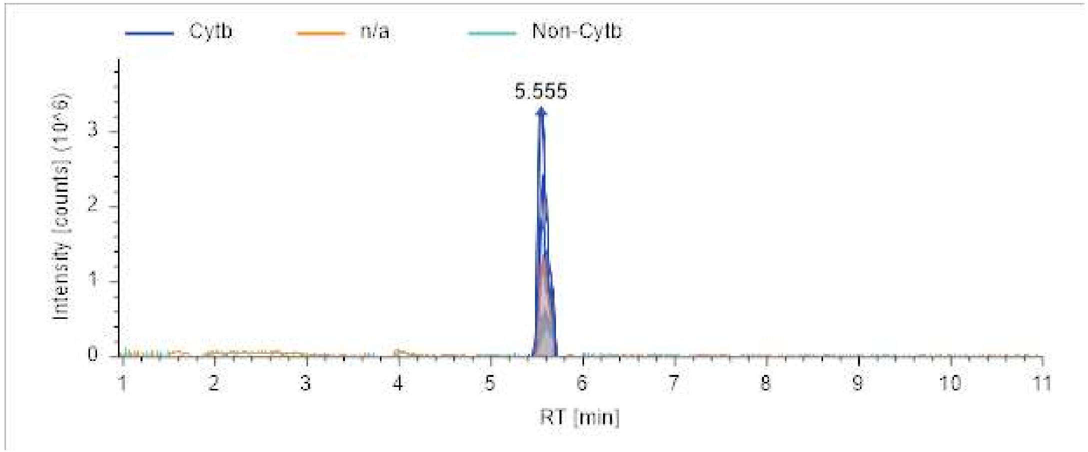

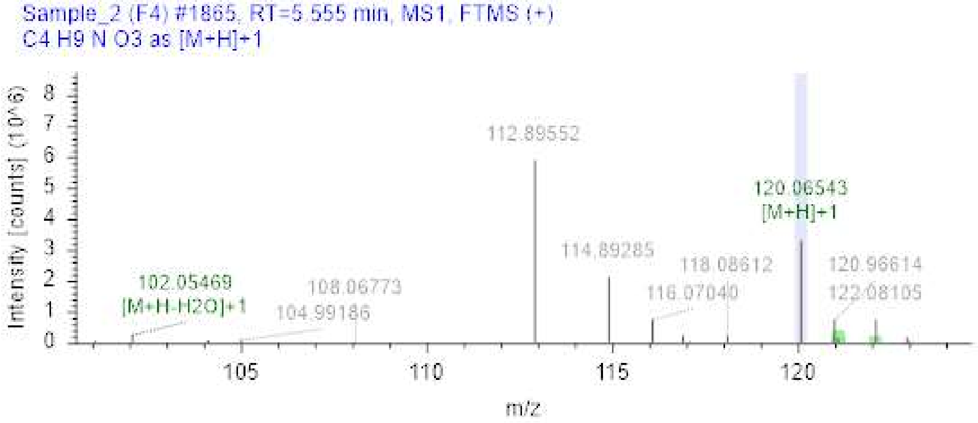

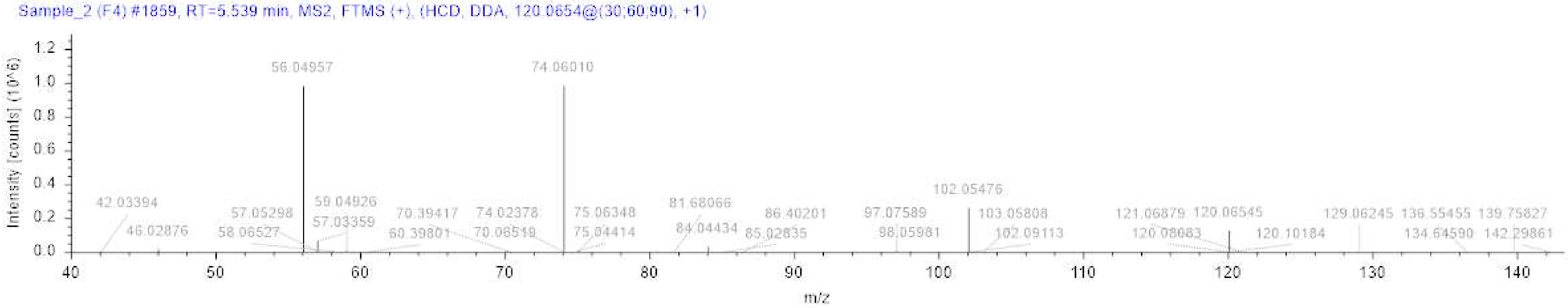

Compounds

17-Dec-2025 1:28

File name: 02_Cytb signature generation-(1)

Study: 02_Cytb signature generation

Structure	Name	RT [min]	Formula	Calc. MW	Group Areas	
		5.91	C10 H21 N8 O15 P3	586.03387	4.68e6	2.01e6

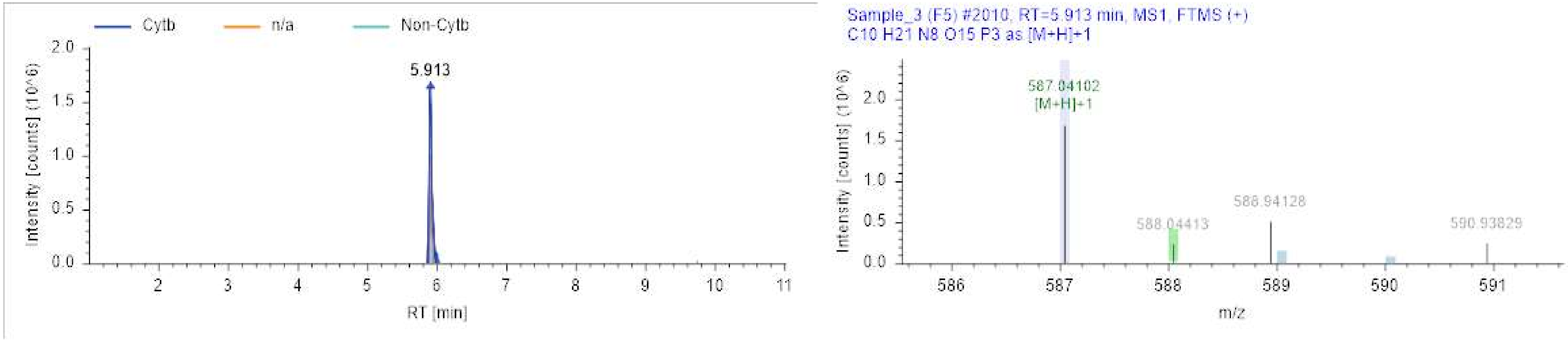

Compounds

17-Dec-2025 1:28

File name: 02_Cytb signature generation-(1)

Study: 02_Cytb signature generation

Structure	Name	RT [min]	Formula	Calc. MW	Group Areas
		5.91	C7 H23 N8 O8 P3 S2	504.03051	6.11e6 / 2.89e6

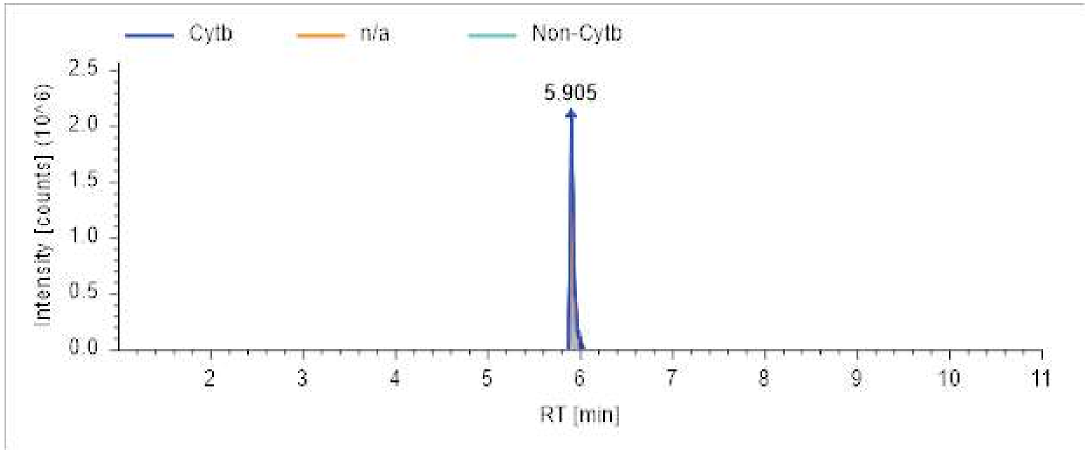

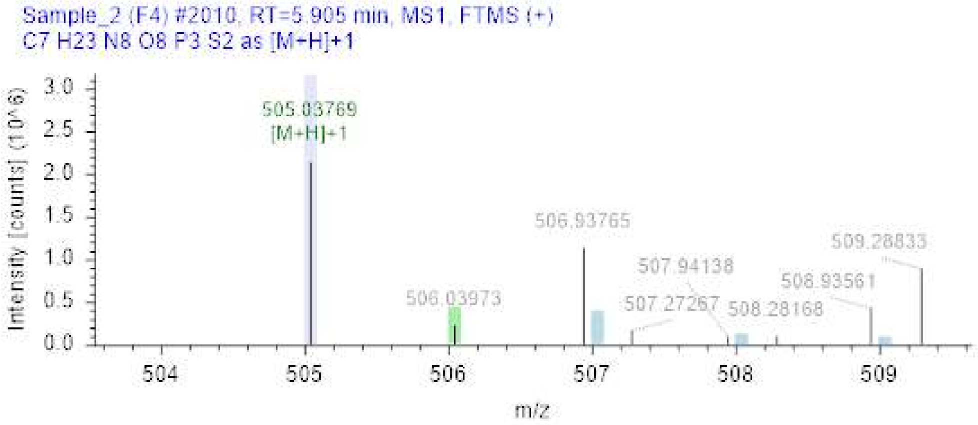

Compounds

17-Dec-2025 1:28

File name: 02_Cytb signature generation-(1)

Study: 02_Cytb signature generation

Structure	Name	RT [min]	Formula	Calc. MW	Group Areas
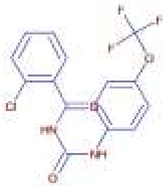		5.91	C15 H10 Cl F3 N2 O3	358.03414	1.15e7 5.28e6

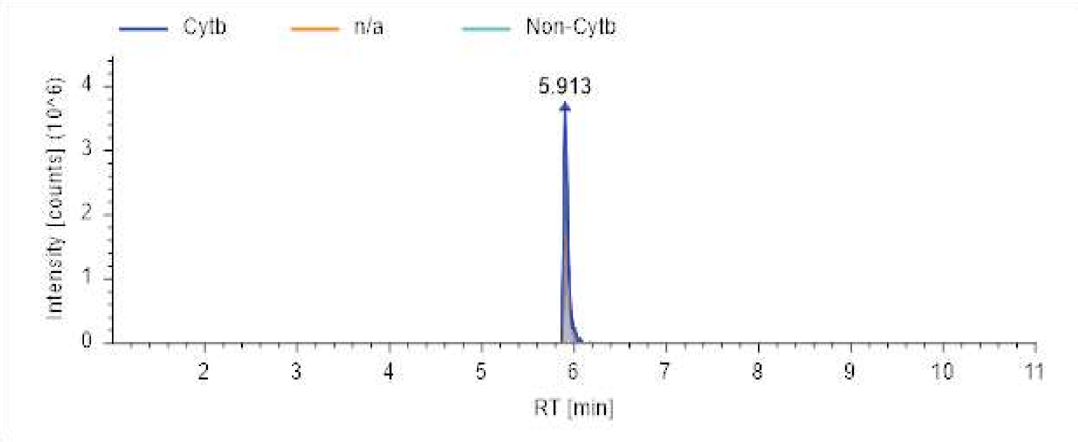

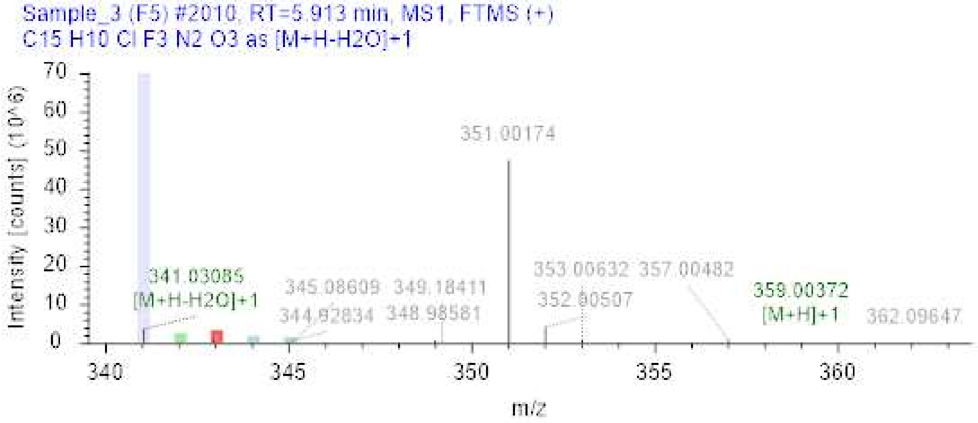

Compounds

17-Dec-2025 1:28

File name: 02_Cytb signature generation-(1)

Study: 02_Cytb signature generation

Structure	Name	RT [min]	Formula	Calc. MW	Group Areas
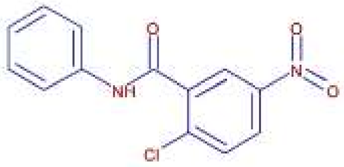		5.92	C13 H9 Cl N2 O3	276.03095	1.33e7 / 5.86e6

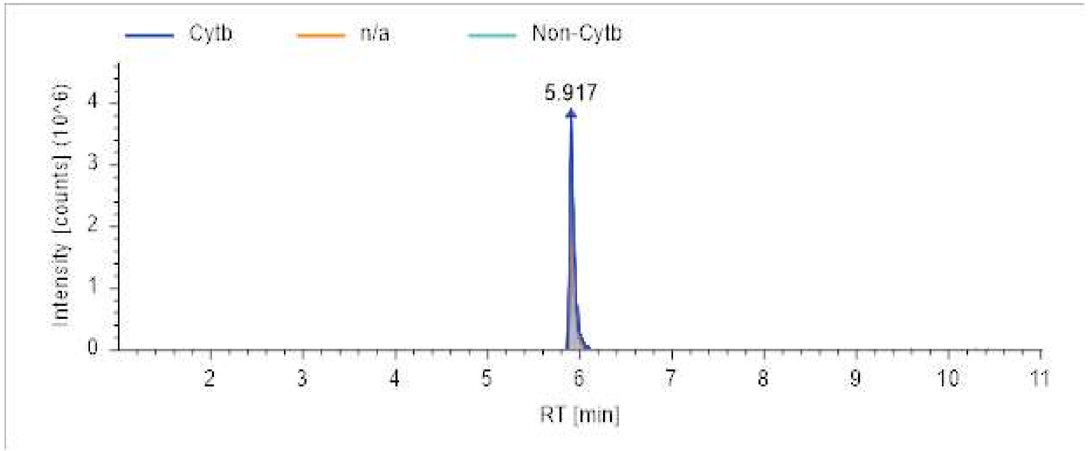

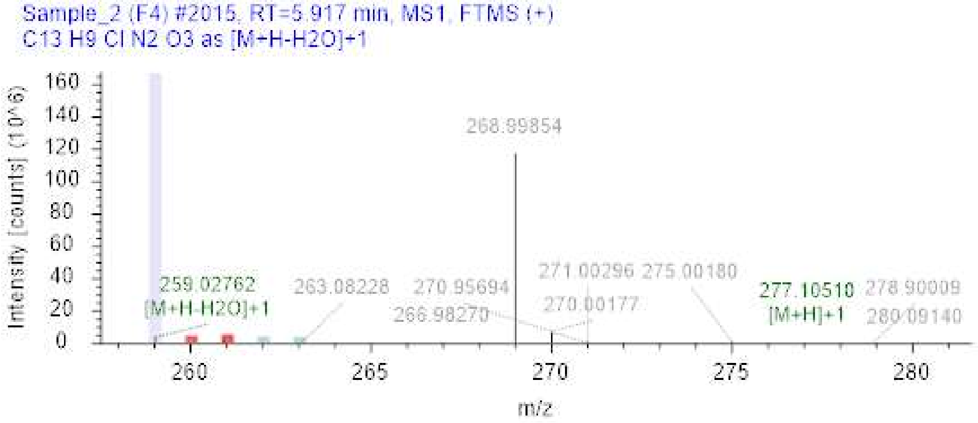

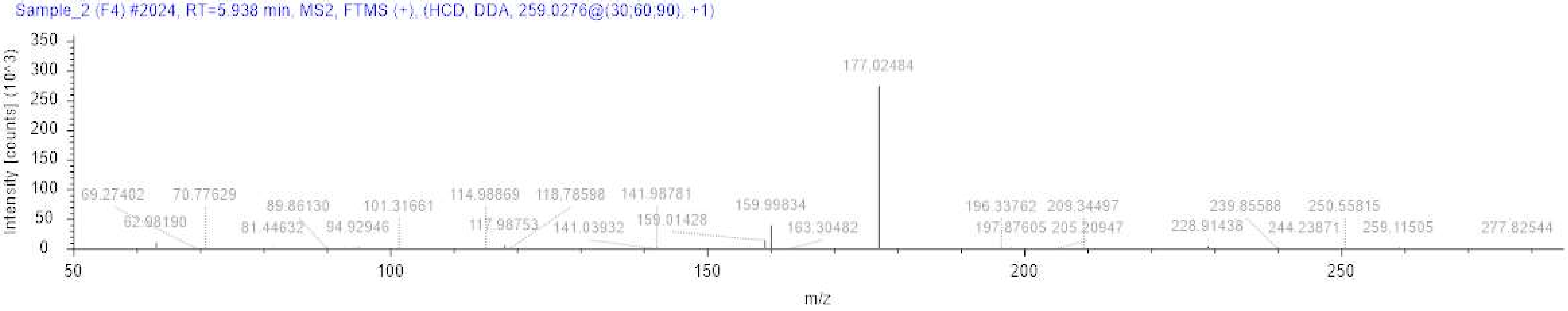

Compounds

17-Dec-2025 1:28

File name: 02_Cytb signature generation-(1)

Study: 02_Cytb signature generation

Structure	Name	RT [min]	Formula	Calc. MW	Group Areas	
		5.92	C13 H16 N2 O10 P2	422.02682	8.17e6	3.45e6

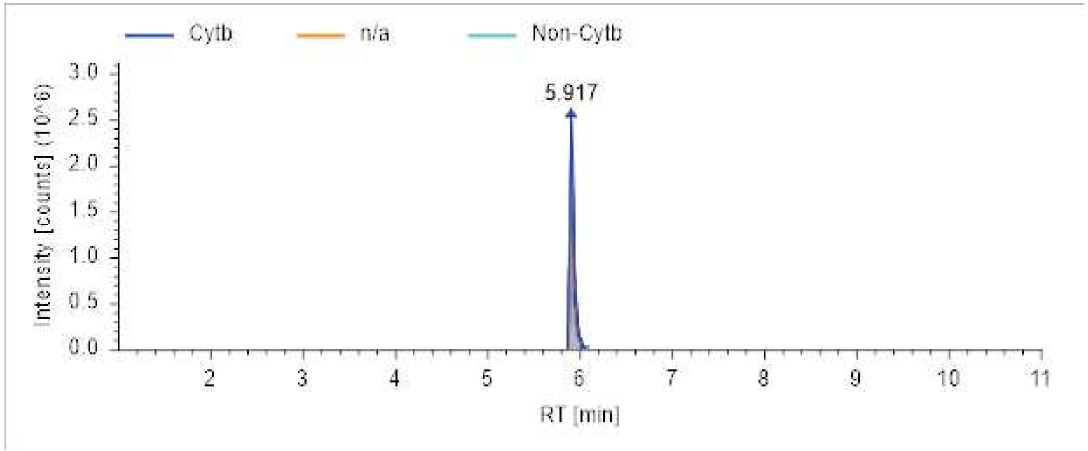

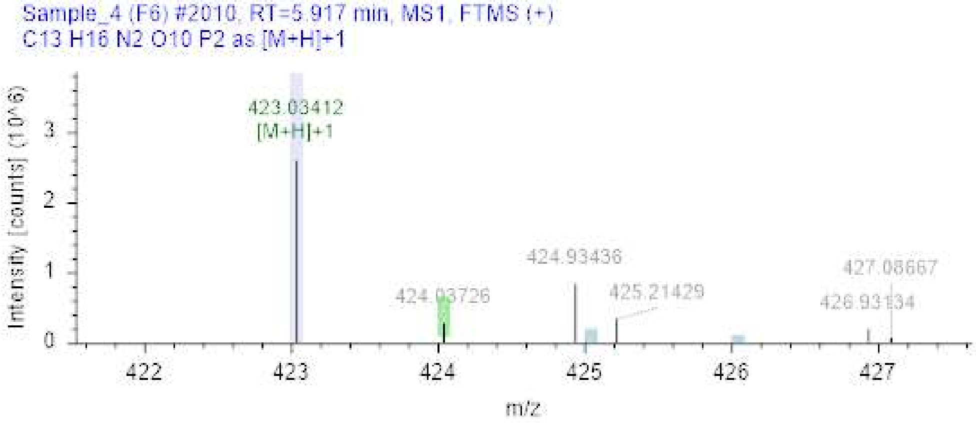

Compounds

17-Dec-2025 1:28

File name: 02_Cytb signature generation-(1)

Study: 02_Cytb signature generation

Structure	Name	RT [min]	Formula	Calc. MW	Group Areas
		5.92	C6 H10 Cl N4 O5 P S	315.97968	1.63e6 / 7.86e5

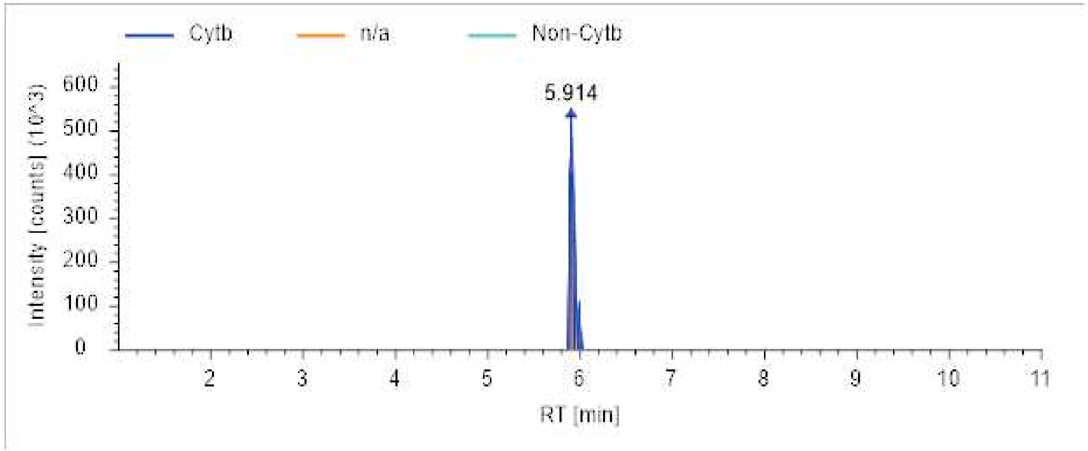

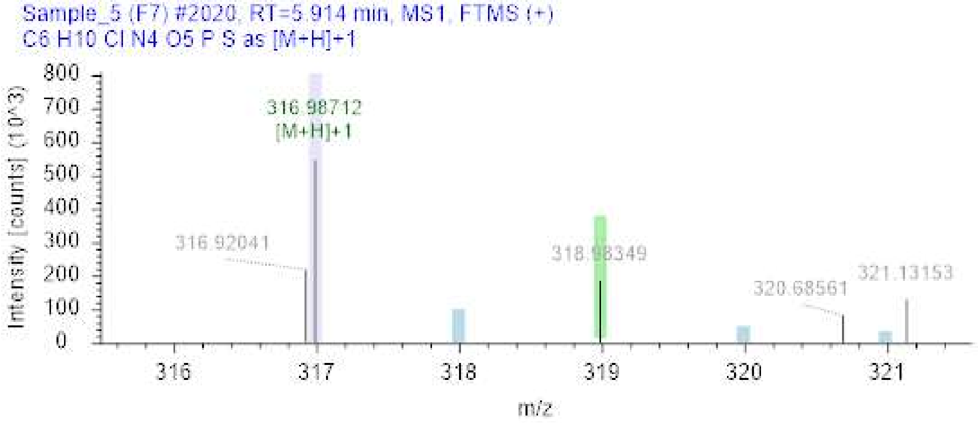

Compounds

17-Dec-2025 1:28

File name: 02_Cytb signature generation-(1)

Study: 02_Cytb signature generation

Structure	Name	RT [min]	Formula	Calc. MW	Group Areas
		5.92	C8 H13 N4 O7 P S	340.02361	1.15e7 / 5.28e6

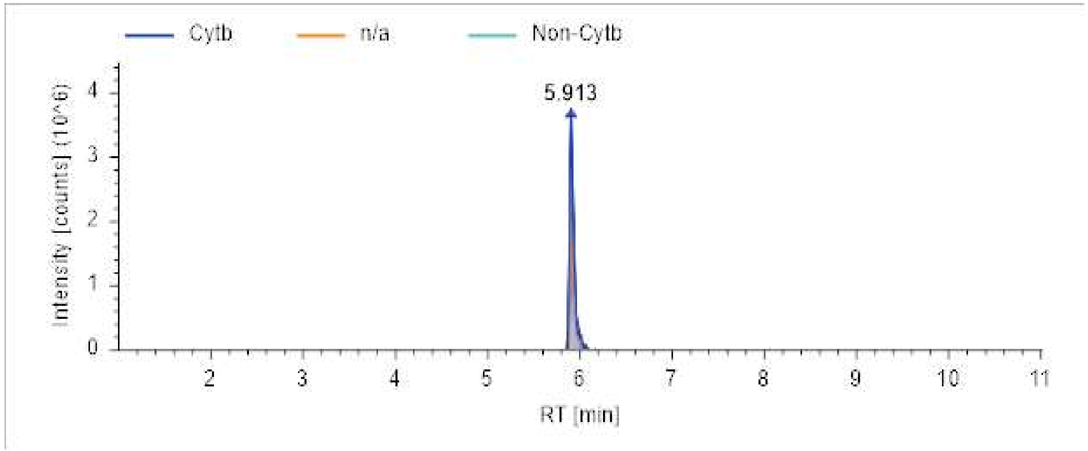

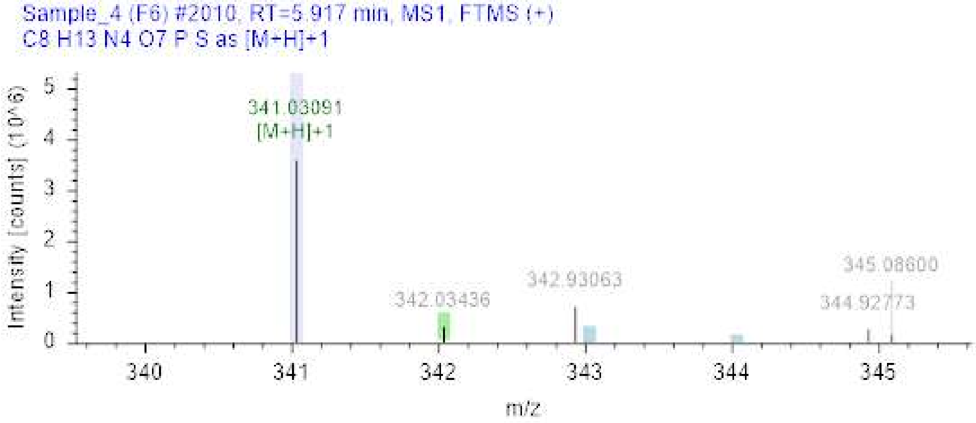

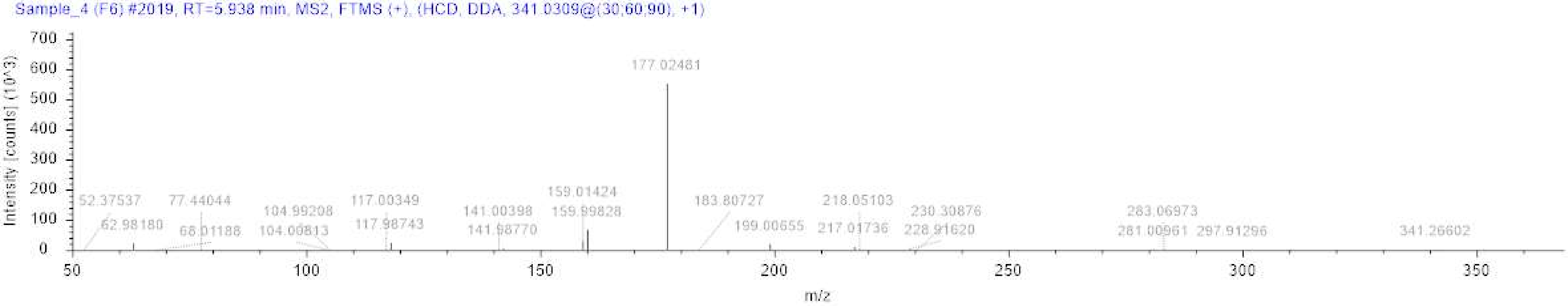

Compounds

17-Dec-2025 1:28

File name: 02_Cytb signature generation-(1)

Study: 02_Cytb signature generation

Structure	Name	RT [min]	Formula	Calc. MW	Group Areas
		5.92	C5 H15 N6 O18 P3 S2	603.90882	8.68e5 / 2.10e6

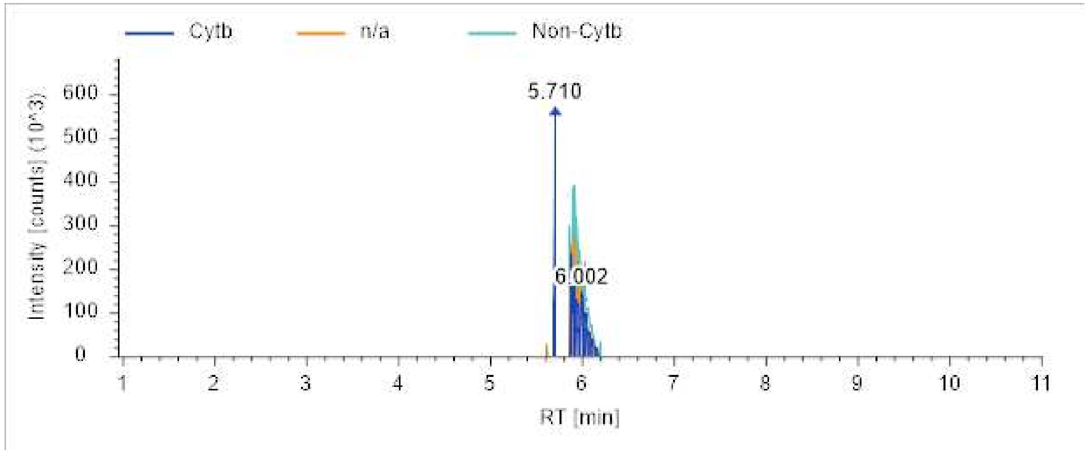

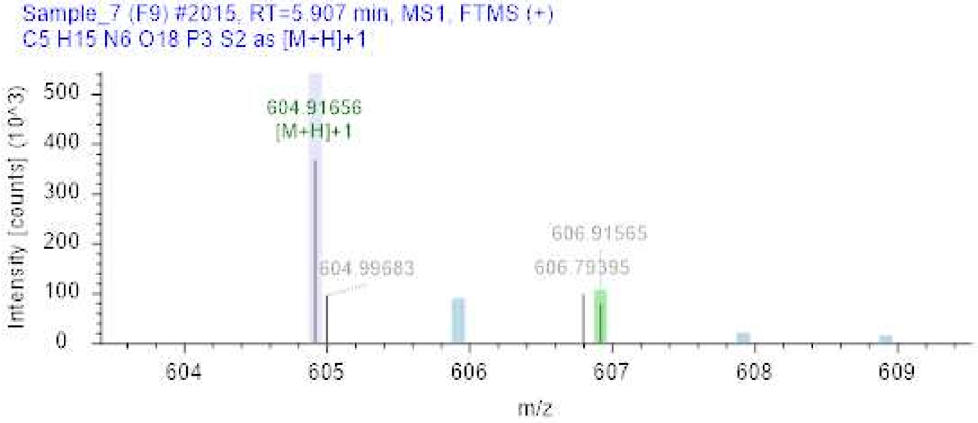

Compounds

17-Dec-2025 1:28

File name: 02_Cytb signature generation-(1)

Study: 02_Cytb signature generation

Structure	Name	RT [min]	Formula	Calc. MW	Group Areas
		5.92	C5 H7 N8 O P S	258.02032	1.33e7 / 5.86e6

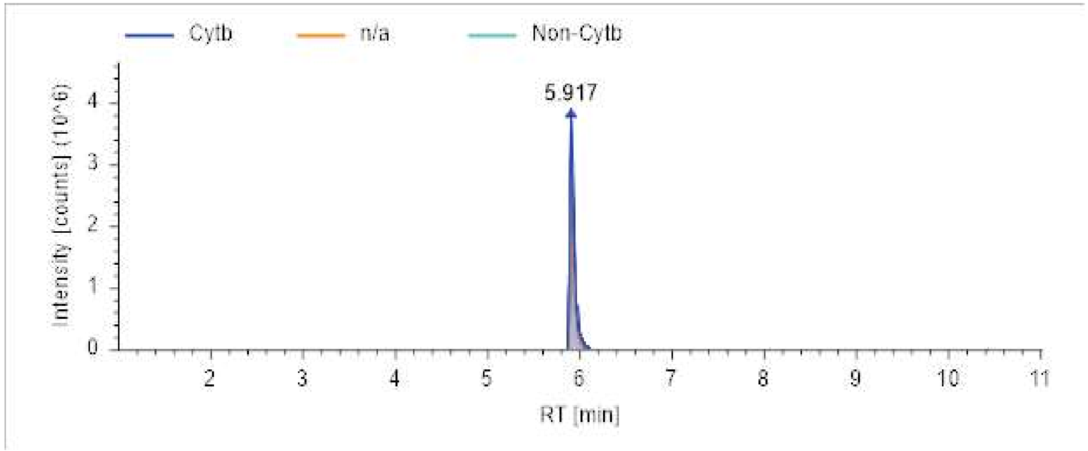

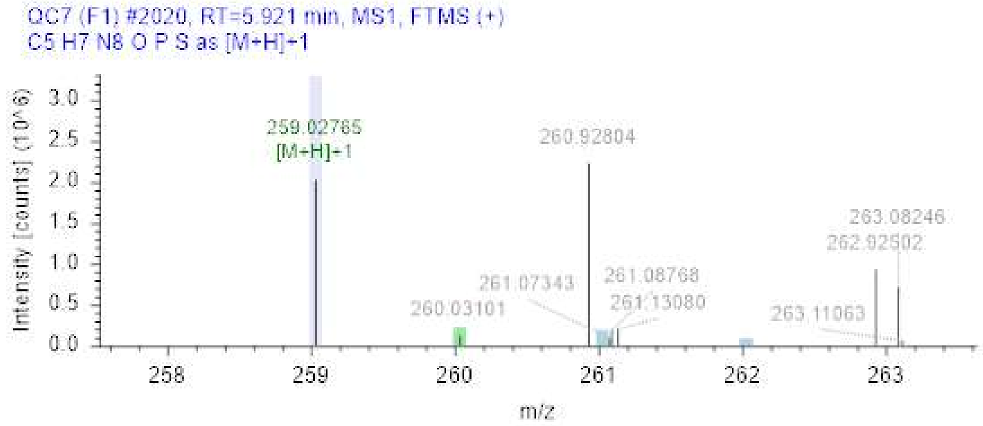

Compounds

17-Dec-2025 1:28

File name: 02_Cytb signature generation-(1)

Study: 02_Cytb signature generation

Structure	Name	RT [min]	Formula	Calc. MW	Group Areas
		5.92	C5 H9 N2 O P S	176.01738	4.86e7 / 2.06e7

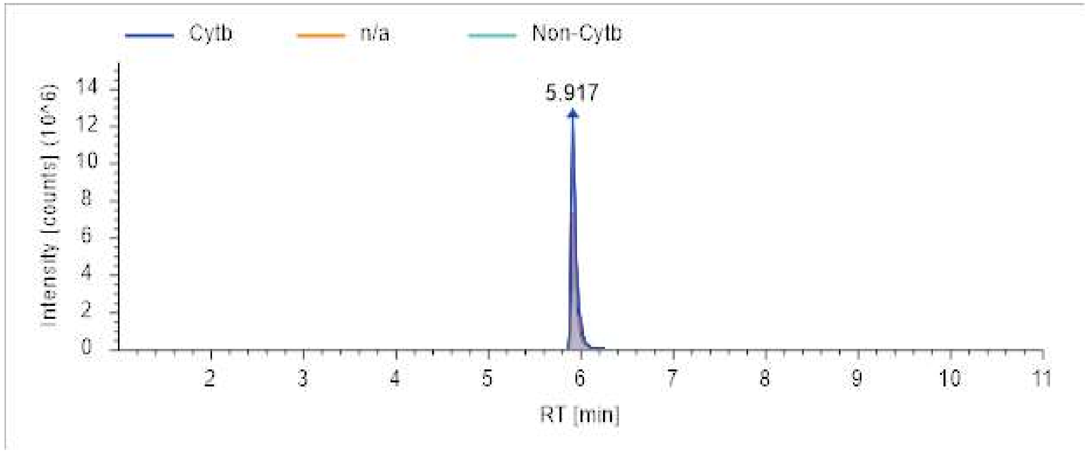

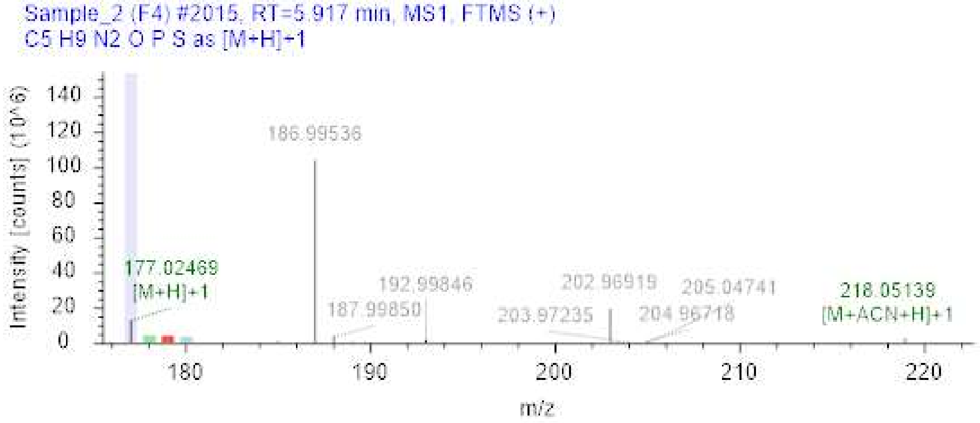

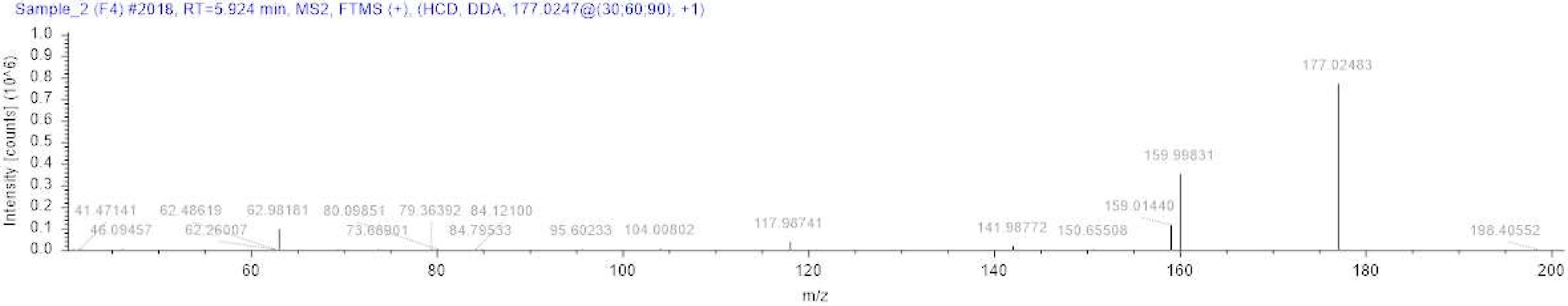

Compounds

17-Dec-2025 1:28

File name: 02_Cytb signature generation-(1)

Study: 02_Cytb signature generation

Structure	Name	RT [min]	Formula	Calc. MW	Group Areas	
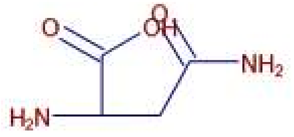	Asparagine	5.93	C4 H8 N2 O3	132.05348	3.99e7	1.75e7

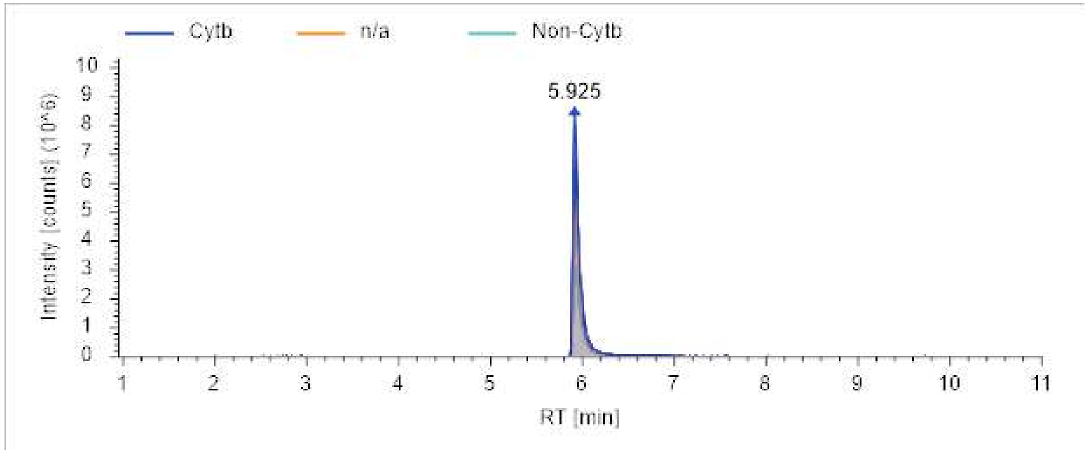

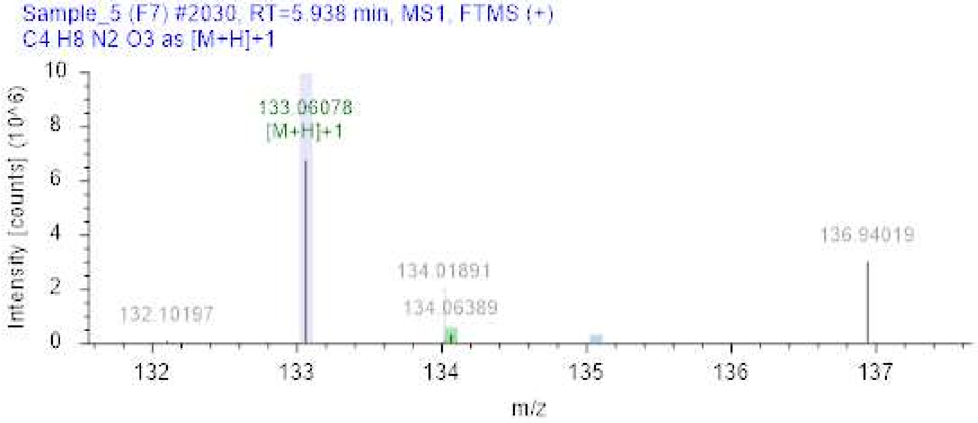

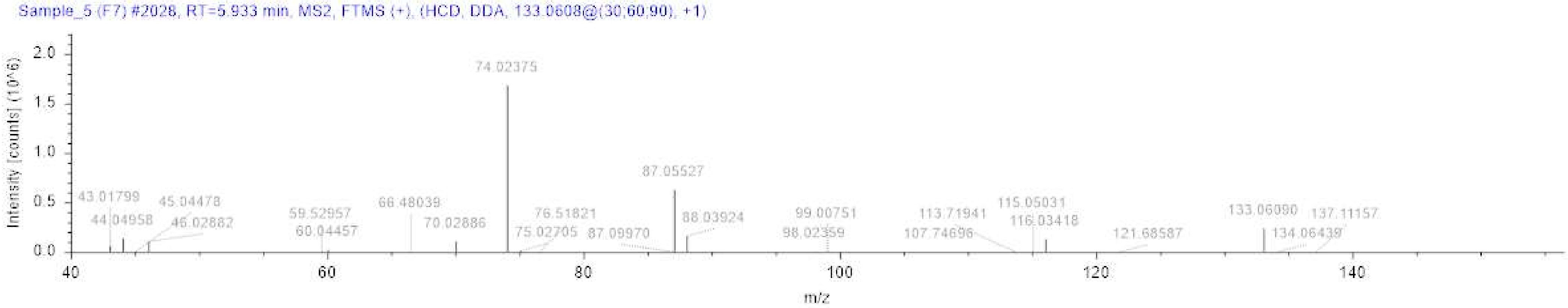

Compounds

17-Dec-2025 1:28

File name: 02_Cytb signature generation-(1)

Study: 02_Cytb signature generation

Structure	Name	RT [min]	Formula	Calc. MW	Group Areas
		5.94	C9 H18 N7 O17 P S	559.02259	3.83e6 / 1.31e6

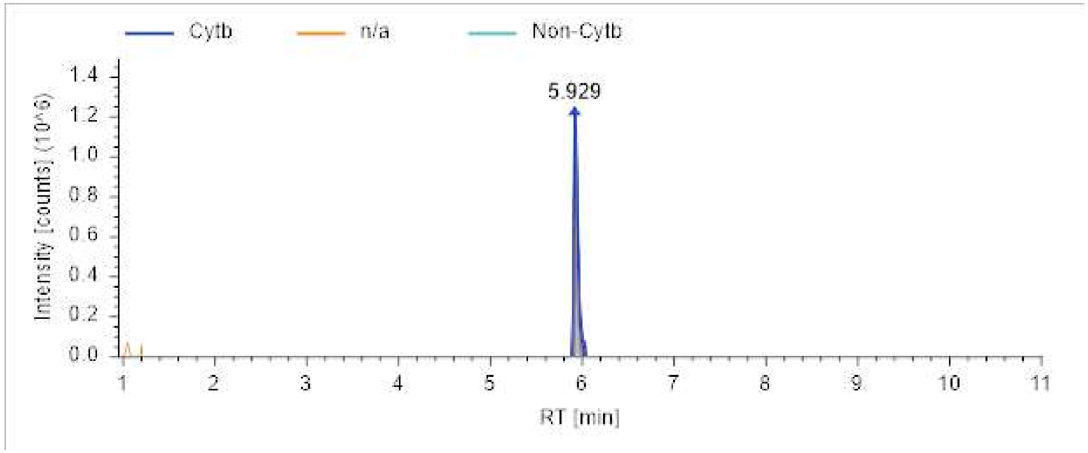

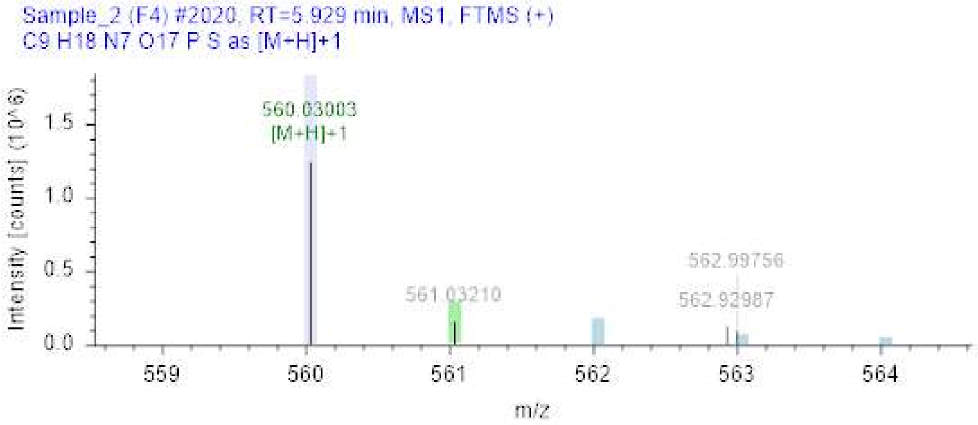

Compounds

17-Dec-2025 1:28

File name: 02_Cytb signature generation-(1)

Study: 02_Cytb signature generation

Structure	Name	RT [min]	Formula	Calc. MW	Group Areas	
		5.94	C9 H22 N O15 P3	477.01926	5.62e6	2.20e6

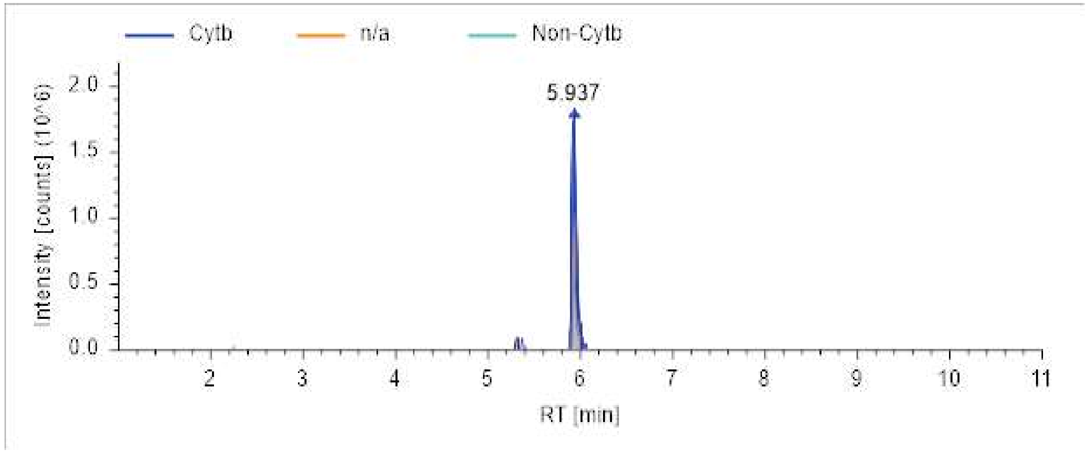

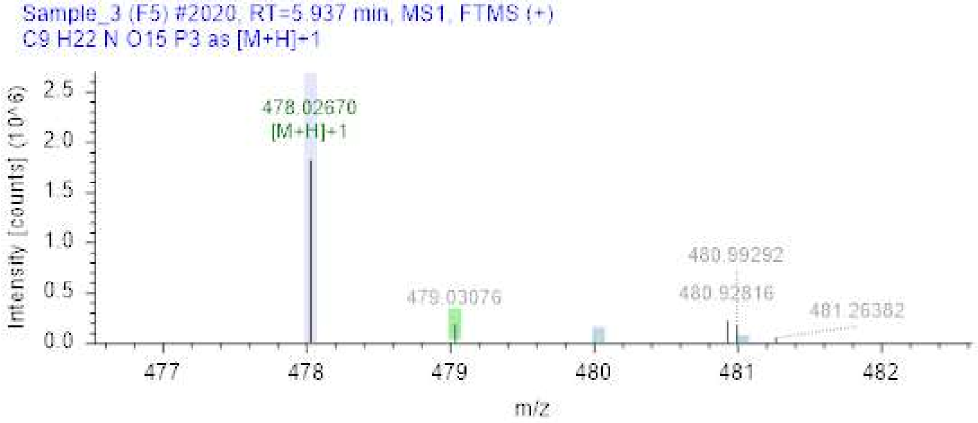

Compounds

17-Dec-2025 1:28

File name: 02_Cytb signature generation-(1)

Study: 02_Cytb signature generation

Structure	Name	RT [min]	Formula	Calc. MW	Group Areas	
		5.94	C7 H19 Cl N O13 P3	452.97471	9.82e5	3.71e5

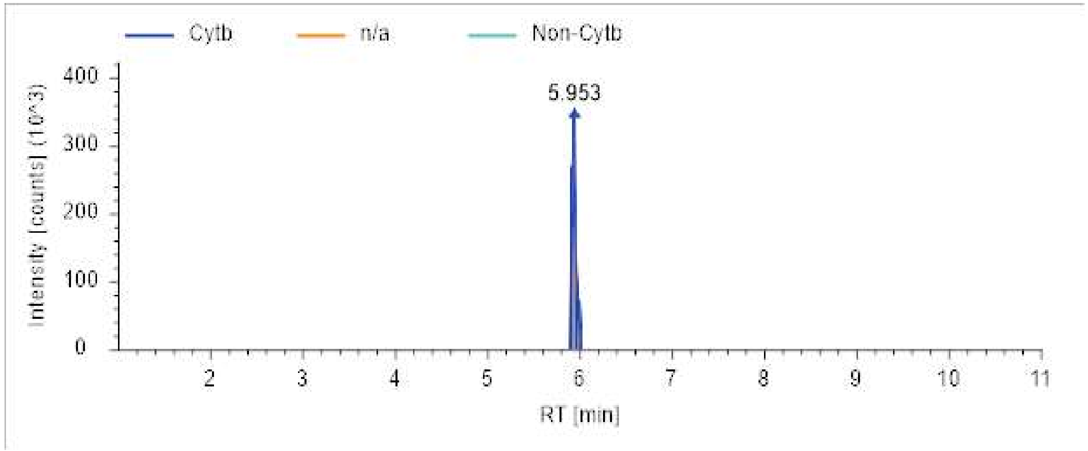

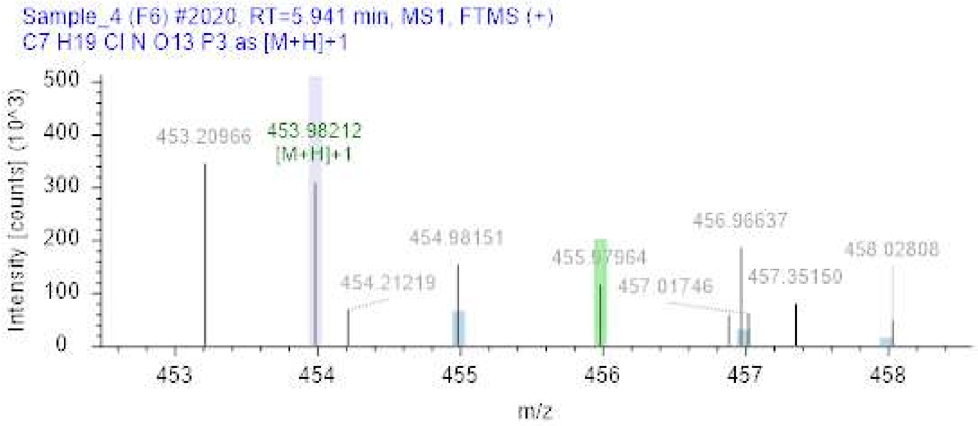

Compounds

17-Dec-2025 1:28

File name: 02_Cytb signature generation-(1)

Study: 02_Cytb signature generation

Structure	Name	RT [min]	Formula	Calc. MW	Group Areas
		5.94	C7 H12 N3 O7 P S	313.01285	1.38e7 / 4.89e6

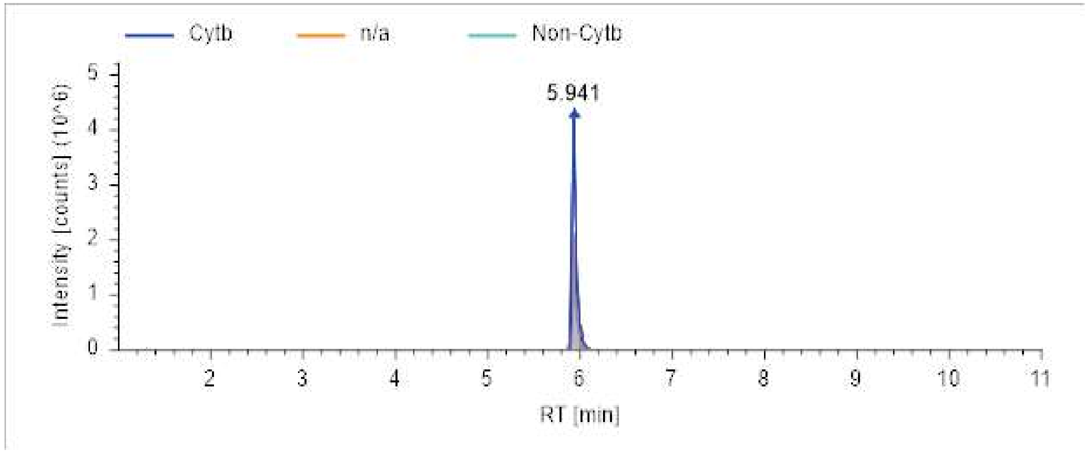

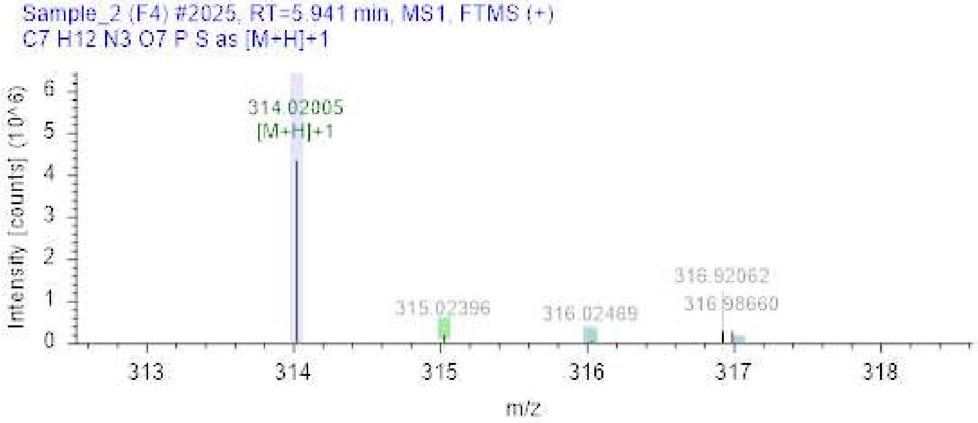

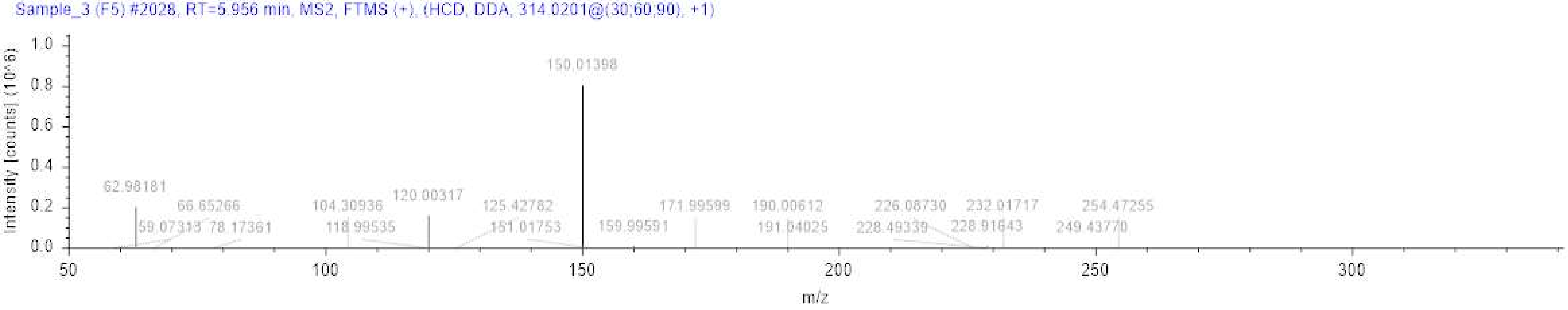

Compounds

17-Dec-2025 1:28

File name: 02_Cytb signature generation-(1)

Study: 02_Cytb signature generation

Structure	Name	RT [min]	Formula	Calc. MW	Group Areas	
		5.94	C6 H16 N5 O9 P3	395.01584	7.50e6	2.76e6

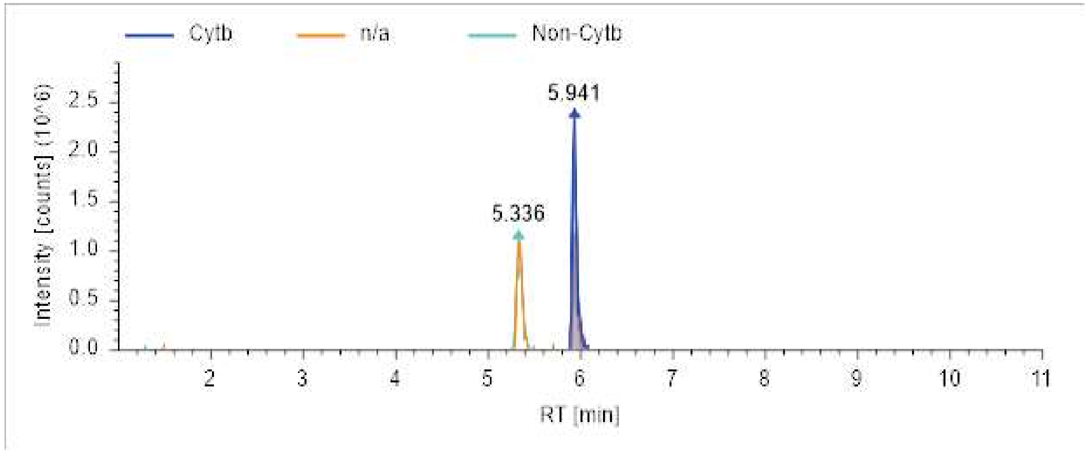

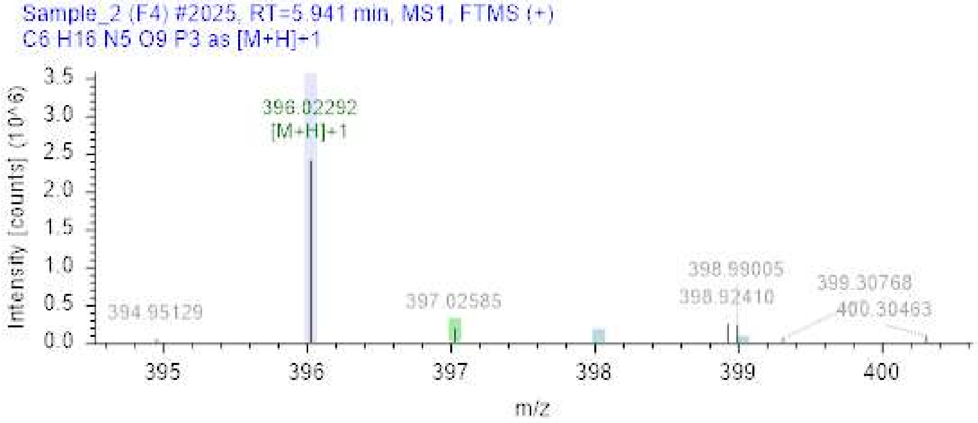

Compounds

17-Dec-2025 1:28

File name: 02_Cytb signature generation-(1)

Study: 02_Cytb signature generation

Structure	Name	RT [min]	Formula	Calc. MW	Group Areas
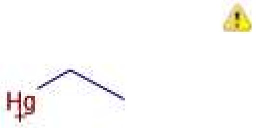	ETHYL MERCURY ION	5.94	C2 H5 Hg	231.00939	1.10e7 3.71e6

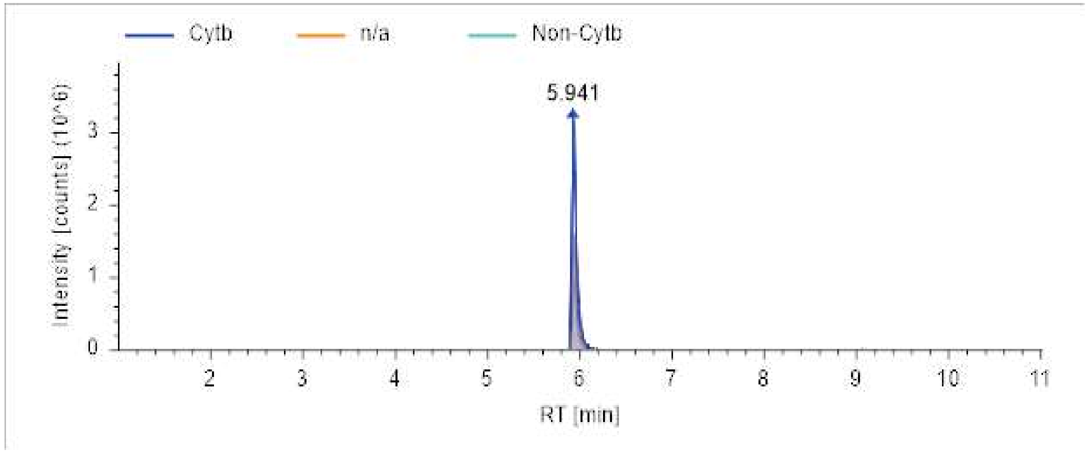

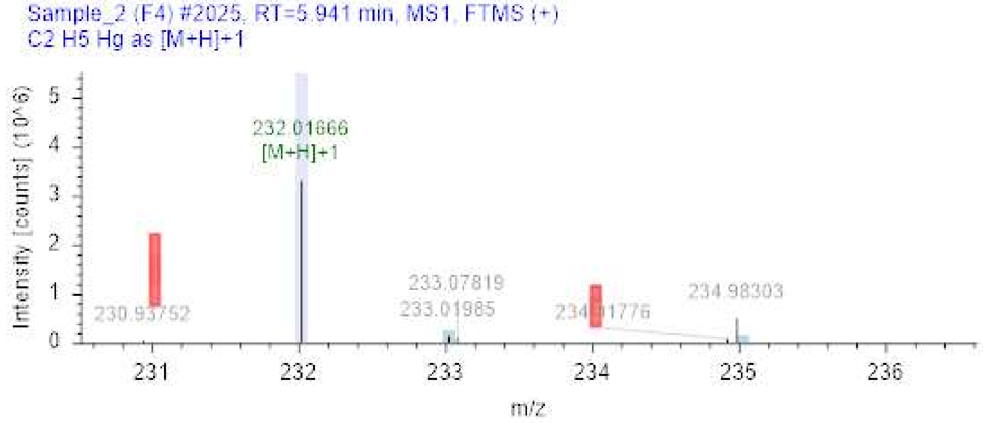

Compounds

17-Dec-2025 1:28

File name: 02_Cytb signature generation-(1)

Study: 02_Cytb signature generation

Structure	Name	RT [min]	Formula	Calc. MW	Group Areas
		5.94	C4 H13 Cl N5 O7 P3	370.97143	1.33e6 | 4.87e5

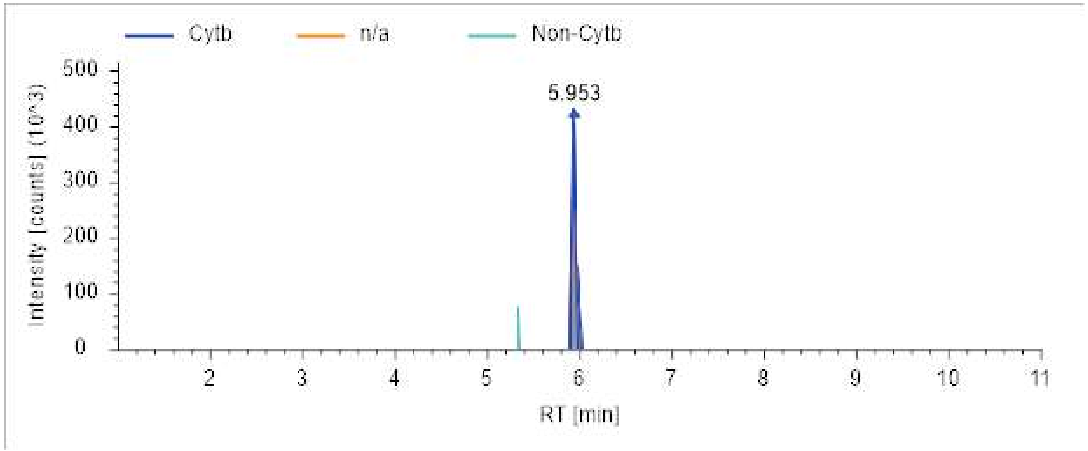

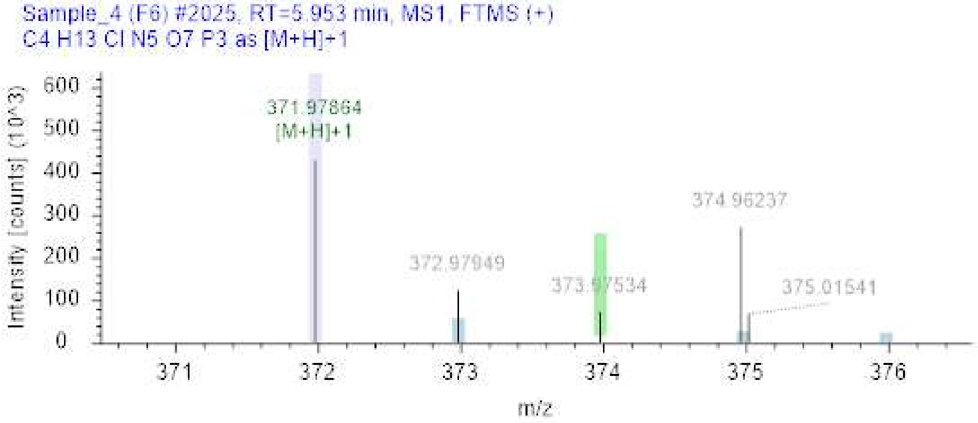

Compounds

17-Dec-2025 1:28

File name: 02_Cytb signature generation-(1)

Study: 02_Cytb signature generation

Structure	Name	RT [min]	Formula	Calc. MW	Group Areas	
		5.94	C7 H14 Cl N O P2 S2	288.96824	1.89e6	6.54e5

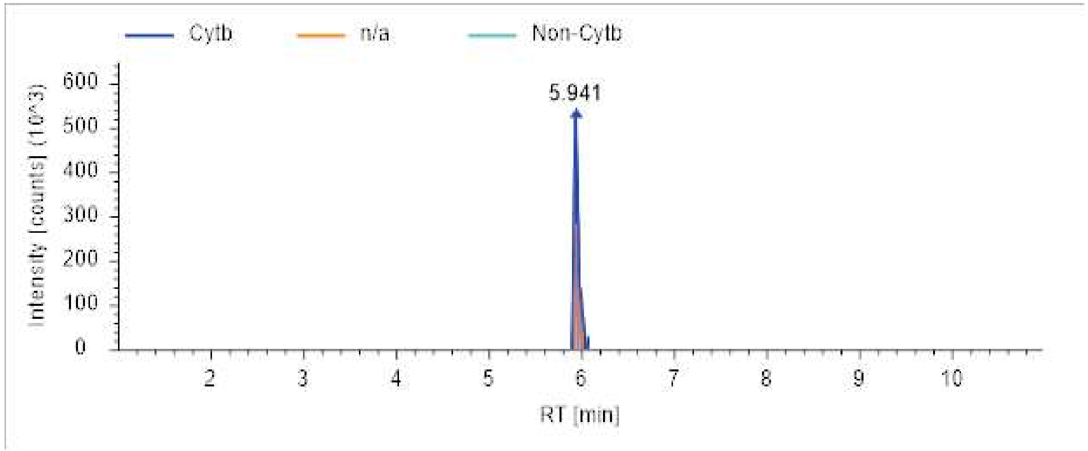

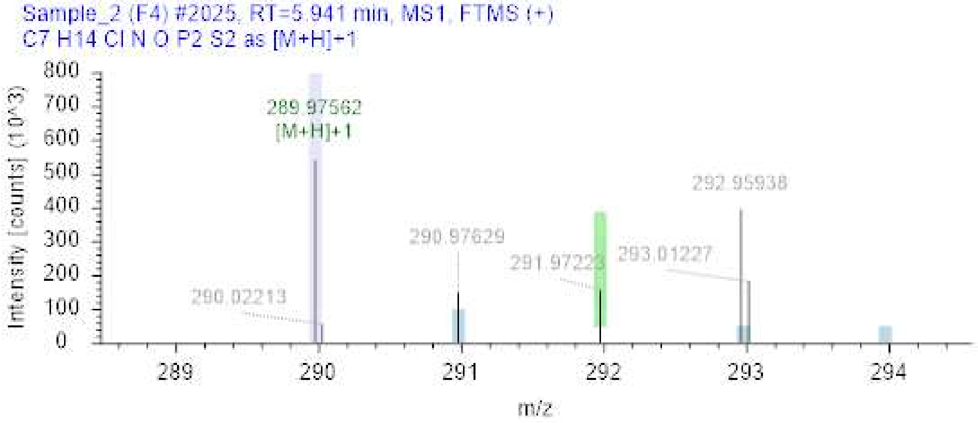

Compounds

17-Dec-2025 1:28

File name: 02_Cytb signature generation-(1)

Study: 02_Cytb signature generation

Structure	Name	RT [min]	Formula	Calc. MW	Group Areas
		5.95	C4 H8 N O P S	149.00655	3.31e7 / 1.05e7

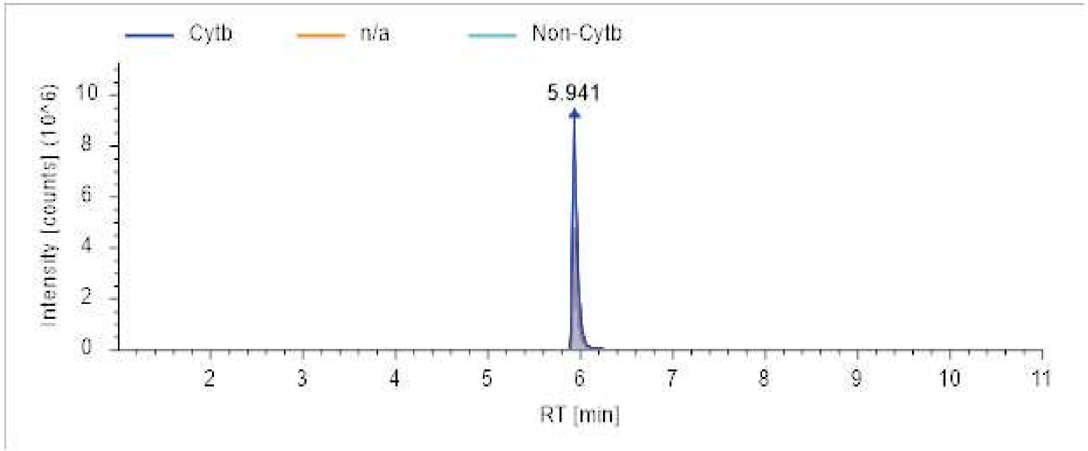

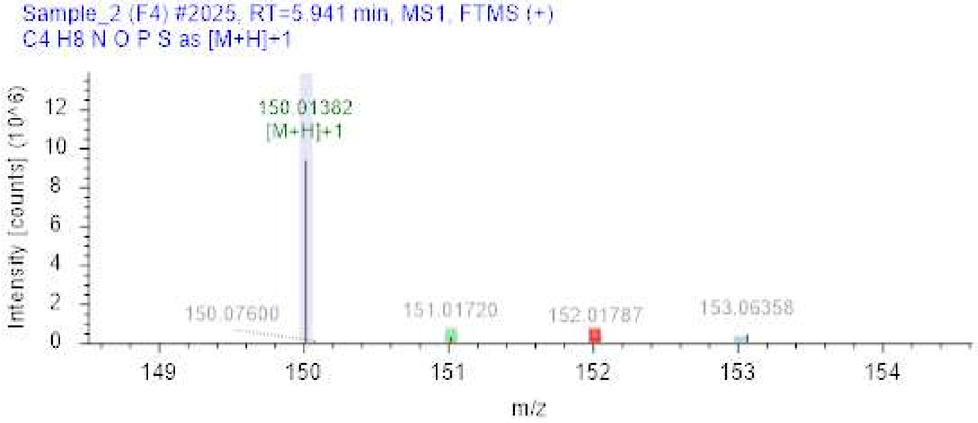

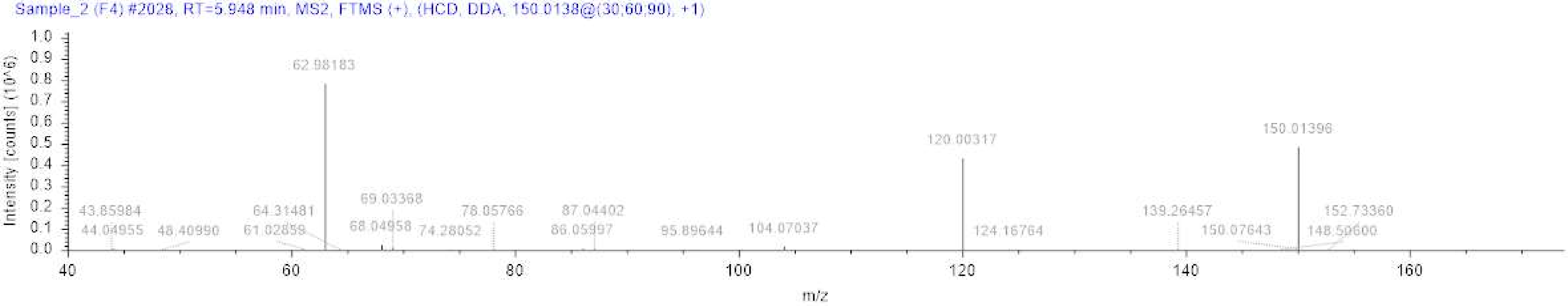

Compounds

17-Dec-2025 1:28

File name: 02_Cytb signature generation-(1)

Study: 02_Cytb signature generation

Structure	Name	RT [min]	Formula	Calc. MW	Group Areas
		5.95	C8 H11 N O9 S2	328.98691	6.65e6 2.87e6

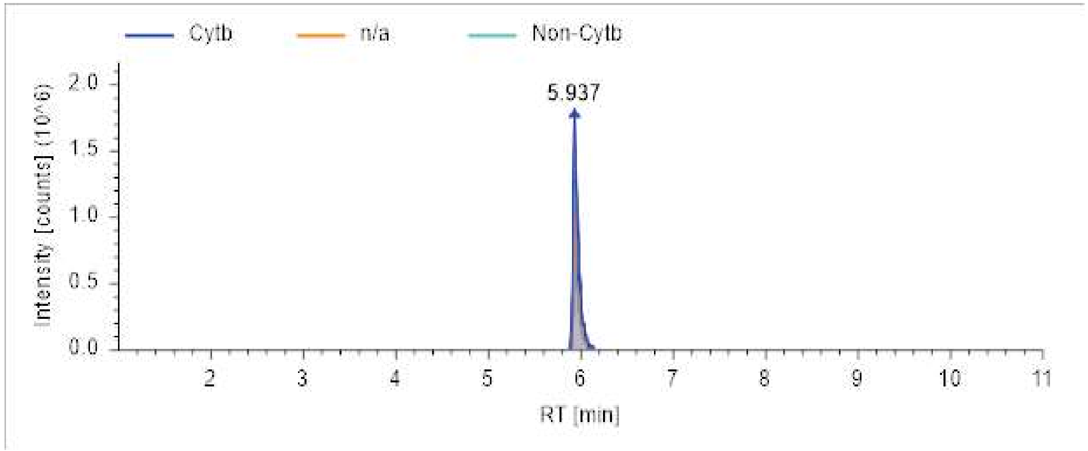

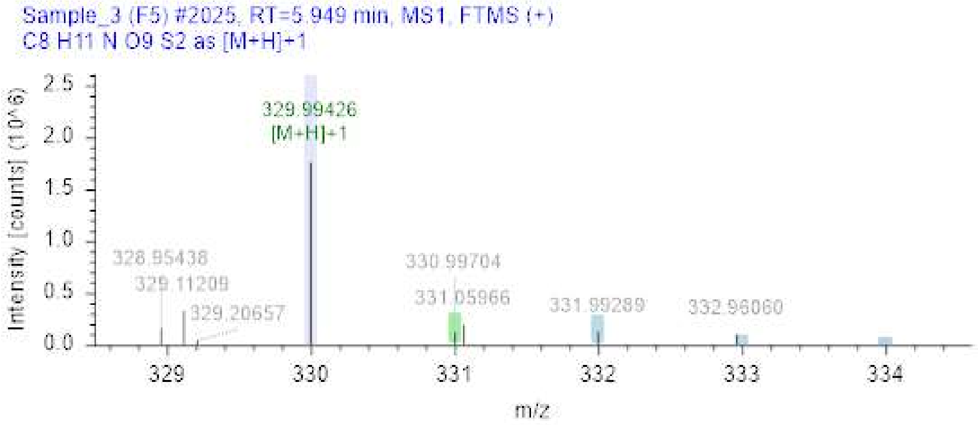

Compounds

17-Dec-2025 1:28

File name: 02_Cytb signature generation-(1)

Study: 02_Cytb signature generation

Structure	Name	RT [min]	Formula	Calc. MW	Group Areas	
		5.95	C4 H N O9	206.96481	1.83e6	6.17e5

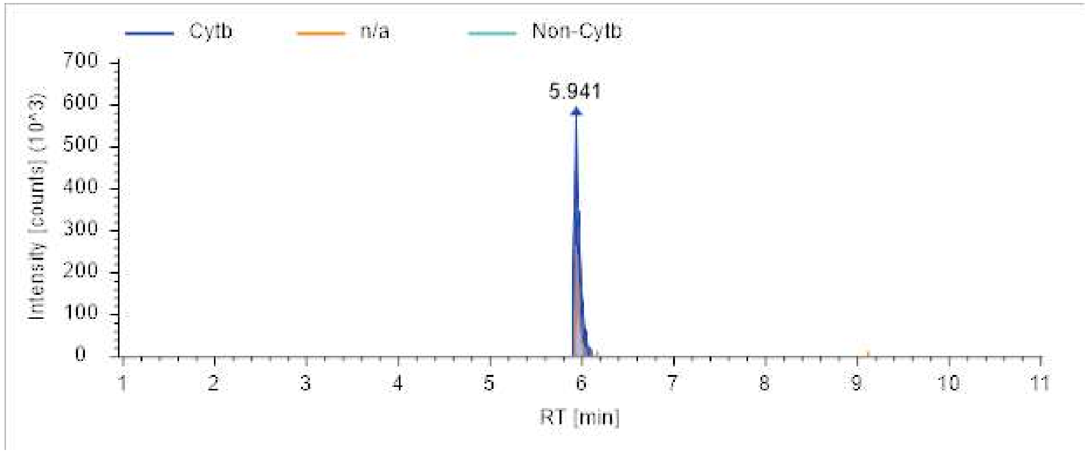

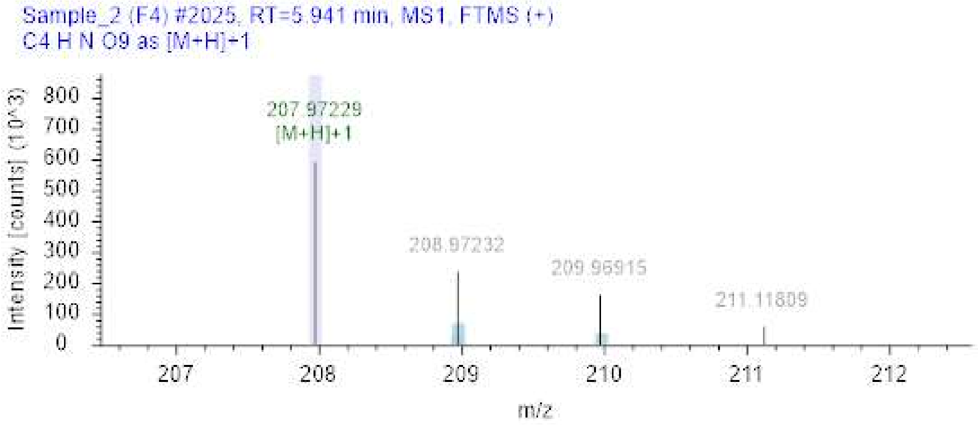

Compounds

17-Dec-2025 1:28

File name: 02_Cytb signature generation-(1)

Study: 02_Cytb signature generation

Structure	Name	RT [min]	Formula	Calc. MW	Group Areas
	SERINE	5.95	C3 H7 N O3	105.04228	1.49e7 5.08e6

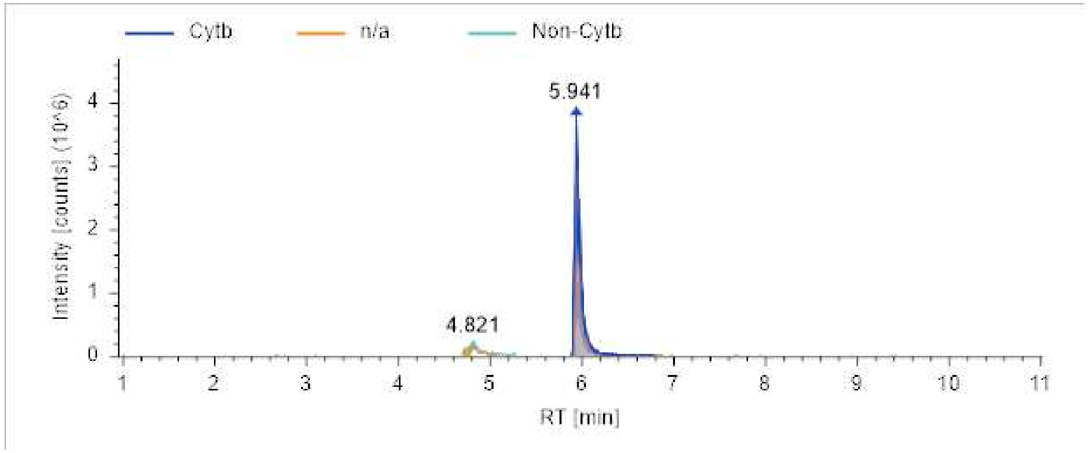

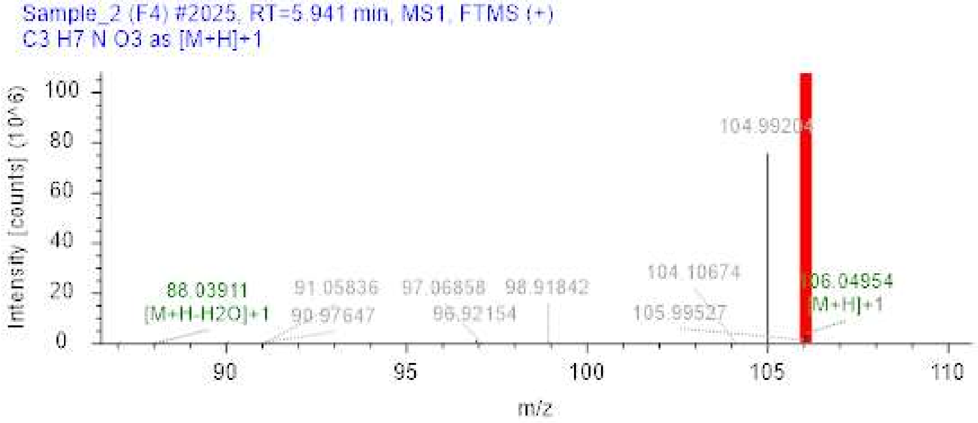

Compounds

17-Dec-2025 1:28

File name: 02_Cytb signature generation-(1)

Study: 02_Cytb signature generation

Structure	Name	RT [min]	Formula	Calc. MW	Group Areas	
		5.95	C5 H4 Cl N O12	304.94197	1.26e6	5.33e5

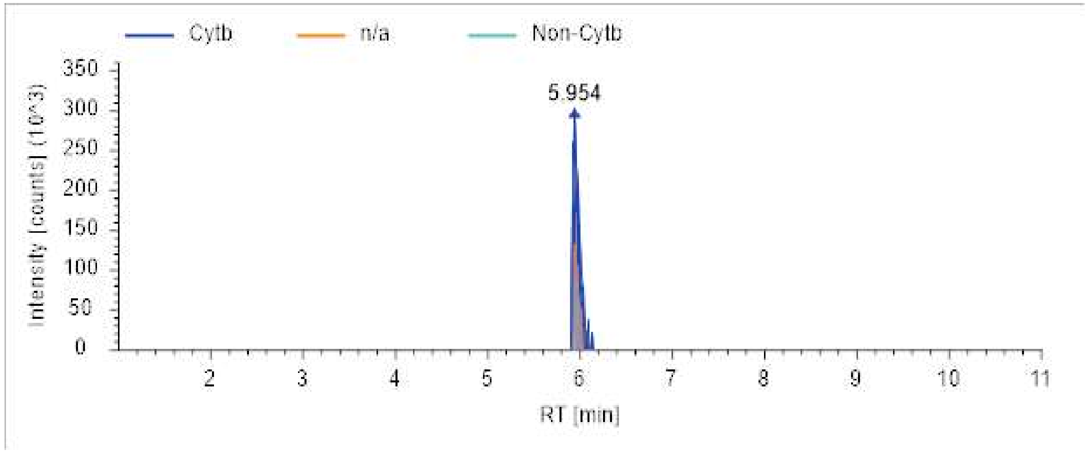

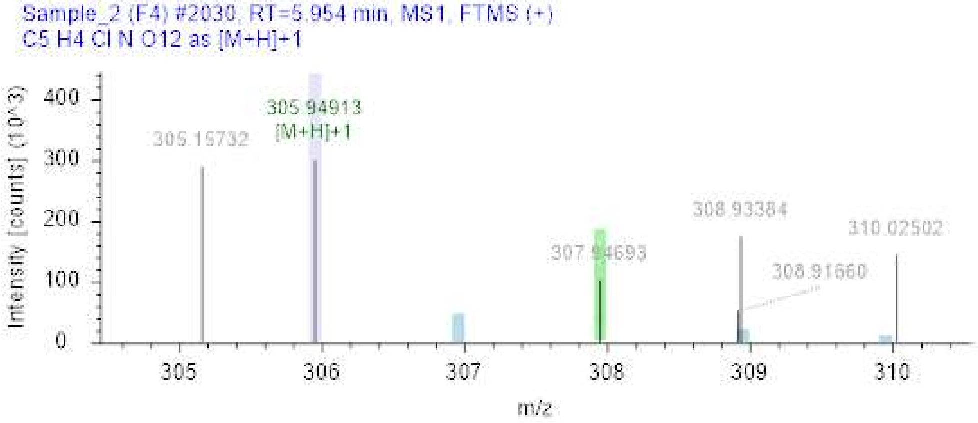

Compounds

17-Dec-2025 1:28

File name: 02_Cytb signature generation-(1)

Study: 02_Cytb signature generation

Structure	Name	RT [min]	Formula	Calc. MW	Group Areas
		5.96	C4 H5 Cl N3 P3	222.93877	1.39e6 / 5.02e5

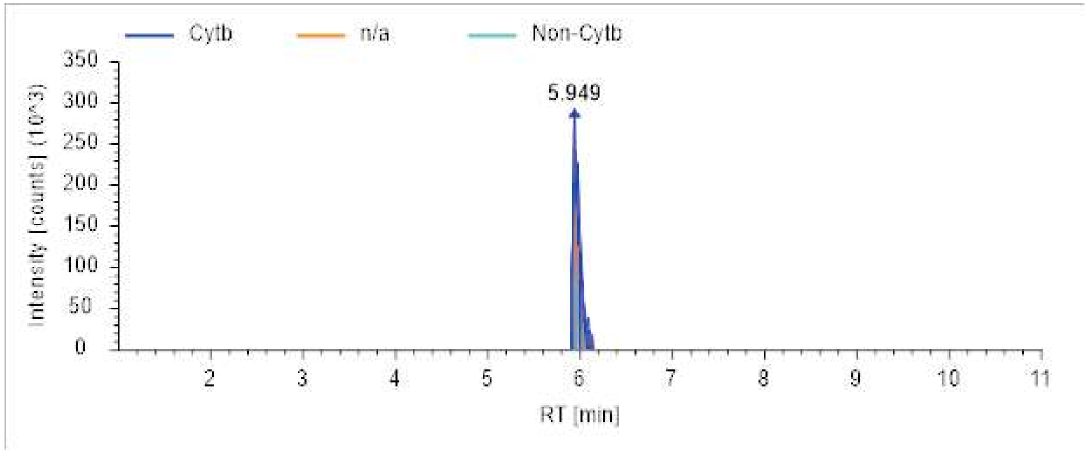

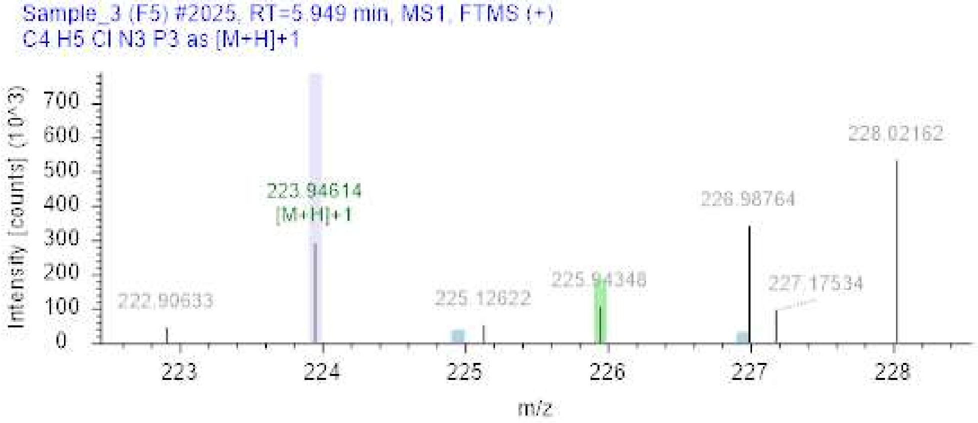

Compounds

17-Dec-2025 1:28

File name: 02_Cytb signature generation-(1)

Study: 02_Cytb signature generation

Structure	Name	RT [min]	Formula	Calc. MW	Group Areas
		5.98	C5 H7 O2 P S	161.99050	7.12e6 1.53e6

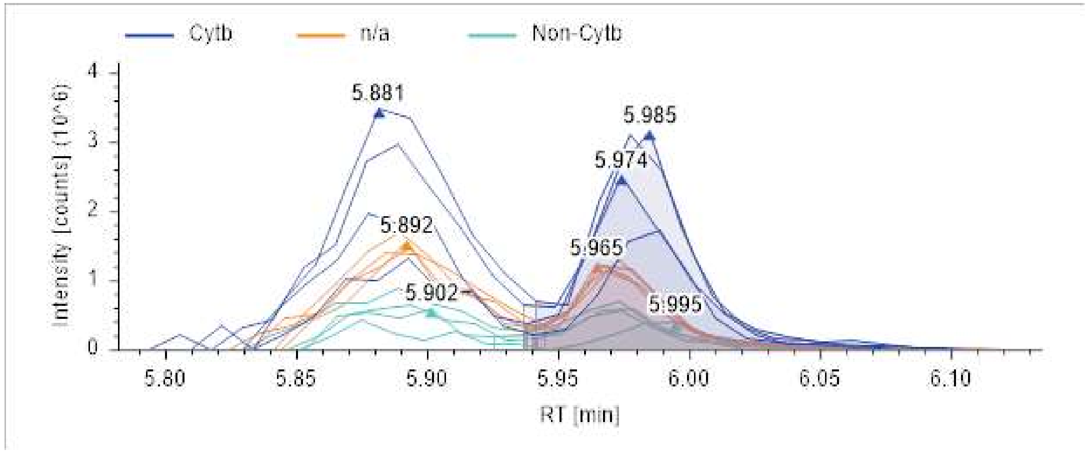

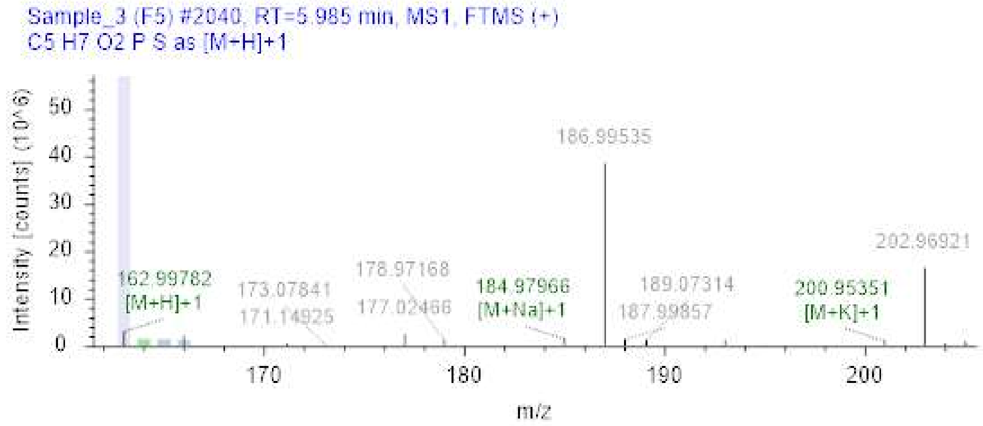

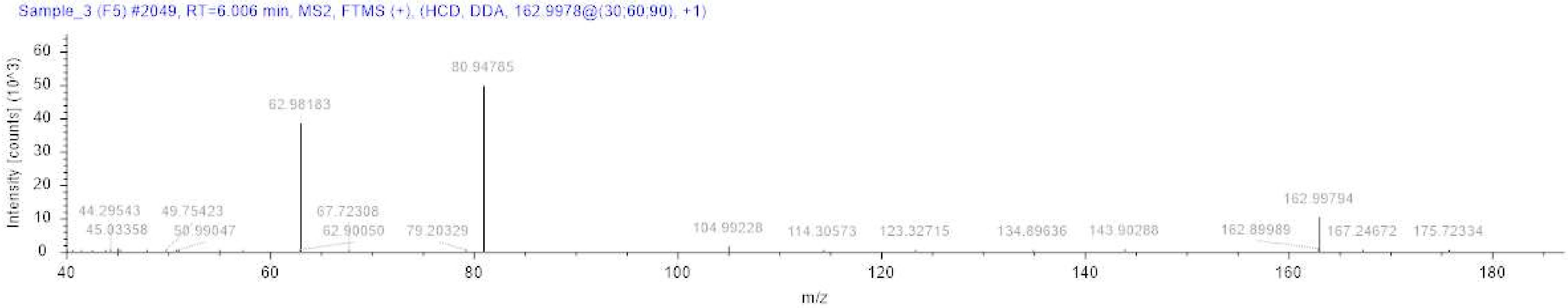

Compounds

17-Dec-2025 1:28

File name: 02_Cytb signature generation-(1)

Study: 02_Cytb signature generation

Structure	Name	RT [min]	Formula	Calc. MW	Group Areas
		5.98	C8 H3 O P S	177.96434	3.18e6 / 8.28e5

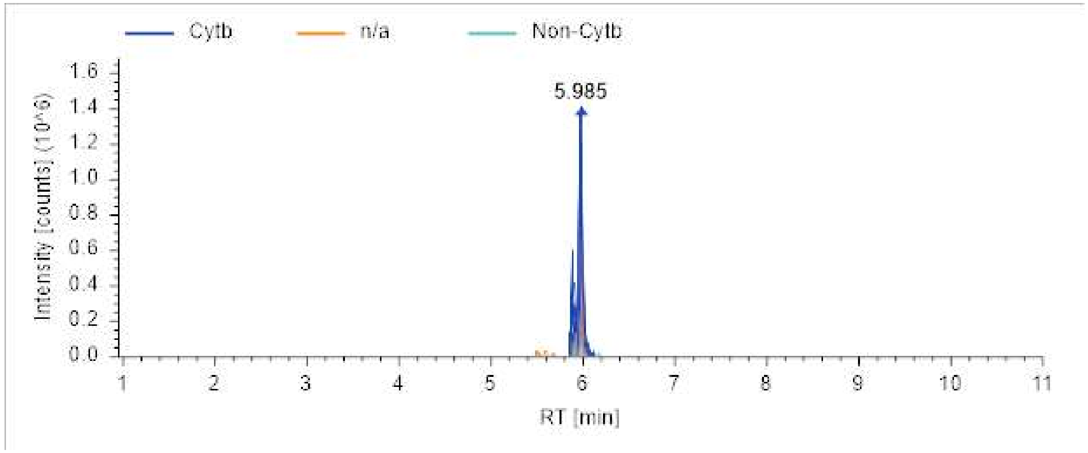

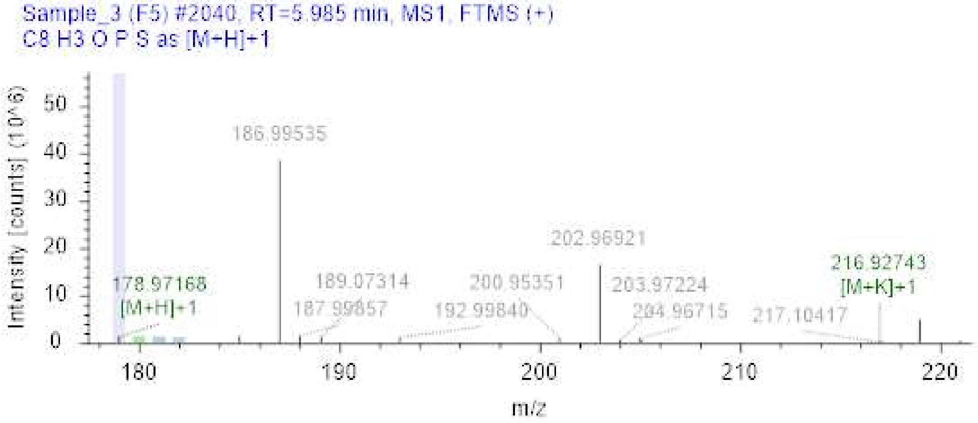

Compounds

17-Dec-2025 1:28

File name: 02_Cytb signature generation-(1)

Study: 02_Cytb signature generation

Structure	Name	RT [min]	Formula	Calc. MW	Group Areas
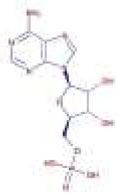	Adenosine monophosphate	6.14	C10 H14 N5 O7 P	347.06330	6.64e6 / 1.93e6

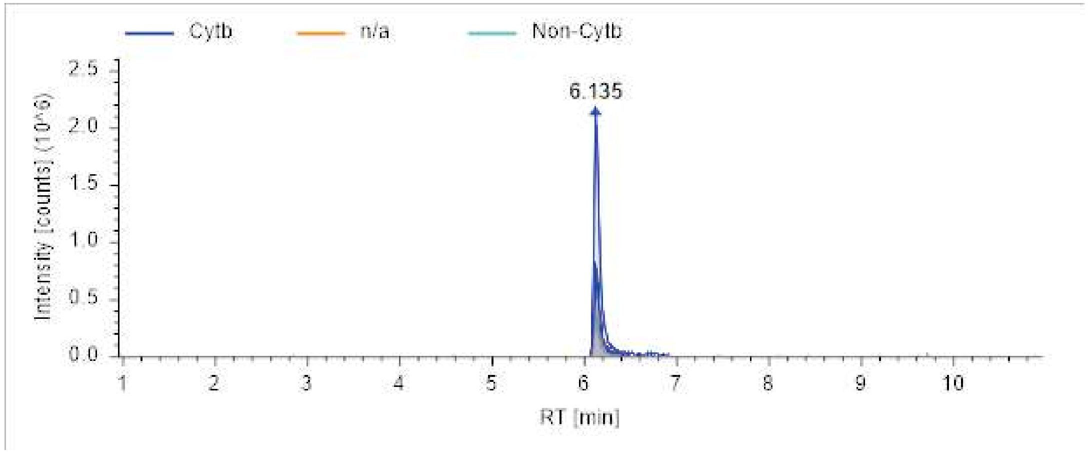

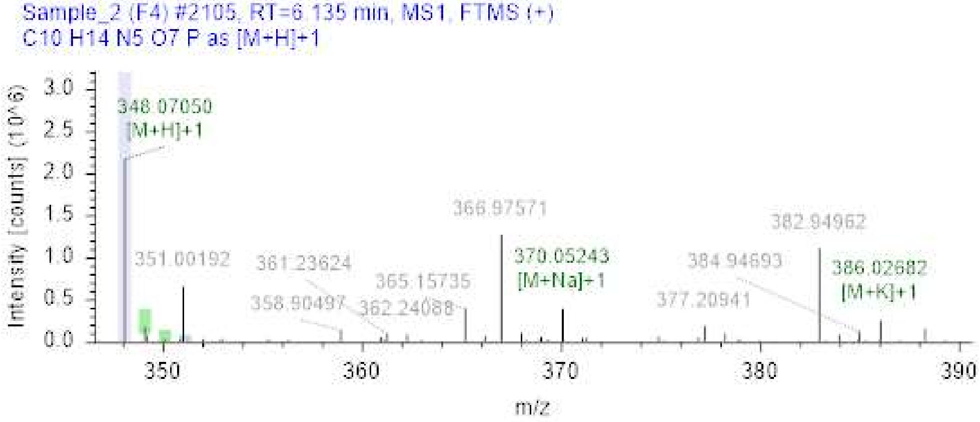

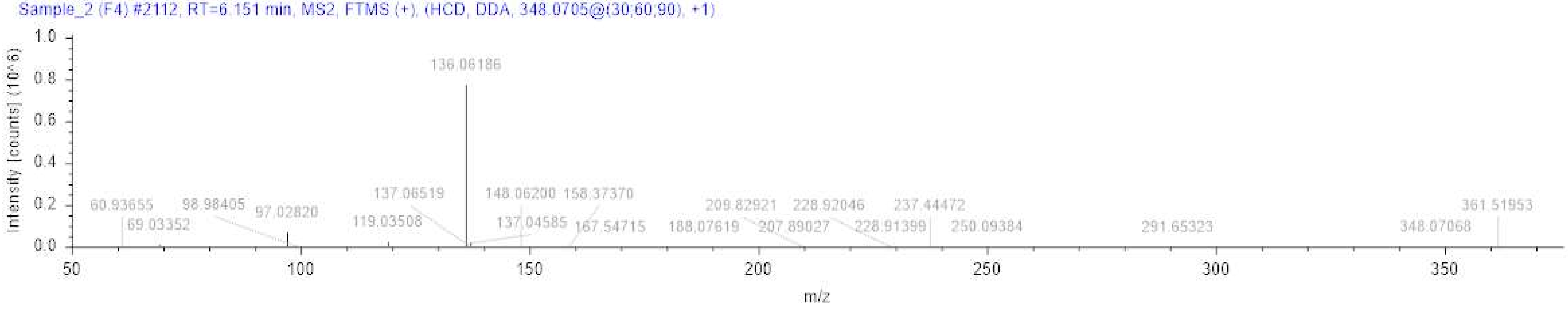

Compounds

17-Dec-2025 1:28

File name: 02_Cytb signature generation-(1)

Study: 02_Cytb signature generation

Structure	Name	RT [min]	Formula	Calc. MW	Group Areas
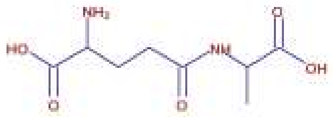	gamma-Glutamylalanine	6.49	C8 H14 N2 O5	218.09009	4.82e6 / 2.03e6

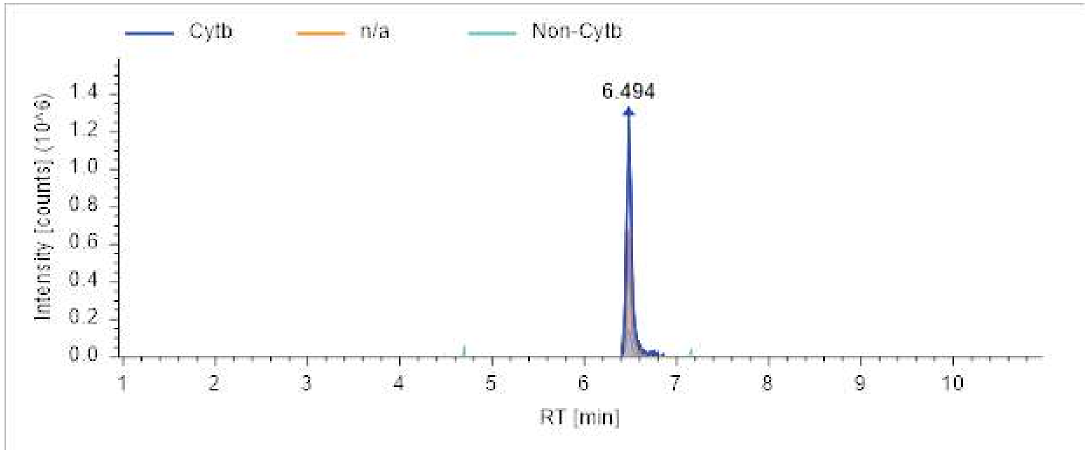

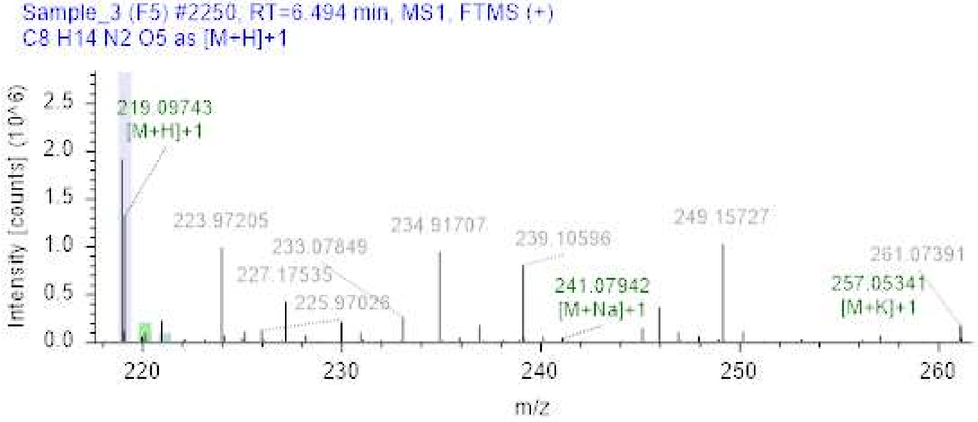

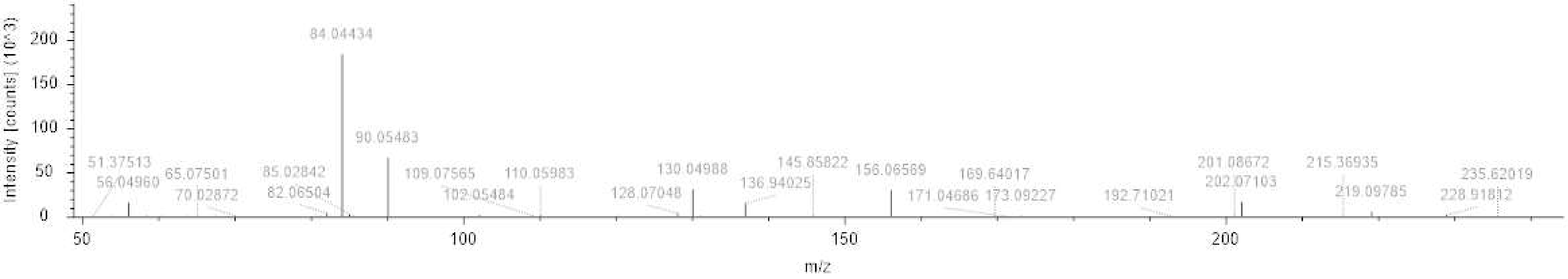

Compounds

17-Dec-2025 1:28

File name: 02_Cytb signature generation-(1)

Study: 02_Cytb signature generation

Structure	Name	RT [min]	Formula	Calc. MW	Group Areas	
		6.51	C11 H21 N2 O4 P3	338.07045	7.69e4	2.69e5

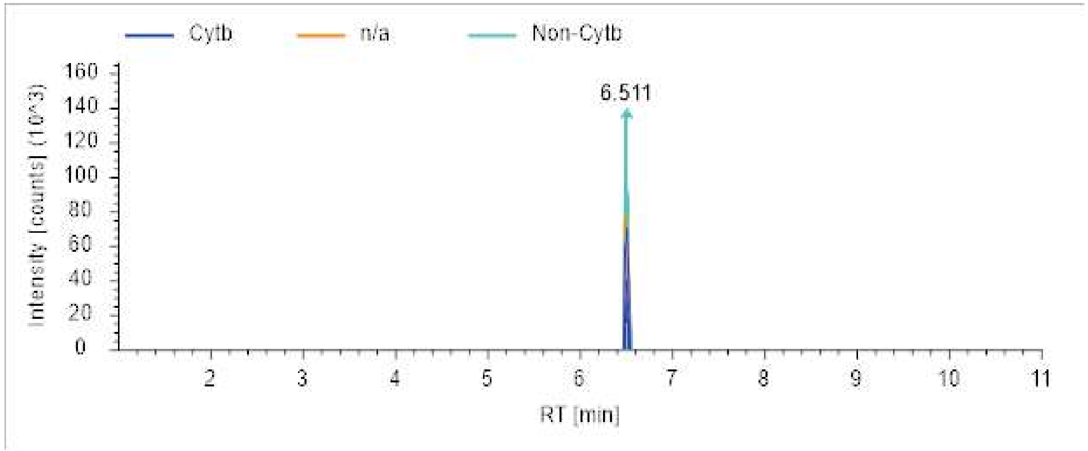

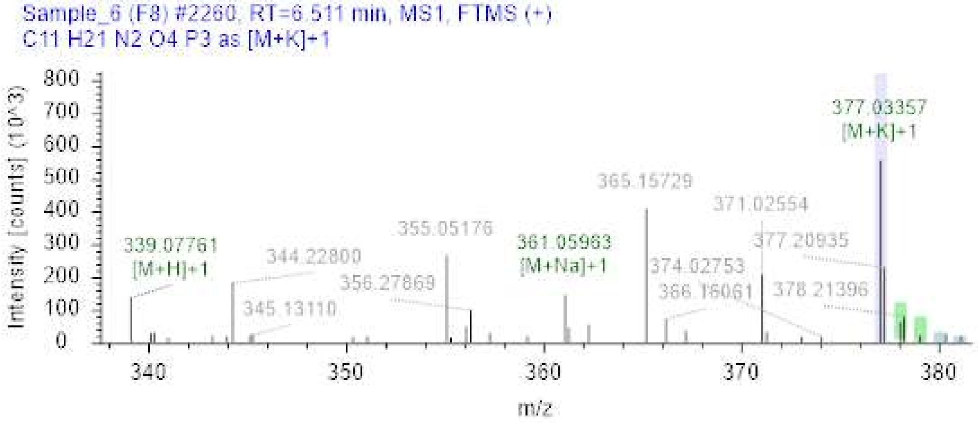

Structure	Name	RT [min]	Formula	Calc. MW	Group Areas
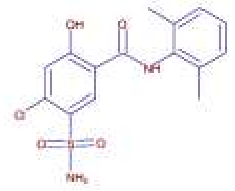	Xipamide	6.51	C15 H15 Cl N2 O4 S	354.04446	1.16e6 / 2.62e6

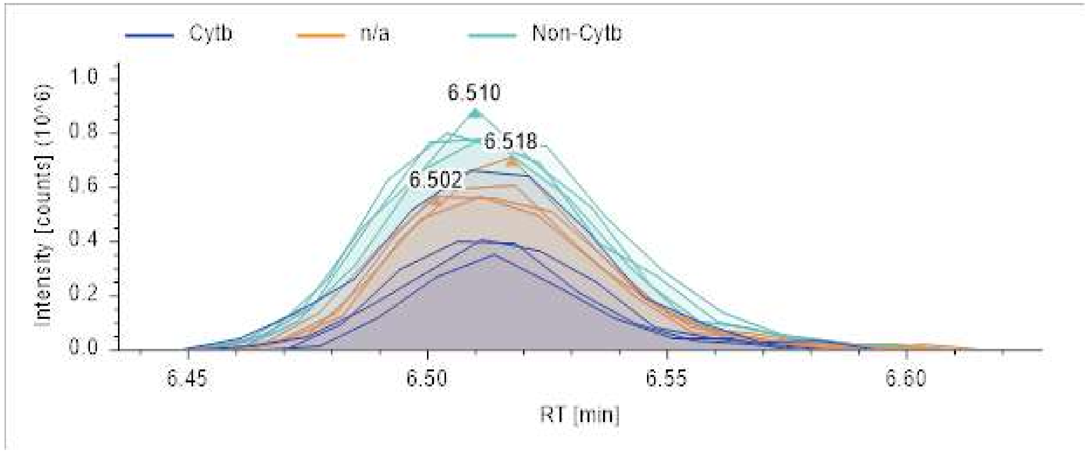

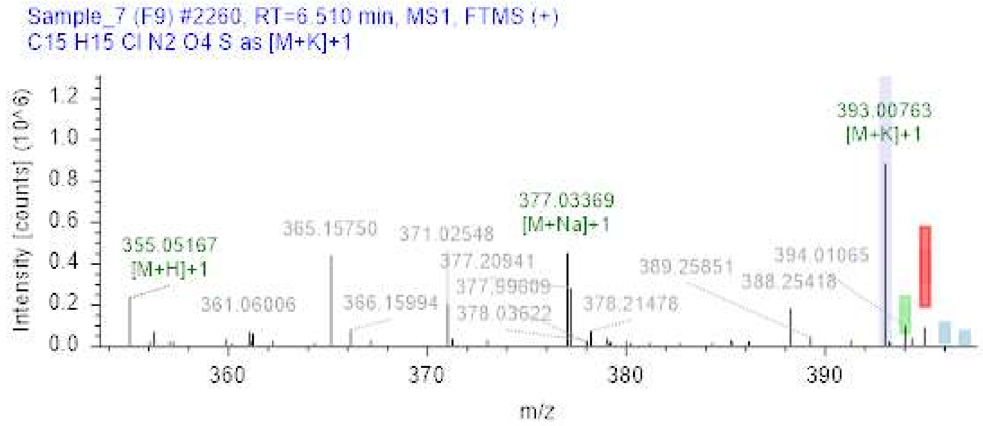

Compounds

17-Dec-2025 1:28

File name: 02_Cytb signature generation-(1)

Study: 02_Cytb signature generation

Structure	Name	RT [min]	Formula	Calc. MW	Group Areas	
		6.51	C25 H14 N O13 P S	598.99238	2.64e5	6.13e5

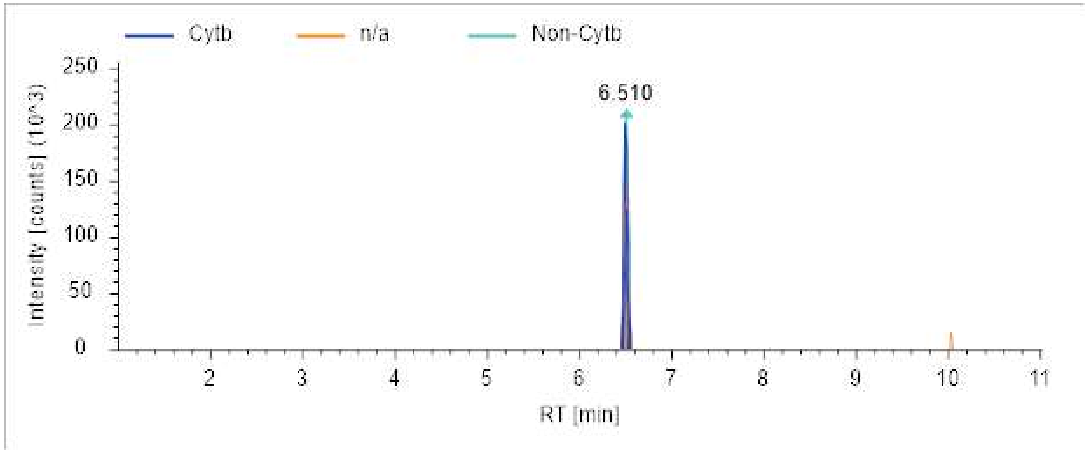

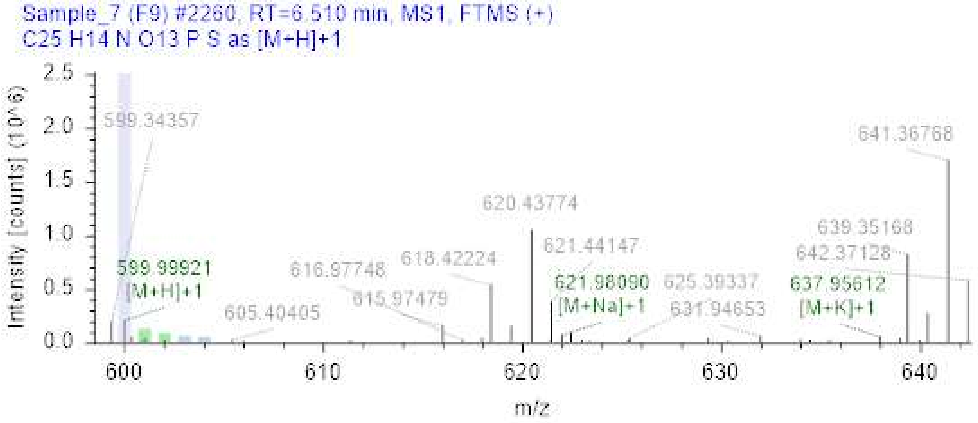

Compounds

17-Dec-2025 1:28

File name: 02_Cytb signature generation-(1)

Study: 02_Cytb signature generation

Structure	Name	RT [min]	Formula	Calc. MW	Group Areas
		6.51	C8 H12 Cl O16 P	429.95617	9.55e5 / 1.98e6

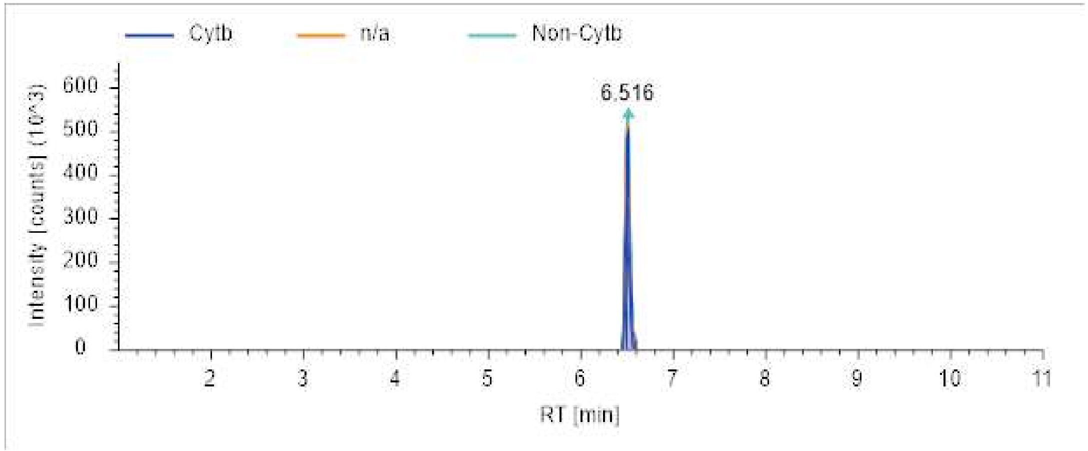

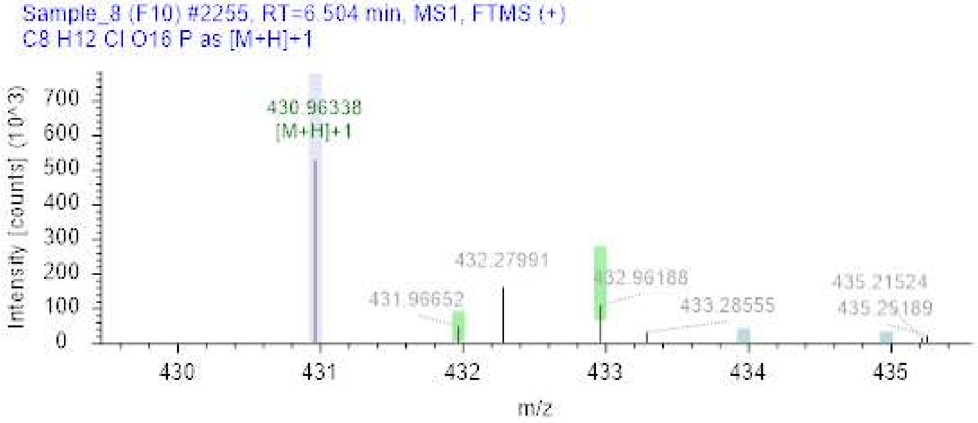

Compounds

17-Dec-2025 1:28

File name: 02_Cytb signature generation-(1)

Study: 02_Cytb signature generation

Structure	Name	RT [min]	Formula	Calc. MW	Group Areas	
	INOSINE-MONOPHOSPHATE	6.52	C10 H13 N4 O8 P	348.04733	2.42e6	5.34e4

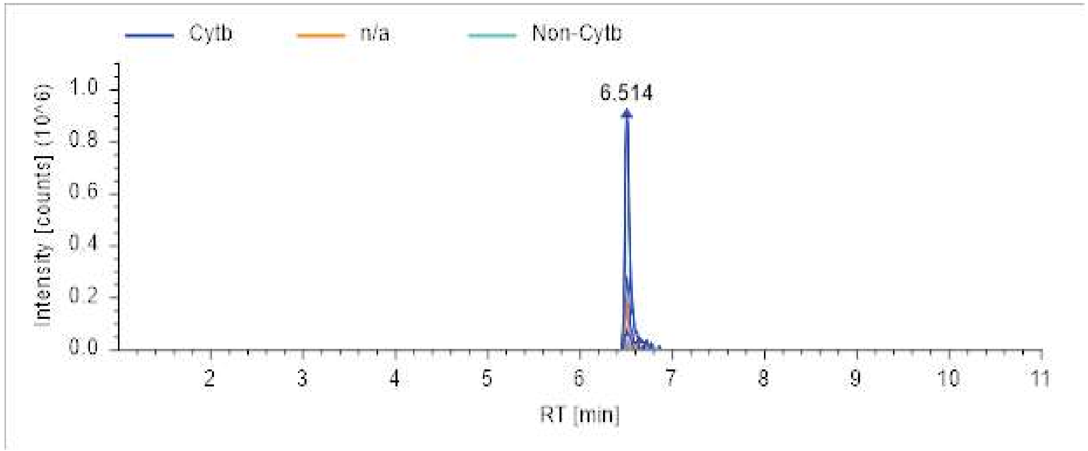

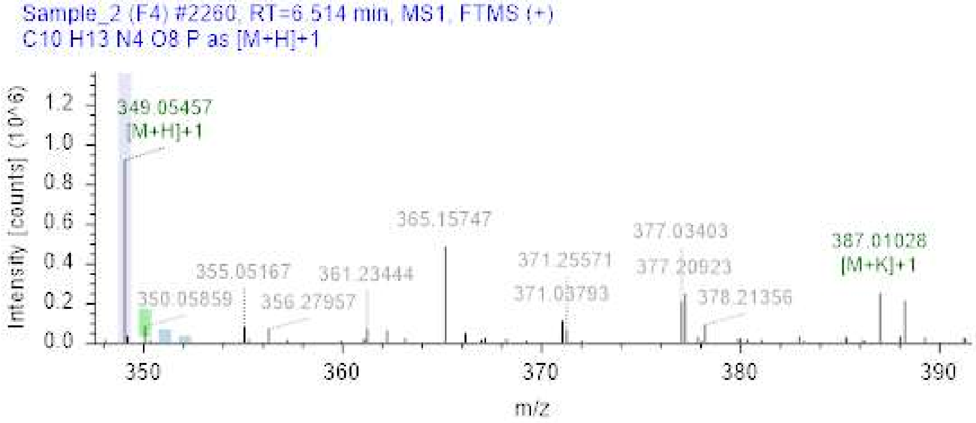

Compounds

17-Dec-2025 1:28

File name: 02_Cytb signature generation-(1)

Study: 02_Cytb signature generation

Structure	Name	RT [min]	Formula	Calc. MW	Group Areas	
	ASPARTATE	6.58	C4 H7 N O4	133.03752	1.28e6	2.90e6

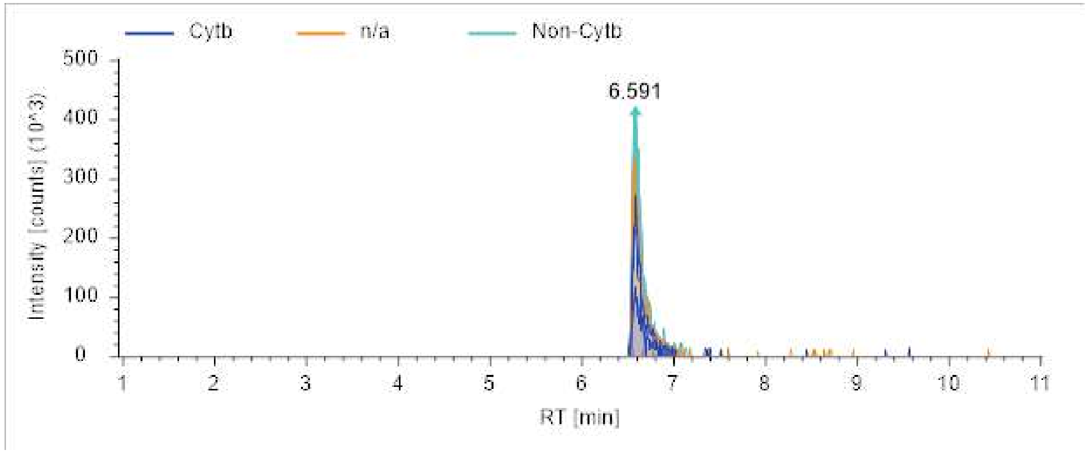

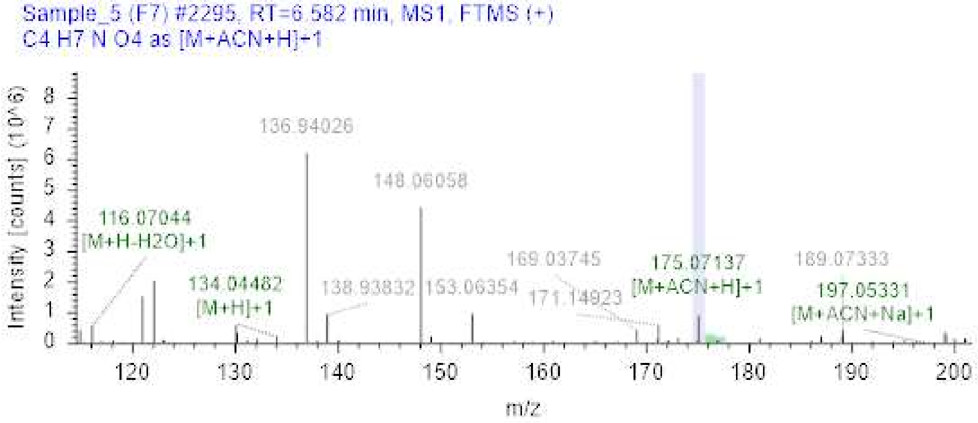

Compounds

17-Dec-2025 1:28

File name: 02_Cytb signature generation-(1)

Study: 02_Cytb signature generation

Structure	Name	RT [min]	Formula	Calc. MW	Group Areas	
		6.59	C3 H6 N3 P3 S	208.94910	2.91e5	7.37e5

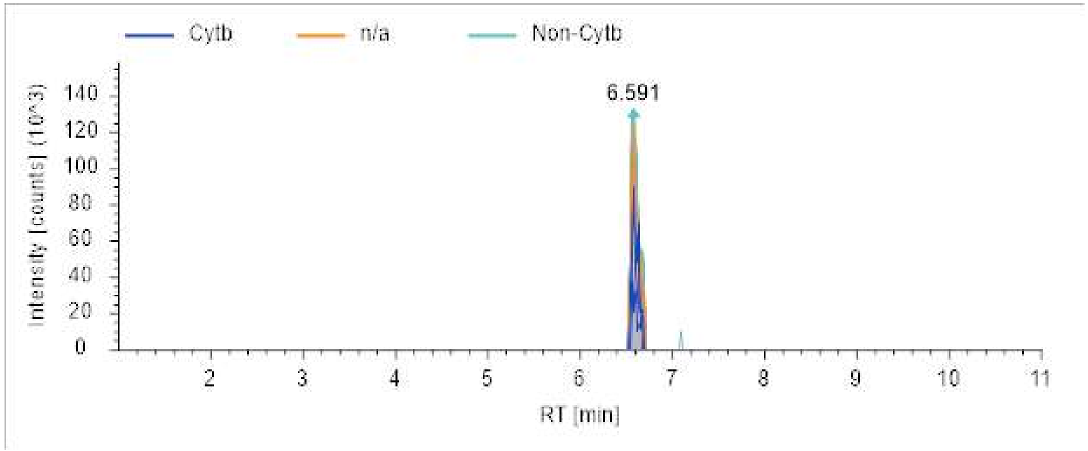

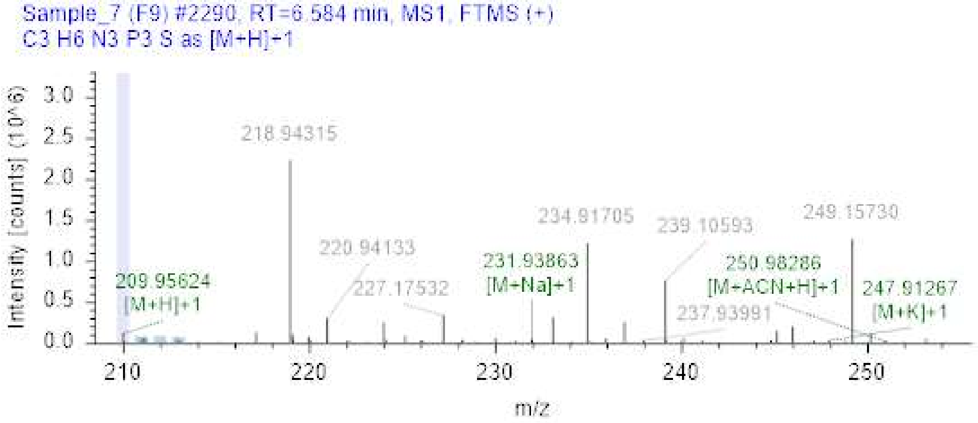

Compounds

17-Dec-2025 1:28

File name: 02_Cytb signature generation-(1)

Study: 02_Cytb signature generation

Structure	Name	RT [min]	Formula	Calc. MW	Group Areas
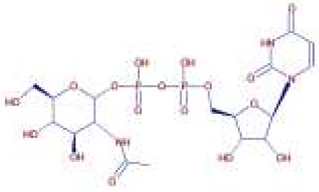	UDP-GlcNAc	6.92	C17 H27 N3 O17 P2	607.08308	4.62e6 / 9.72e6

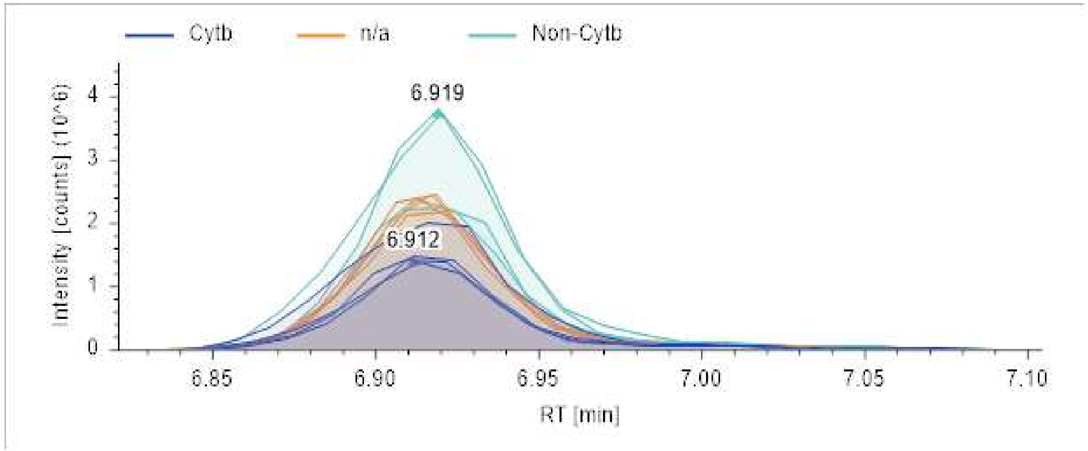

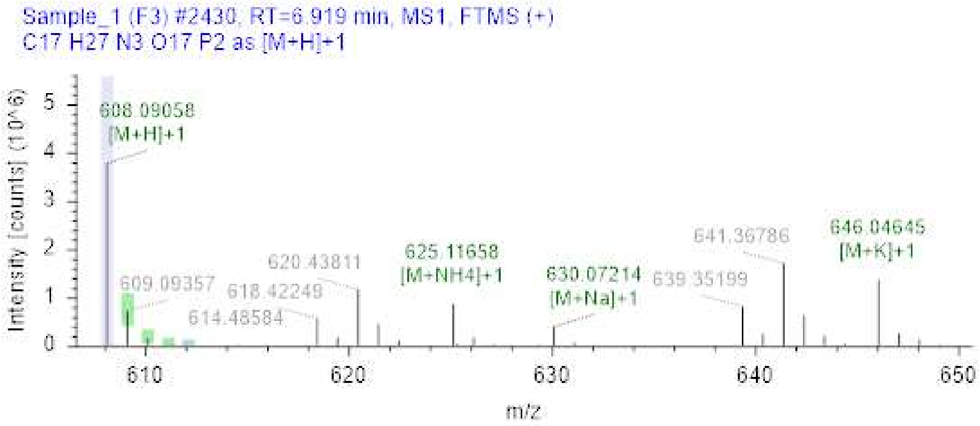

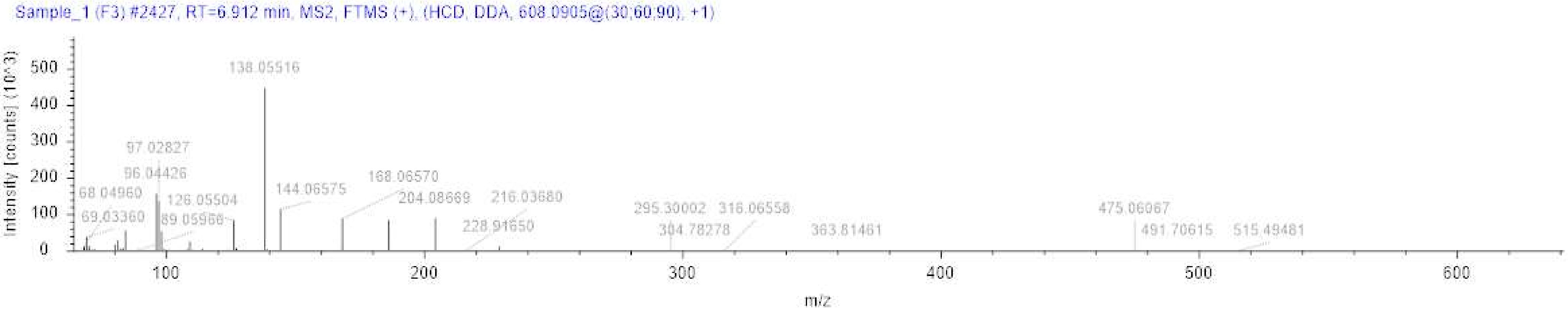

Compounds

17-Dec-2025 1:28

File name: 02_Cytb signature generation-(1)

Study: 02_Cytb signature generation

Structure	Name	RT [min]	Formula	Calc. MW	Group Areas	
		6.92	C21 H22 N3 O15 P S3	682.99485	4.22e5	8.97e5

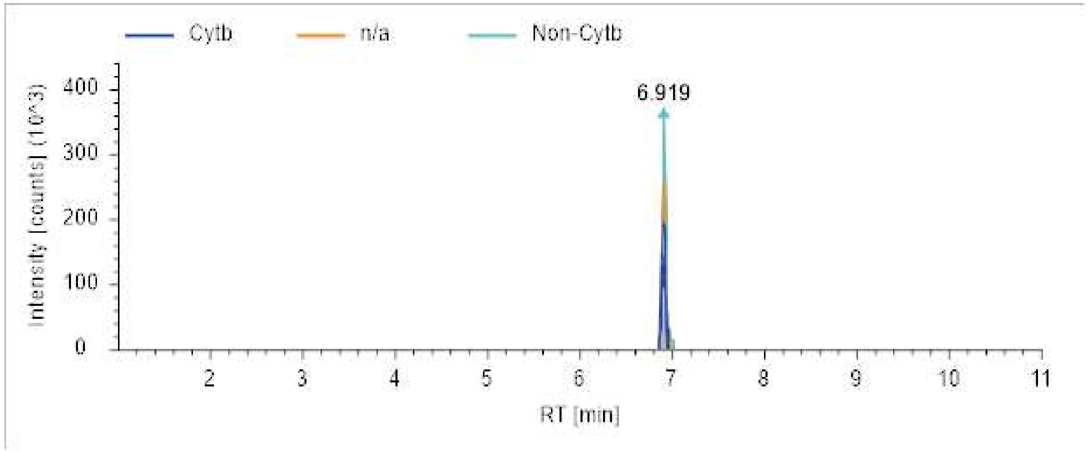

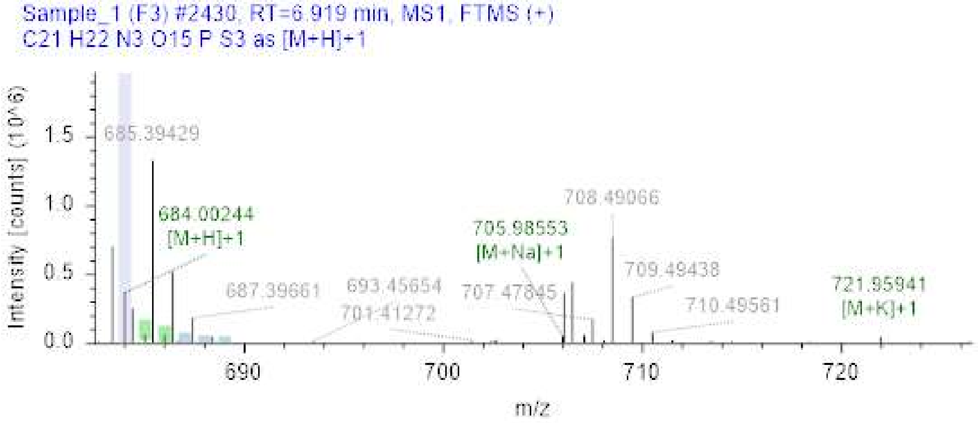

Compounds

17-Dec-2025 1:28

File name: 02_Cytb signature generation-(1)

Study: 02_Cytb signature generation

Structure	Name	RT [min]	Formula	Calc. MW	Group Areas	
		6.92	C39 H14 N3 O3 P S2	667.02084	2.49e5	5.06e5

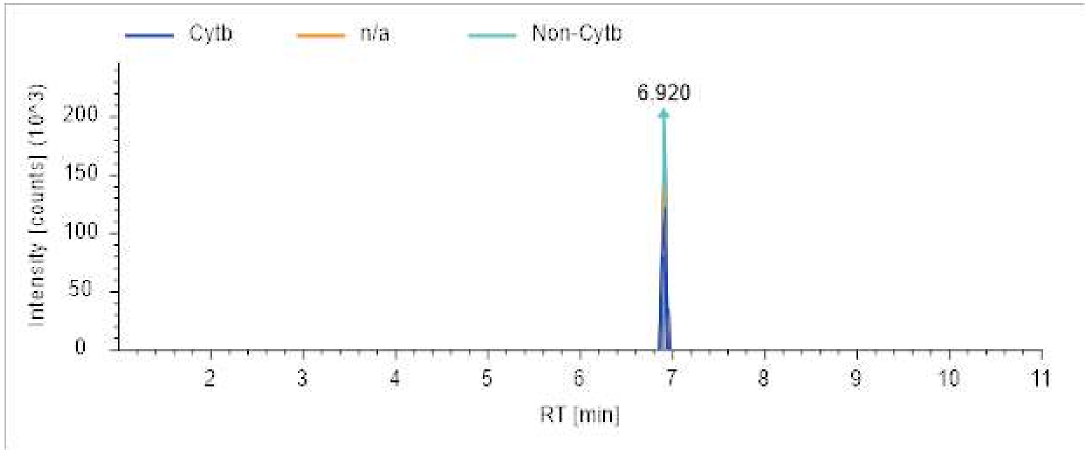

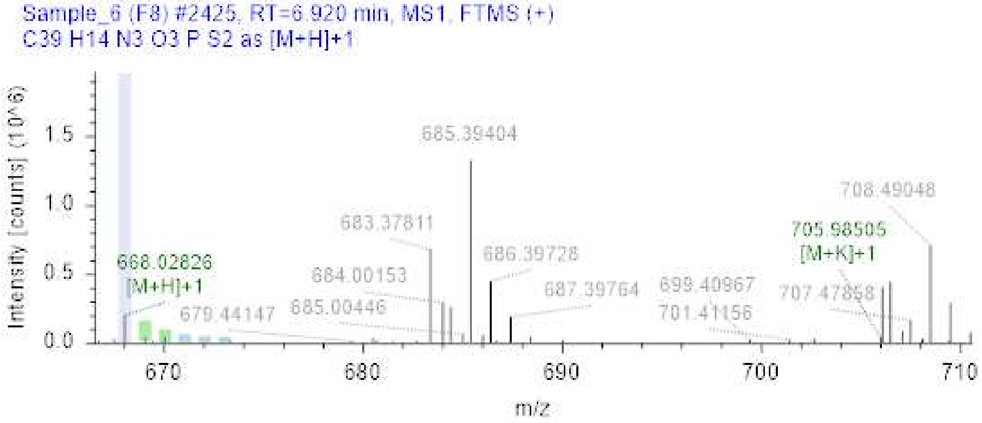

Compounds

17-Dec-2025 1:28

File name: 02_Cytb signature generation-(1)

Study: 02_Cytb signature generation

Structure	Name	RT [min]	Formula	Calc. MW	Group Areas
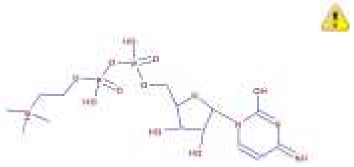	Citicoline	6.95	C14 H26 N4 O11 P2	488.10803	1.02e6 / 3.96e5

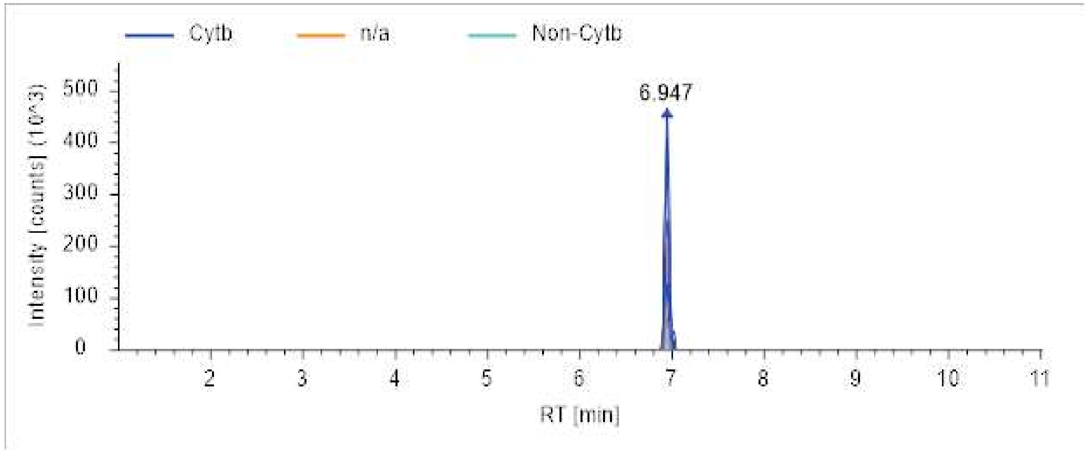

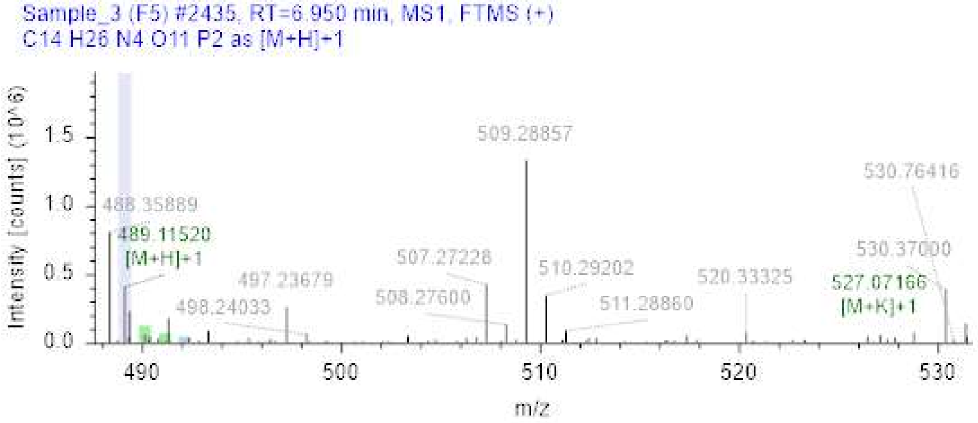

Compounds

17-Dec-2025 1:28

File name: 02_Cytb signature generation-(1)

Study: 02_Cytb signature generation

Structure	Name	RT [min]	Formula	Calc. MW	Group Areas
	ADENOSINE 5'-DIPHOSPHATE	7.41	C10 H15 N5 O10 P2	427.02981	2.16e7 9.88e6

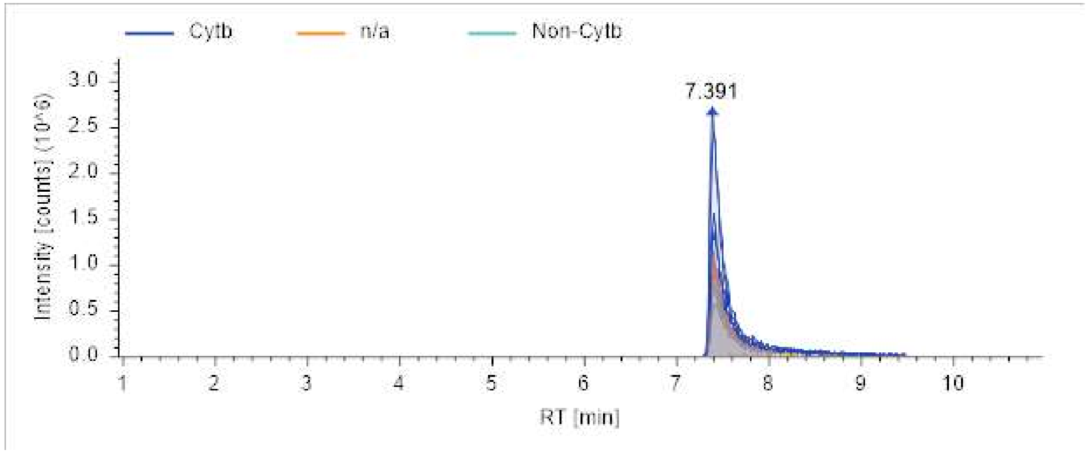

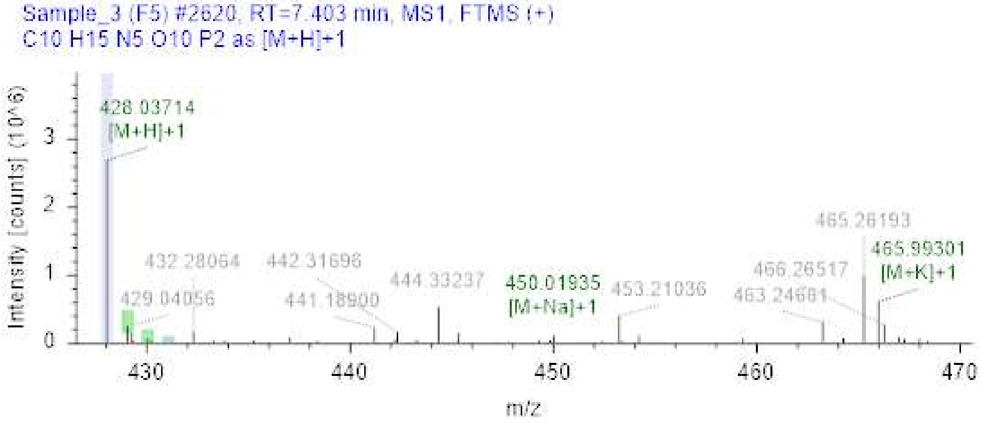

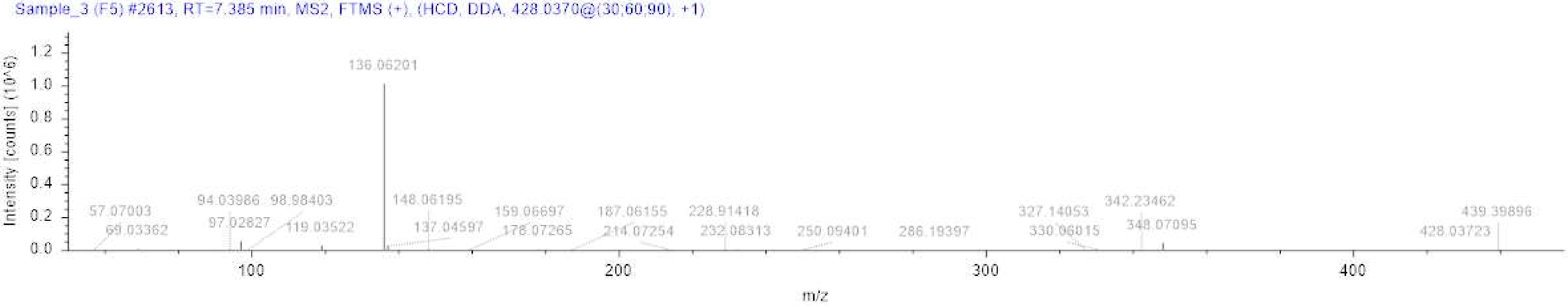

Compounds

17-Dec-2025 1:28

File name: 02_Cytb signature generation-(1)

Study: 02_Cytb signature generation

Structure	Name	RT [min]	Formula	Calc. MW	Group Areas
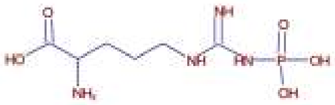	Phosphoarginine	7.82	C6 H15 N4 O5 P	254.07806	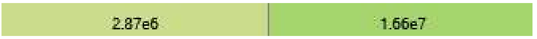

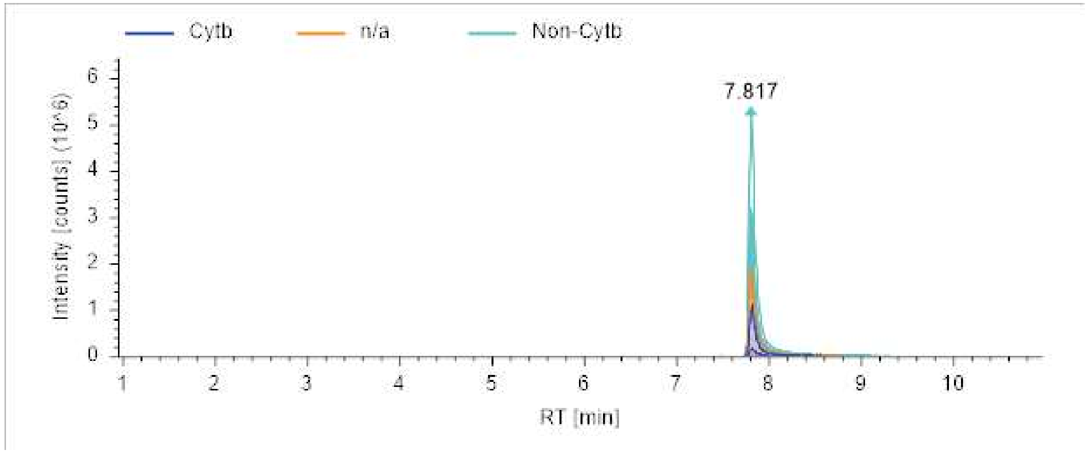

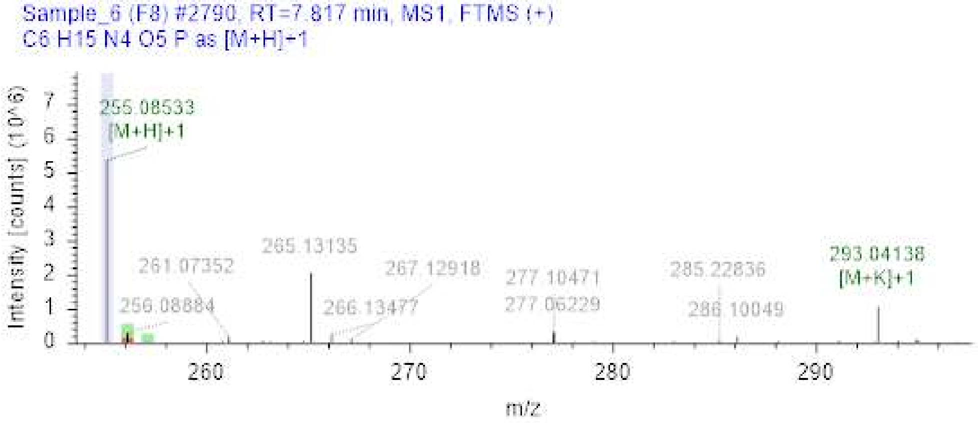

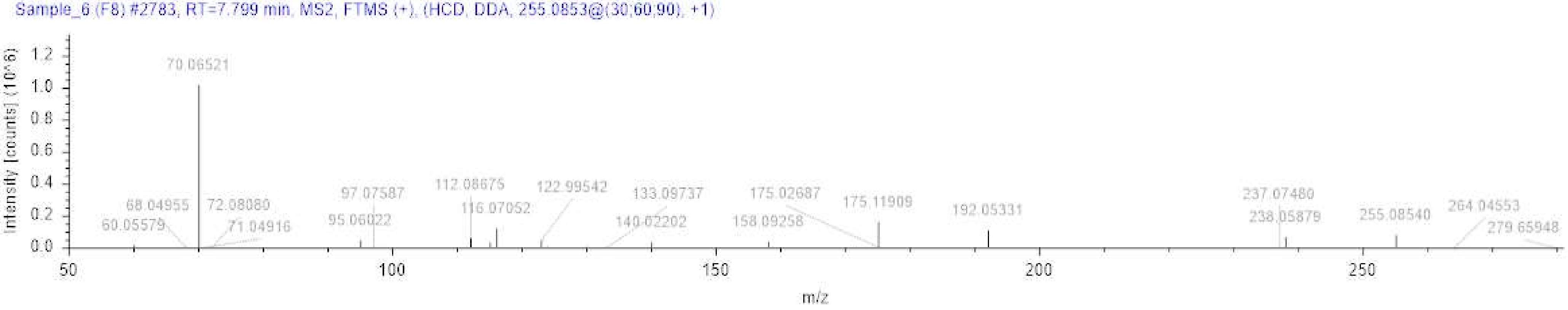

Structure	Name	RT [min]	Formula	Calc. MW	Group Areas
